# Supplementary material for: Upconversion NIR-II fluorophores for mitochondria-targeted cancer imaging and photothermal therapy
Source: Nat Commun. 2020 Dec 3;11:6183. doi: 10.1038/s41467-020-19945-w (PMC7713230; doi:10.1038/s41467-020-19945-w)
Supplement: Supplementary file 1 — Supporting Information [file 41467_2020_19945_MOESM1_ESM.pdf]

Supplementary Information

Upconversion NIR-II Fluorophores for Mitochondria-Targeted Cancer Imaging and Photothermal Therapy

Zhou *et. al.*

Supplementary Materials

Materials and Methods

All chemicals were purchased from commercial sources (such as Aldrich, Energy Chemical, TCI and Chinapeptides) without further purification. MitoTracker Green (Life Technologies) was re-suspended in dimethyl sulfoxide (DMSO; Sigma) and used at a final concentration of 200 nM. Antibodies used in this work included the following: recombinant Anti-Cytochrome C antibody (ab133504, abcam); secondary Alexa-647-conjugated goat anti-rabbit antibody, (A27040, Life technologies); anti-caspase 3 (9662S, Cell Signaling), anti-caspase 9 (9504S, Cell Signaling), anti-cleaved caspase 3 (9664S, Cell Signaling), anti-cleaved caspase 9 (9509S, Cell Signaling); recombinant anti-Smac/Diablo antibody (ab32023,abcam); anti-Endo G antibody (ab9647, abcam); recombinant anti-AIF antibody (ab32516, abcam); recombinant anti-Cytochrome C antibody (ab133504, Abcam); secondary Alexa-647-conjugated Goat anti-rabbit antibody (A27040, Life technologies); HRP conjugated goat anti-rabbit IgG H&L (HRP) (ab6721, abcam) and anti-β-actin antibody (ab179467, Abcam). Chemical structures were drawn by Chemdraw 14. The ^{1}H spectra were acquired on a Bruker 400 MHz, 500 MHz or 600MHz magnetic resonance spectrometer and ^{13}C spectra were obtained on the same instruments at 100 MHz, 125 MHz or 150 MHz. Multiplicities were reported as s (singlet), d (doublet), t (triplet), q (quartet), m (multiplet), dd (doublet of doublet) etc. TLC analysis was performed on silica gel plates and column chromatography was conducted over silica gel (mesh 200-300) from the Qingdao Ocean Chemicals. UV-Vis absorbance of the probes was recorded on a PerkinElmer Lambda 25 UV-Vis spectrophotometer. NIR fluorescence spectrum was recorded on an Applied Nano Fluorescence spectrometer at room temperature with an excitation laser source of 785 nm. Confocal laser scanning microscope (CLSM) was recorded under a FV1200 CLSM (Olympus) consisting of a Coherent ULTRA near infrared tunable Titanium Sapphire laser (690-1040 nm), FLUOVIEW FV 1000 (Olympus) and Leica SPE. Cellular uptake and DCFH-DA assay images were obtained by Zeiss Axiovert 200 fluorescence microscopy. Bioluminescence or fluorescence intensity in ATP detection, JC-1 assay, MPT detection, and caspase multiplex activity assay was collected from microplate reader (TECAN, Infinite M200 Pro). The sequence of PT is PPSHTPT. Preparative high performance liquid chromatography (HPLC) was performed on a Dionex HPLC System with a UV-Vis detection. A reversed-phase C4 (Ultimate, 5 μm, 4.6 × 250 mm) column was used for semi-preparation (mobile phase: water/acetonitrile with 0.05 % TFA). Maldi-TOF mass spectra was obtained on Applied Biosystems 4700 Proteomics Analyzer.

Synthesis and characterization

Synthesis of compound ***5a*** and *1-(4-hydroxyphenyl)ethan-1-one*: This compound was prepared by a modified procedure according to previous reports. Compound **4** (1.36 g, 10 mmol) was dissolved in 20 mL of ethanol, and the 20% KOH (20 mL) solution was added and reacted at room temperature for 10 min. Then benzaldehyde (1.16 g, 11 mmol) was added into the mixture and the reaction stirred at room temperature overnight. After cooling to room temperature, the reaction solution was adjusted to pH 3 with 2 M aq. HCl solution, and the yellow solid that formed was collected by filtration. The product

1-(4-hydroxyphenyl)ethan-1-one (1.83 g, yield 82%) can be used in the next step without further purification (**5a**, yield 80%).

Characterization of **5a** ^{1}H NMR (400 MHz, $CDCl_3$) δ 8.05 (s, 2H), 7.83 (d, *J* = 15.7 Hz, 1H), 7.64 (dd, *J* = 6.6, 2.8 Hz, 2H), 7.62 – 7.46 (m, 4H), 7.45 – 7.36 (m, 3H); ^{13}C NMR (101 MHz, $CDCl_3$) δ 190.5, 144.9, 138.2, 134.9, 132.9, 130.6, 129.0, 128.7, 128.6, 128.5, 122.1.

Characterization of **1-(4-hydroxyphenyl)ethan-1-one:** ^{1}H NMR (400 MHz, Acetone-d_6) δ 9.38 (s, 1H), 8.15 – 8.09 (m, 2H), 7.90 (d, *J* = 15.6 Hz, 1H), 7.86 – 7.81 (m, 2H), 7.77 (d, *J* = 15.6 Hz, 1H), 7.51 – 7.43 (m, 3H), 7.03 – 6.97 (m, 2H); ^{13}C NMR (101 MHz, Acetone-d_6) δ 187.1, 161.9, 142.8, 135.4, 131.0, 130.2, 130.2, 128.9, 128.5, 122.0, 115.3.

Synthesis of compound ***5b**:* To a solution of compound 1-(4-hydroxyphenyl)ethan-1-one (1.12 g, 5 mmol) and K_2CO_3 (1.52 g, 11 mmol) in acetone (10 mL) was stirred for 10 min, then 3-bromopropyne (0.654 g, 5.5 mmol) was added into the mixture, it was heated to reflux and it was stirring for 4 h. Then the mixture was filtered and filtrate was concentrated under reduced pressure to give the product (1.27 g, 97% yield). It also can be used in the next step without further purification.

Characterization of ***5b***: ^{1}H NMR (400 MHz, Acetone-d_6) δ 8.24 – 8.18 (m, 2H), 7.92 (d, *J* = 15.6 Hz, 1H), 7.88 – 7.83 (m, 2H), 7.79 (d, *J* = 15.6 Hz, 1H), 7.52 – 7.43 (m, 3H), 7.21 – 7.15 (m, 2H), 4.95 (d, *J* = 2.4 Hz, 2H), 3.19 (t, *J* = 2.4 Hz, 1H); ^{13}C NMR (101 MHz, Acetone-d_6) δ 187.3, 161.5, 143.3, 135.3, 131.7, 130.7, 130.3, 128.9, 128.6, 121.9, 114.7, 78.3, 76.7, 55.6.

Synthesis of compounds ***6a*** *and* ***6b***: Cyclopentanone (1.2 g, 14.3 mmol) and tetrahydropyrrole (1.02 g, 14.3 mmol) was dissolved in benzene (12 mL), the solution was heated to 100 °C and stirred for 4 h. After cooling to room temperature, benzene was evaporated under reduced pressure. Then compound **5b** (2.5g, 9.53 mmol) and dry dioxane were added into the mixture, the solution was heated under reflux for 6 h. Water was added into the mixture and it was extracted with EtOAc. After that, organic layer was washed with saturated aqueous brine. The combined organic layers were dried with anhydrous magnesium sulfate, filtered and concentrated. Then the solvent was removed in vacuum and the crude product was purified by column chromatography on silica gel to get **6b** 2.17 g (66%); (**6a**, yield 68%).

Characterization of **6a**: ^{1}H NMR (400 MHz, $CDCl_3$) δ 8.05 – 7.95 (m, 2H), 7.57 (t, *J* = 7.3 Hz, 1H), 7.47 (t, *J* = 7.6 Hz, 2H), 7.34 – 7.18 (m, 5H), 3.92 – 3.73 (m, 2H), 3.62 – 3.42 (m, 1H), 2.63 – 2.48 (m, 1H), 2.35 – 2.08 (m, 2H), 2.04 – 1.88 (m, 1H), 1.90 – 1.73 (m, 2H), 1.73 – 1.60 (m, 1H); ^{13}C NMR (101 MHz, $CDCl_3$) δ 220.7, 199.1, 142.3, 137.0, 133.1, 128.6, 128.5, 128.2, 126.8, 53.1, 41.0, 40.9, 39.8, 27.1, 20.6.

Characterization of **6b**: ^{1}H NMR (400 MHz, $CDCl_3$) δ 8.02 – 7.95 (m, 2H), 7.28 (m, 1H), 7.25 (m, 2H), 7.25 – 7.16 (m, 2H), 7.03 – 6.98 (m, 2H), 4.74 (d, *J* = 2.5 Hz, 2H), 3.82 – 3.68 (m, 2H), 3.48 – 3.35 (m, 1H), 2.55 (t, *J* = 2.4 Hz, 1H), 2.29 – 2.19 (m, 1H), 2.13 (m, 1H), 1.92 (m, 1H), 1.77 (m, 2H), 1.71 – 1.58 (m, 2H); ^{13}C NMR (101 MHz, $CDCl_3$) δ 220.8, 197.7, 161.4, 142.5, 131.0, 130.5, 128.6, 128.6, 126.8, 114.7, 77.9, 76.3, 55.9, 53.2, 41.3, 40.7, 39.8, 27.2, 20.7.

Synthesis of compounds ***7a*** *and* ***7b***: Compound **6b** (1.2 g, 3.46 mmol) was dissolved in ether (10 mL) and stirred for 10 min, thioacetic acid (0.659 g, 8.66 mmol) and boron trifluoride ether (2.46 g, 17.32 mmol)

were added into the mixture, then the solution was heated under reflux for 8 h. After cooling to room temperature, the reaction mixture was quenched by water, excess ether was added into the solution. Then the mixture was stirred at room temperature to precipitate the pale yellow solid **7b** without purification. 0.4 g; Yield: 50%. (**7a**, yield 62%)

Characterization of **7a**: ^{1}H NMR (400 MHz, Acetonitrile-d_3) δ 8.71 (s, 1H), 7.99 (d, *J* = 7.5 Hz, 2H), 7.82 (dd, *J* = 7.6, 2.1 Hz, 2H), 7.78 (d, *J* = 7.3 Hz, 1H), 7.74 (s, 1H), 7.73 – 7.67 (m, 4H), 3.71 (t, *J* = 7.5 Hz, 2H), 3.39 (t, *J* = 7.5 Hz, 2H), 2.39 (p, *J* = 7.5 Hz, 2H); ^{13}C NMR (101 MHz, CD_3CN) δ 176.4, 165.8, 159.6, 150.6, 137.0, 134.1, 133.8, 133.2, 131.8, 130.3, 129.9, 129.3, 128.5, 38.4, 34.2, 24.7.

Characterization of **7b**: ^{1}H NMR (400 MHz, Acetonitrile-d_3) δ 8.63 (s, 1H), 8.01 (d, *J* = 8.9 Hz, 1H), 7.80 (dd, *J* = 7.6, 2.0 Hz, 2H), 7.73 – 7.65 (m, 3H), 7.29 (d, *J* = 9.0 Hz, 2H), 4.92 (d, *J* = 2.4 Hz, 2H), 3.66 (t, *J* = 7.5 Hz, 2H), 3.34 (t, *J* = 7.5 Hz, 2H), 2.94 (t, *J* = 2.4 Hz, 1H), 2.37 (p, *J* = 7.5 Hz, 2H); ^{13}C NMR (101 MHz, Acetonitrile-d_3) δ 175.3, 166.6, 162.8, 160.3, 150.4, 138.0, 133.4, 132.6, 131.1, 130.1, 130.1, 128.1, 117.4, 78.7, 77.7, 57.0, 39.1, 35.0, 25.4; HRMS (ESI) Calcd for: $C_{23}H_{19}OS^+$ ($[M\text{-}BF4]^+$): 343.1151, found: 343.1155.

*Synthesis of **2, 4-iodo-N, N-dimethylaniline***: To a mixture of *N, N*-dimethylaniline (600 mg, 4.95 mmol) and solution of $NaHCO_3$ (624 mg, 7.43 mmol), iodine (1.13 g, 4.46 mmol) was slowly added into the solution and stirred at room temperature for 2 h. Then the mixture was quenched by sodium thiosulfate and extracted with EtOAc (3 × 25 mL). The combined organic layer was washed with brine, and dried over anhydrous Na_2SO_4. Then the solvent was removed *in vacuum* and the crude product was purified by column chromatography on silica gel (861 mg, yield 70%).

*Characterization of **2, 4-iodo-N, N-dimethylaniline***: ^{1}H NMR (400 MHz, DMSO-d_6) δ 7.51 – 7.37 (m, 2H), 6.60 – 6.47 (m, 2H), 2.87 (s, 6H); ^{13}C NMR (101 MHz, DMSO-d_6) δ 150.3, 137.5, 115.3, 77.6, 40.3.

*Synthesis of **5-(4-(dimethylamino)phenyl)thiophene-2-carbaldehyde***: To a solution of compound 4-iodo-*N, N*-dimethylaniline and (5-formylthiophen-2-yl)boronic acid in THF (2.5 mL) was bubbled with argon for 5 min. Potassium carbonate (18 mg, 0.125 mmol) in 0.5 mL distilled water and $Pd(PPh_3)_4$ were added to the above reaction mixture under an argon atmosphere. The mixture was heated in an oil bath at 80 °C for 6 h. After cooling to room temperature, the solvent was removed in vacuum. The residue was dissolved in dichloromethane, and the resulting solution was washed with saturated aqueous brine. After drying over anhydrous magnesium sulfate and removal of the solvents under reduced pressure, the crude product was purified by column chromatography to give the yellow solid (yield 65%).

*Characterization of **5-(4-(dimethylamino)phenyl)thiophene-2-carbaldehyde***: ^{1}H NMR (400 MHz, $CDCl_3$) δ 9.84 (s, 1H), 7.70 (d, *J* = 4.0 Hz, 1H), 7.58 (d, *J* = 8.9 Hz, 2H), 7.26 (d, *J* = 4.0 Hz, 1H), 6.73 (d, *J* = 8.9 Hz, 2H), 3.04 (s, 6H); ^{13}C NMR (101 MHz, $CDCl_3$) δ 182.5, 156.1, 151.2, 140.1, 138.2, 127.5, 121.5, 120.8, 112.1, 40.2.

*General Procedures for **3a ~3k** and **H4***: Compound **7** (1 eq), different aldehydes (1.3 eq) and acetic anhydride were added into the round-bottomed flask, the mixture was stirred at 70°C for 2 h. The product was obtained after 2 h microwave chemical reaction; then ether was poured into the solution to precipitate

the solid. The residue was purified by column chromatography to give the product **3a-3f** and **H4** (yield 47-60%).

3a: ^{1}H NMR (400 MHz, Acetonitrile-d_3 and DMSO-d_6) δ 8.68 (s, 1H), 8.09 (d, J = 6.1 Hz, 2H), 7.89 – 7.81 (m, 2H), 7.79 – 7.71 (m, 2H), 7.71 (d, J = 7.5 Hz, 2H), 7.67 (s, 4H), 7.56 (s, 1H), 7.53 (d, J = 7.1 Hz, 2H), 7.48 (m, 1H), 3.66 – 3.44 (m, 2H), 3.42 – 3.32 (m, 2H); ^{13}C NMR (151 MHz, Acetonitrile-d_3 and DMSO-d_6) δ 171.1, 163.5, 160.0, 151.1, 142.9, 137.6, 135.8, 135.5, 135.1, 134.9, 134.0, 132.2, 131.4, 130.9, 130.1, 129.9, 129.8, 129.2, 127.4, 33.5, 30.7.

3b: ^{1}H NMR (400 MHz, Acetonitrile-d_3 and DMSO-d_6) δ 8.68 (s, 1H), 8.10 (d, J = 6.1 Hz, 2H), 7.91 (s, 1H), 7.86 (m, 2H), 7.82 – 7.74 (m, 3H), 7.72 (d, J = 7.4 Hz, 2H), 7.68 (m, 3H), 7.30 (d, J = 8.4 Hz, 2H), 3.64 – 3.45 (m, 2H), 3.44 – 3.30 (m, 2H), 2.28 (s, 3H); ^{13}C NMR (151 MHz, Acetonitrile-d_3 and DMSO-d_6) δ 175.6, 169.9, 163.7, 156.2, 153.1, 142.7, 137.7, 134.1, 132.8, 132.3, 131.8, 131.0, 130.5, 130.3, 130.0, 129.7, 129.3, 129.1, 127.5, 123.5, 33.6, 30.7, 21.3.

3c: ^{1}H NMR (400 MHz, Acetonitrile-d_3 and DMSO-d_6) δ 8.56 (s, 1H), 8.06 (d, J = 7.3 Hz, 2H), 7.91 (s, 1H), 7.86 – 7.79 (m, 2H), 7.75 (d, J = 7.8 Hz, 3H), 7.71 (d, J = 7.6 Hz, 2H), 7.70 – 7.62 (m, 3H), 7.12 (d, J = 8.5 Hz, 2H), 3.88 (s, 3H), 3.56 – 3.47 (m, 2H), 3.39 – 3.31 (m, 2H); ^{13}C NMR (126 MHz, Acetonitrile-d_3 and DMSO-d_6) δ 172.4, 162.9, 162.1, 159.4, 150.5, 140.6, 138.0, 136.6, 135.2, 134.1, 133.9, 133.6, 132.2, 131.0, 130.1, 129.9, 129.1, 128.9, 115.9, 56.4, 33.4, 30.8.

3d: ^{1}H NMR (600 MHz, Acetonitrile-d_3 and DMSO-d_6) δ 8.61 (s, 1H), 8.08 (d, J = 7.5 Hz, 2H), 7.88 (s, 1H), 7.85 – 7.80 (m, 2H), 7.75 (d, J = 7.3 Hz, 1H), 7.71 (d, J = 7.8 Hz, 2H), 7.70 – 7.64 (m, 5H), 7.39 (d, J = 8.5 Hz, 2H), 3.58 – 3.44 (m, 2H), 3.38 – 3.31 (m, 2H), 2.54 (s, 3H); ^{13}C NMR (151 MHz, Acetonitrile-d_3 and DMSO-d_6) δ 171.4, 162.4, 159.3, 150.8, 144.4, 141.9, 137.7, 135.6, 135.0, 133.9, 132.1, 131.9, 130.8, 130.5, 130.4, 129.9, 129.0, 126.3, 125.8, 33.4, 30.7, 14.6.

3e: ^{1}H NMR (600 MHz, Acetonitrile-d_3 and DMSO-d_6) δ 8.16 (s, 1H), 8.00 – 7.93 (m, 2H), 7.84 (s, 1H), 7.76 – 7.71 (m, 2H), 7.69 – 7.66 (m, 3H), 7.64 (d, J = 7.4 Hz, 2H), 7.63 – 7.59 (m, 3H), 6.87 (d, J = 9.2 Hz, 2H), 3.44 – 3.34 (m, 2H), 3.28 – 3.22 (m, 2H), 3.11 (s, 6H); ^{13}C NMR (151 MHz, Acetonitrile-d_3 and DMSO-d_6) δ 171.7, 155.9, 154.7, 153.8, 148.4, 139.6, 138.1, 137.0, 135.4, 135.2, 132.9, 131.3, 131.0, 130.5, 129.7, 129.3, 128.3, 124.2, 113.4, 39.6, 32.4, 30.8.

3f: ^{1}H NMR (400 MHz, Acetonitrile-d_3 and DMSO-d_6) δ 8.29 (s, 1H), 8.21 (d, J = 7.1 Hz, 1H), 8.13 (s, 1H), 7.97 (d, J = 7.1 Hz, 2H), 7.76 (m, 2H), 7.73 – 7.68 (m, 2H), 7.69 – 7.62 (m, 4H), 7.58 (d, J = 8.7 Hz, 2H), 7.52 (d, J = 4.0 Hz, 1H), 6.66 (d, J = 8.7 Hz, 2H), 3.48 – 3.40 (m, 2H), 3.19 – 3.12 (m, 2H), 2.92 (s, 6H); ^{13}C NMR (126 MHz, Acetonitrile-d_3 and DMSO-d_6) δ 170.1, 169.5, 158.4, 156.7, 156.3, 152.0, 150.1, 139.3, 138.3, 137.9, 135.0, 134.4, 133.4, 131.8, 130.9, 130.7, 129.9, 129.6, 128.6, 128.0, 124.1, 120.8, 112.8, 32.7, 32.2, 30.8.

3g: ^{1}H NMR (400 MHz, Acetonitrile-d_3 and DMSO-d_6) δ 8.62 (s, 1H), 8.13 (d, J = 8.4 Hz, 2H), 7.98 – 7.78 (m, 3H), 7.73 (d, J = 7.3 Hz, 2H), 7.69 – 7.61 (m, 3H), 7.53 (d, J = 7.4 Hz, 3H), 7.30 (d, J = 8.5 Hz, 2H), 4.95 (d, J = 2.4 Hz, 2H), 3.52 – 3.43 (m, 2H), 3.41 – 3.33 (m, 2H), 3.31 (t, J = 2.4 Hz, 1H); ^{13}C NMR (126 MHz, Acetonitrile-d_3 and DMSO-d_6) δ 169.5, 163.9, 162.7, 160.1, 150.3, 143.0, 137.8, 136.0, 134.9, 132.9, 132.2, 131.3, 131.2, 131.1, 130.5, 130.0, 129.8, 128.1, 117.4, 78.8, 78.5, 57.0, 33.5, 30.7; MS (ESI) Calcd for: $C_{30}H_{23}OS^+$ ($[M-BF_4]^+$): 431.15, found:431.21.

3h: ^{1}H NMR (600 MHz, Acetonitrile-d_3 and DMSO-d_6) δ 8.57 (s, 1H), 8.11 (d, J = 8.6 Hz, 2H), 7.84 (s, 1H), 7.83 – 7.79 (m, 2H), 7.77 (d, J = 8.3 Hz, 2H), 7.69 – 7.60 (m, 3H), 7.30 (d, J = 8.9 Hz, 2H), 7.28 (d, J = 8.6 Hz, 2H), 4.94 (d, J = 2.4 Hz, 2H), 3.51 – 3.44 (m, 2H), 3.39 – 3.33 (m, 2H), 3.22 (t, J = 2.4 Hz, 1H), 2.28 (s, 3H); ^{13}C NMR (151 MHz, Acetonitrile-d_3 and DMSO-d_6) δ 170.0, 162.7, 152.9, 142.9, 137.8, 134.2, 133.6, 132.6, 132.2, 131.7, 131.1, 130.8, 130.5, 130.0, 129.9, 129.8, 128.1, 123.4, 117.3, 78.8, 78.5, 56.9, 36.0, 33.4, 21.2; HRMS (ESI) Calcd for: $C_{31}H_{25}O_3S^+$ ($[M-BF_4]^+$): 489.1519, found: 489.1509.
3i: ^{1}H NMR (500 MHz, Acetonitrile-d_3 and DMSO-d_6) δ 8.50 (s, 1H), 8.09 (d, J = 8.9 Hz, 2H), 7.84 (s, 1H), 7.83 – 7.77 (m, 2H), 7.72 (d, J = 8.8 Hz, 2H), 7.69 – 7.61 (m, 3H), 7.29 (d, J = 9.0 Hz, 2H), 7.10 (d, J = 8.9 Hz, 2H), 4.94 (d, J = 2.4 Hz, 2H), 3.87 (s, 3H), 3.46 (m, 2H), 3.36 – 3.28 (m, 2H), 3.25 (t, J = 2.4 Hz, 1H); ^{13}C NMR (126 MHz, Acetonitrile-d_3 and DMSO-d_6) δ 170.5, 162.6, 162.3, 159.2, 149.5, 140.5, 138.0, 135.7, 133.8, 132.3, 132.1, 131.0, 130.5, 130.0, 129.8, 128.9, 128.2, 117.3, 115.7, 78.9, 78.6, 57.0, 56.3, 33.3, 30.7; HRMS (ESI) Calcd for: $C_{31}H_{25}O_2S^+$ ($[M-BF_4]^+$): 461.1570, found: 461.1555.
3j: ^{1}H NMR (500 MHz, Acetonitrile-d_3 and DMSO-d_6) δ 8.52 (s, 1H), 8.09 (d, J = 8.8 Hz, 2H), 7.86 – 7.75 (m, 3H), 7.66 (m, 5H), 7.39 (d, J = 8.6 Hz, 2H), 7.29 (d, J = 9.0 Hz, 2H), 4.93 (d, J = 2.4 Hz, 2H), 3.52 – 3.44 (m, 2H), 3.33 (m, 2H), 3.22 (t, J = 2.4 Hz, 1H), 2.54 (s, 3H); ^{13}C NMR (126 MHz, Acetonitrile-d_3 and DMSO-d_6) δ 169.7, 162.4, 159.3, 149.8, 144.0, 141.9, 137.7, 134.7, 134.0, 132.2, 131.8, 130.9, 130.4, 129.9, 129.7, 128.0, 126.3, 125.8, 117.2, 78.7, 78.5, 56.8, 33.3, 30.6, 14.6; HRMS (ESI) Calcd for: $C_{31}H_{25}OS_2^+$ ($[M-BF_4]^+$): 477.1341, found: 477.1330.
3k: ^{1}H NMR (500 MHz, Acetonitrile-d_3) δ 8.05 (s, 1H), 7.90 (d, J = 8.8 Hz, 2H), 7.72 (s, 1H), 7.71 – 7.68 (m, 2H), 7.66 – 7.61 (m, 5H), 7.24 (d, J = 8.8 Hz, 2H), 6.84 (d, J = 9.0 Hz, 2H), 4.89 (d, J = 2.4 Hz, 2H), 3.39 – 3.33 (m, 2H), 3.28 – 3.22 (m, 2H), 3.11 (s, 6H), 2.93 (t, J = 2.4 Hz, 1H); ^{13}C NMR (126 MHz, Acetonitrile-d_3) δ 170.4, 161.1, 156.0, 154.8, 153.0, 147.2, 138.2, 137.6, 136.4, 134.5, 130.8, 129.4, 129.3, 129.2, 128.6, 127.7, 123.5, 116.2, 112.6, 77.9, 76.7, 56.1, 39.5, 31.9, 30.2; HRMS (ESI) Calcd for: $C_{32}H_{28}NOS^+$ ($[M-BF_4]^+$): 474.1886, found:474.1895.
H4: ^{1}H NMR (500 MHz, Acetonitrile-d_3 and DMSO-d_6) δ 8.19 (s, 1H), 7.96 (d, J = 8.7 Hz, 2H), 7.72 (s, 2H), 7.66 – 7.62 (m, 3H), 7.58 (s, 1H), 7.50 (d, J = 8.9 Hz, 2H), 7.45 (d, J = 4.1 Hz, 1H), 7.41 (d, J = 8.5 Hz, 1H), 7.23 (d, J = 8.9 Hz, 2H), 6.59 (d, J = 9.0 Hz, 2H), 4.92 (d, J = 2.4 Hz, 2H), 3.43 – 3.32 (m, 2H), 3.29 (t, J = 2.4 Hz, 1H), 3.10 – 3.02 (m, 2H), 2.87 (s, 6H); ^{13}C NMR (126 MHz, Acetonitrile-d_3 and DMSO-d_6) δ 169.7, 162.2, 158.8, 156.0, 152.1, 151.9, 149.1, 144.7, 138.8, 138.0, 134.1, 131.8, 130.4, 129.9, 129.7, 128.0, 127.9, 124.2, 123.8, 121.5, 120.9, 117.1, 112.9, 79.0, 78.7, 56.9, 32.8, 30.4, 21.3; HRMS (ESI) Calcd for: $C_{36}H_{30}NOS_2^+$ ($[M-BF_4]^+$): 556.18, found: 556.42.
MALDI-TOF-MS. Expected M.W.2093.46 ($[M-BF_4]^+$), Measured M.W. 2093.63.

*Synthesis of **3j-PEG, 3k-PEG, H4-PEG** were similar to the **H4-PEG-PT.** **3j-PEG:*** Expected M.W.1297.06 ($[M-BF_4]^+$, Measured M.W. 1297.03; ***3k-PEG:*** Expected M.W.1294.12 ($[M-BF_4]^+$, Measured M.W. 1294.31; ***H4-PEG:*** Expected M.W.1376.11 ($[M-BF_4]^+$, Measured M.W. 1376.37.

The cellular uptake of 143B and HepG2 cells treated with H4-PEG-PT

Human bone osteosarcoma 143B cells and human hepatocellular carcinoma HepG2 cells were dispersed in

20 mm confocal dishes, and cultured for 12 h at a density of 1×10^5 cells/well. Then, 200 μL of **H4-PEG-PT** (64 μM) was introduced into the confocal dishes. After 7 h incubation, 143B and HepG2 cells were washed twice with PBS, stained with Hoechst staining kit in PBS for 20 min. The tumor cell samples were immediately examined by an inverted Zeiss Axiovert 200 fluorescence microscopy at a 20× objective, using a high-pressure mercury lamp as an excitation source.

Detection of ATP level

143B cells (6×10^3) were incubated in 96 wells plate at 37 °C for 12 h. Then, cells were treated with 64 μM **H4-PEG-PT** or 40 μM CCCP for 6 h and irradiated by an 808 nm laser for 3 min each well. After irradiation, ATP level was immediately determined by an ATP determination kit (cat. no. A22066) from Thermo Fisher Scientific with 1% Triton X 100 water solution for cell permeabilization. After 10 min incubation, the luminescence intensity was then detected using a microplate reader (TECAN, Infinite M200 Pro).

Detection of mitochondrial membrane potential by JC-1 assay

143B cells (6×10^3) were incubated in 96 wells plate for 12 h. Then, cells were treated with **H4-PEG-PT (**64 μM), or CCCP (40 μM) for 6 h and irradiated by an 808 nm laser for 3 min. After irradiation, JC-1 (1 μM) was immediately added to each well for 30 min incubation at 37 °C. Fluorescence intensity was then detected using a microplate reader (TECAN, Infinite M200 Pro) with excitation at 485 nm (green) or 535 nm (red) and emissions at 535 nm (green) or 595 nm (red). The ratio of red fluorescence intensity (J-aggregates) to green fluorescence intensity (monomers) was used to quantify the mitochondrial membrane potential change of **H4-PEG-PT** with laser irradiation, the ratio of the control was 100%.

Detection of mitochondrial permeability transition

143B cells (6×10^3) were incubated in 96 wells plate for 12 h. Then, 100 μL of **H4-PEG-PT** (64 μM) in fresh DMEM medium was introduced into the 96 wells plate. After an incubation time of 6 h, cells were irradiated by an 808 nm laser for 3 min. After that, cells were loaded with 1 μM of calcein-acetoxymethyl ester (AM) in Hank's buffer at 37 °C for 10 min and treated with 2 mM cobalt chloride or 2 mM cobalt chloride plus 10 mM hydrogen peroxide Hank's buffer solution for 10 min incubation at 37 °C. The fluorescence intensity of cell samples was measured using a microplate reader (TECAN, Infinite M200 Pro) with 488 nm excitation and 535 nm emission filters.

Dichloro-dihydro-fluorescein diacetate (DCFH-DA) assay

143 B cells (1×10^4 cells/well) were seeded into a 96-well plate and incubated with 30 μM **H4-PEG-PT** for 6 h and 10 μM DCFH-DA (Ex/Em = 504/529 nm) for 30 min. The cells were then exposed to laser irradiation (2 W/cm^2, 808 nm) for 3 min prior imaging. The tumor cell samples were immediately examined by an inverted Zeiss Axiovert 200 fluorescence microscopy at a 10× objective, using a high-pressure mercury lamp as excitation source.

Caspase-3/8/9 activities measured by caspase multiplex activity assay kit

143 B cells were seeded on the same day at 1 x 10^5 cells/well in a clear bottom 96-well plate. DOX•HCl treatment is the positive control. Briefly, 143B cells (6×10^3) were seeded in a clear bottom 96 wells plate for 12 h. Then, 100 μL of **H4-PEG-PT** (64 μM) in fresh DMEM medium was introduced into the 96 wells plate. After an incubation time of 6 h, cells were irradiated by an 808 nm laser for 3 min. After that, cells incubated at 37 °C for 30 min, then Triple-caspase assay loading solution (100 μL/well for Caspase 3, 8 and 9 together) was added to cells, followed by an incubation at RT for 30 min. The fluorescence intensity was measured at the indicated wavelength using a microplate reader (TECAN, Infinite M200 Pro).

Apoptosis or necrosis of 143B cells by Annexin V-FITC/PI and Hochest/PI staining

143B cells were seeded in 96-well plates at a density of 1.2×10^4 cell/well. After treatment, the cells were harvested and re-suspended in binding buffer. Cells were stained with Annexin V-FITC and PI according to the manufacturer's instructions, followed by laser irradiation to induce cell death. Besides, we also used PI and Hochest dual staining to evaluate the cellular apoptosis and necrosis. Normal cell nuclei are usually blue, while apoptotic cell nuclei are densely stained with bright blue.

Ethics statement

All animal studies were performed in accordance with the Guidelines for the Care and Use of Laboratory Animals of the Chinese Animal Welfare Committee and approved by The Institutional Animal Care and Use Committee (IACUC), Wuhan University Center for Animal Experiment, Wuhan, China.

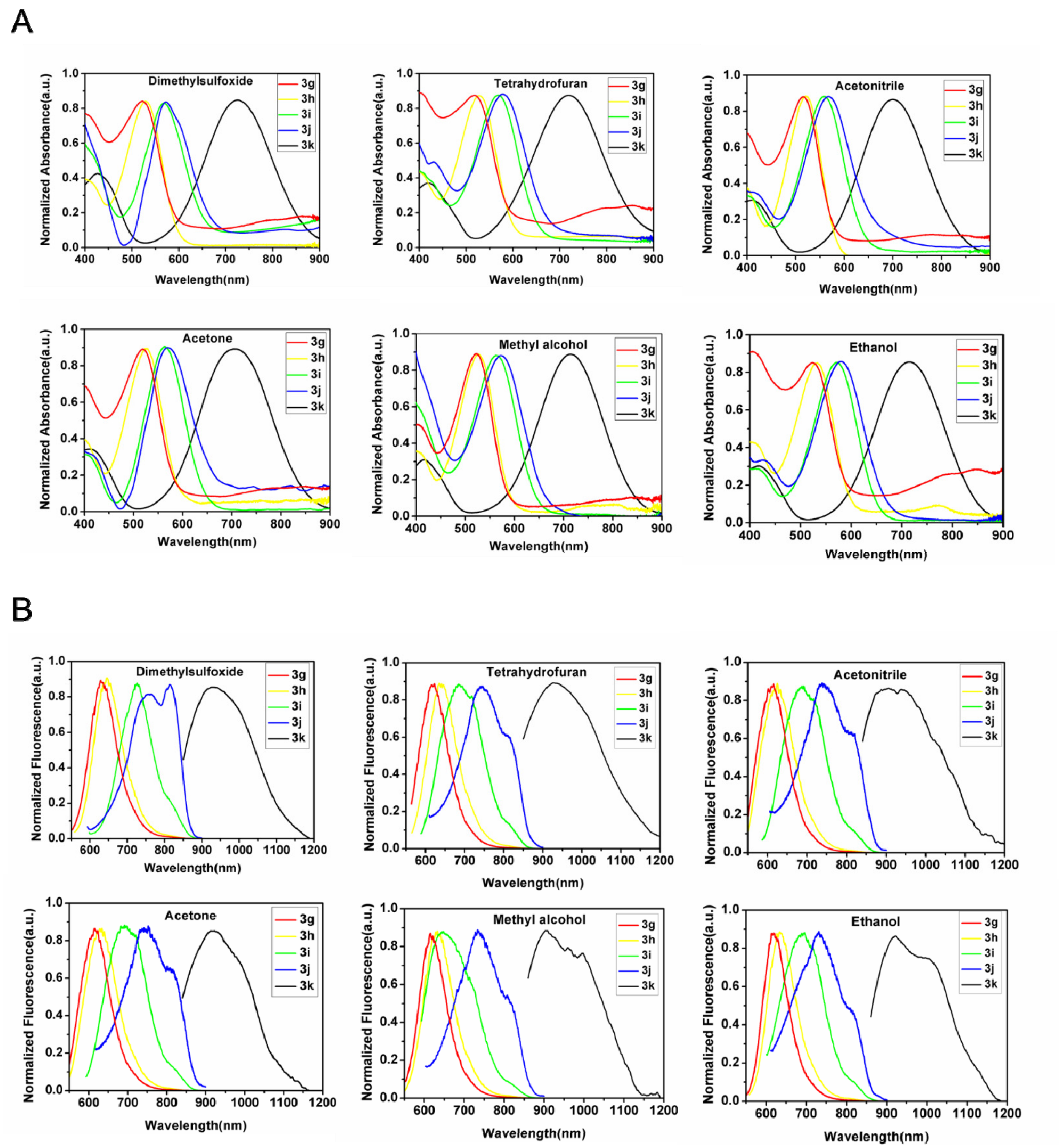

Supplementary Fig. 1. (A) Absorption wavelength of **3g-3k** in different solvents. (B) Emission wavelength of **3g-3k** in different solvents.

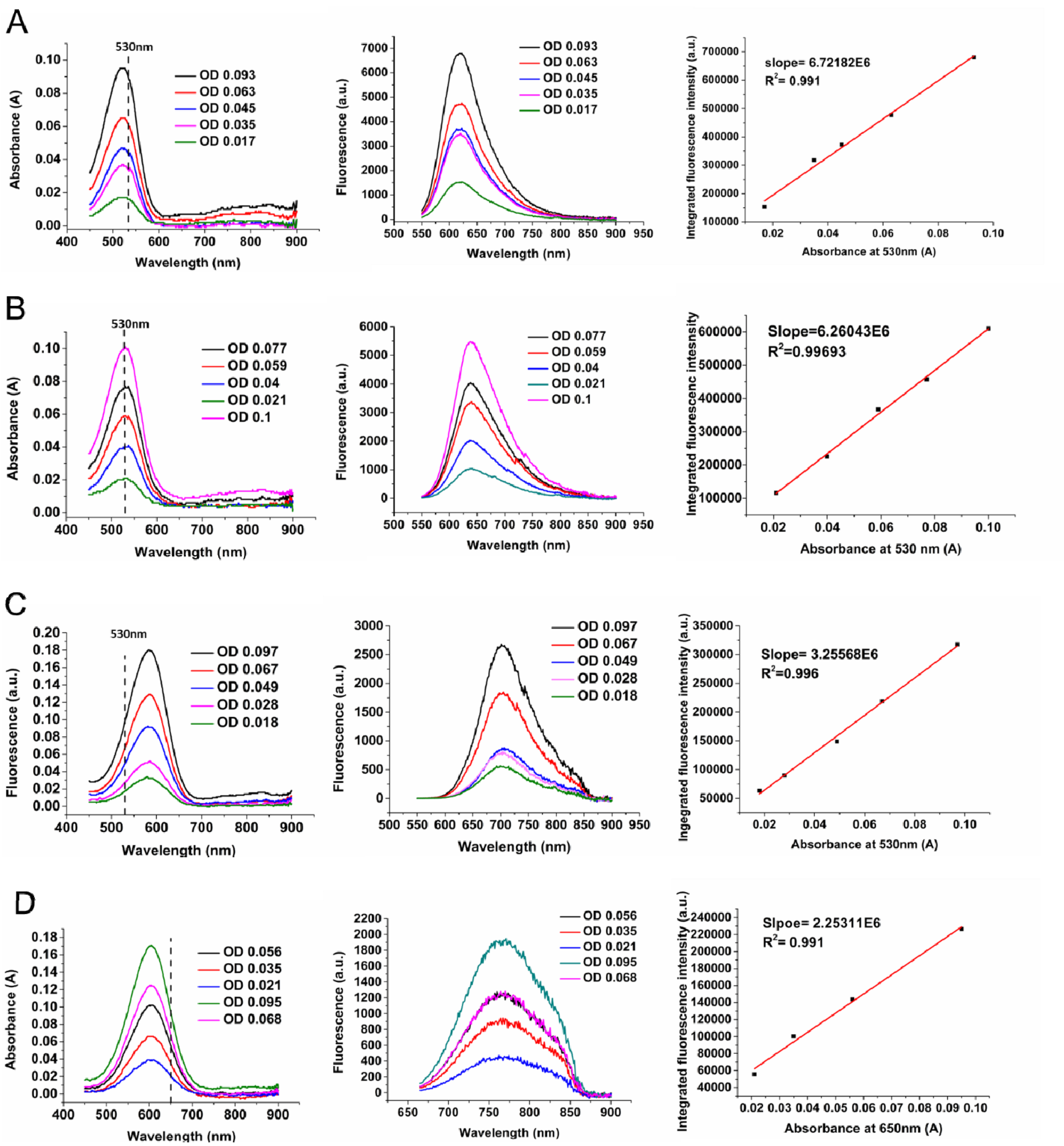
A
530nm
Absorbance (A)
Wavelength (nm)
OD 0.093
OD 0.063
OD 0.045
OD 0.035
OD 0.017
Fluorescence (a.u.)
Wavelength (nm)
Integrated fluorescence intensity (a.u.)
slope= 6.72182E6
R^2= 0.991
Absorbance at 530nm (A)
B
530nm
Absorbance (A)
Wavelength (nm)
OD 0.077
OD 0.059
OD 0.04
OD 0.021
OD 0.1
Fluorescence (a.u.)
Wavelength (nm)
Integrated fluorescenc intesnsity
Slope=6.26043E6
R^2=0.99693
Absorbance at 530 nm (A)
C
530nm
Fluorescence (a.u.)
Wavelength (nm)
OD 0.097
OD 0.067
OD 0.049
OD 0.028
OD 0.018
Fluorescence (a.u.)
Wavelength (nm)
Ingegrated fluorescence intensity (a.u.)
Slope= 3.25568E6
R^2=0.996
Absorbance at 530nm (A)
D
Absorbance (A)
Wavelength (nm)
OD 0.056
OD 0.035
OD 0.021
OD 0.095
OD 0.068
Fluorescence (a.u.)
Wavelength (nm)
Integrated fluorescence intensity (a.u.)
Slpoe= 2.25311E6
R^2= 0.991
Absorbance at 650nm (A)

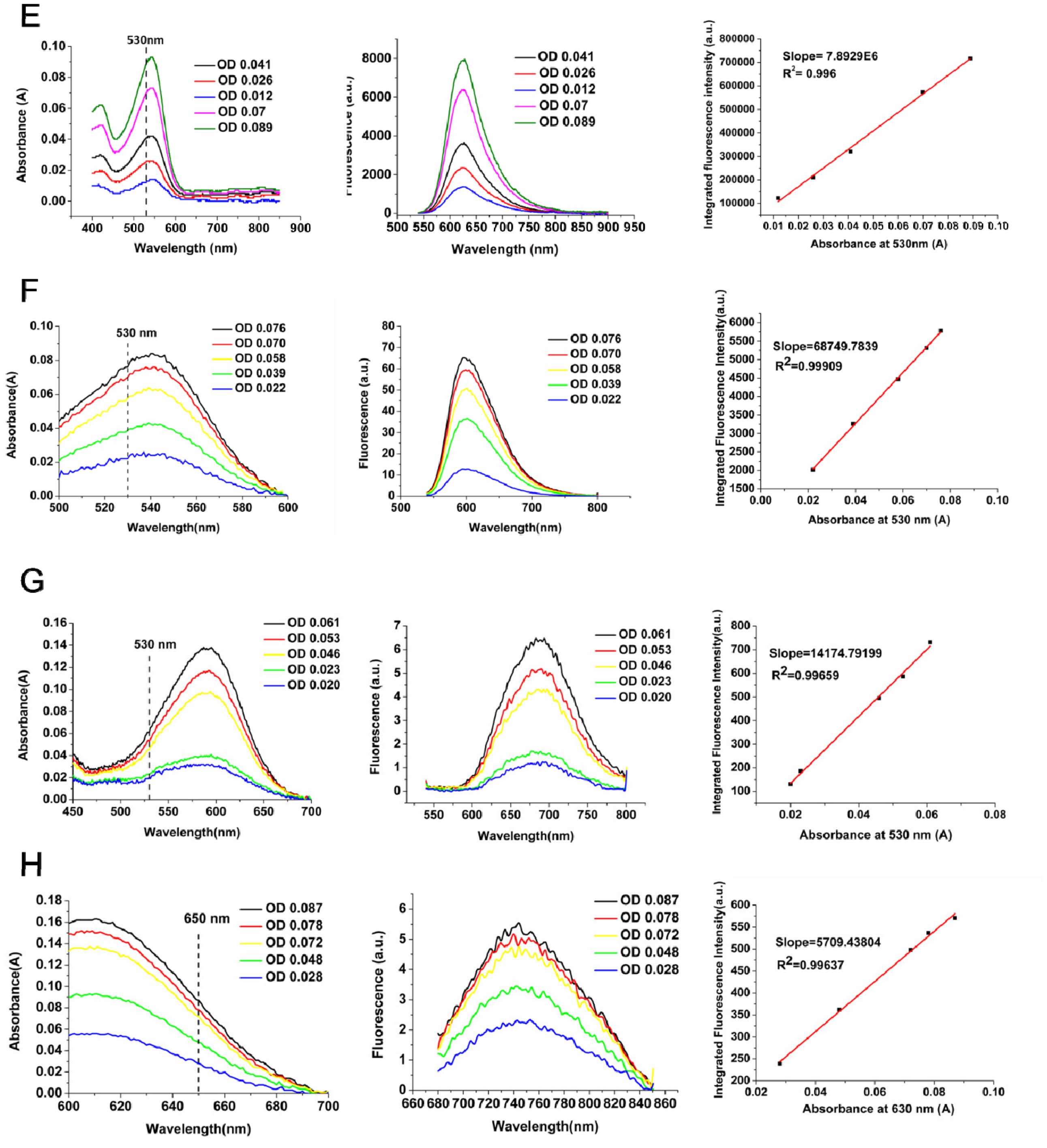

Supplementary Fig. 2. Quantum yield measurements of **3a** (A)**, 3b** (B), **3c** (C), **3d** (D),**3g** (E), **3h** (F),**3i** (G),**3j** (H) in dichloromethane.

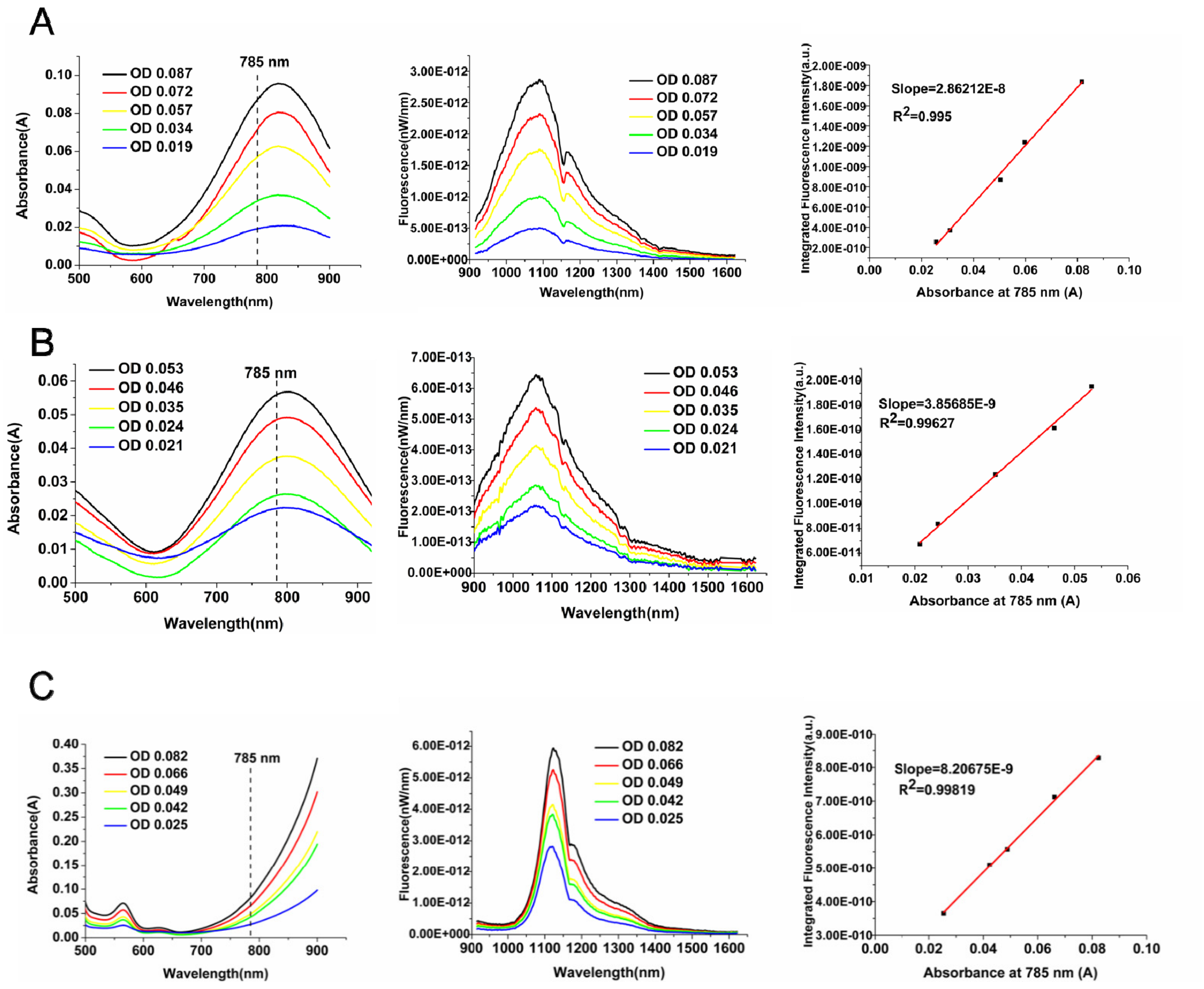

Supplementary Fig. 3. Quantum yield measurements of **H4** in dichloromethane (A) and **H4-PEG-PT** in water (B). A reference IR-26 (0.5%) was chosen (C).

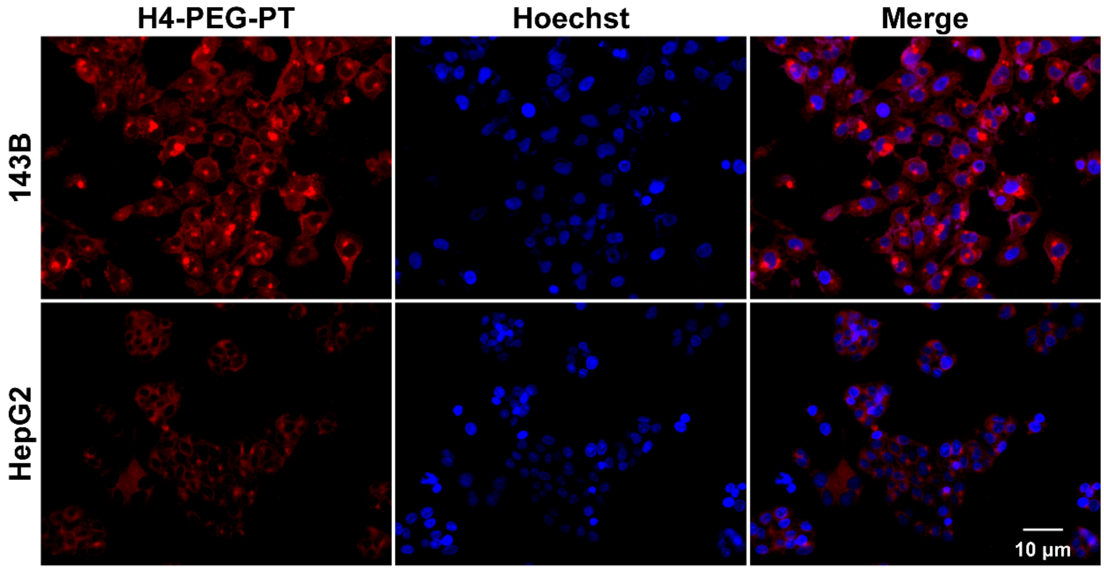

Supplementary Fig. 4. The *in vitro* cellular uptake of **H4-PEG-PT** was investigated against 143B cells. Scale bar = 10 μm. The results are representative of three independent experiments.

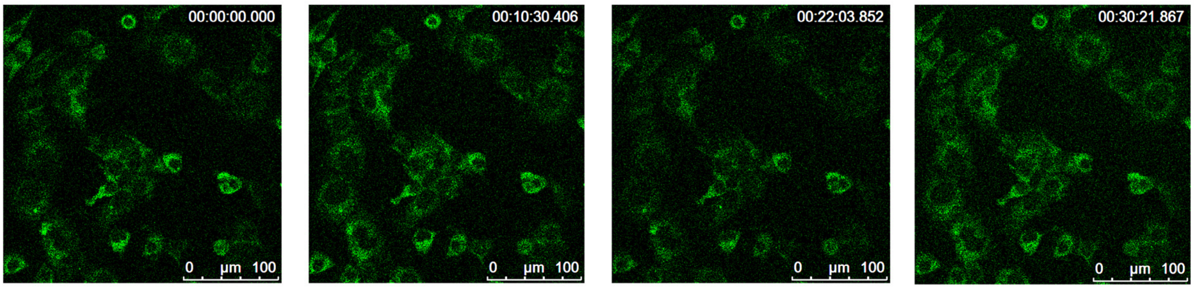

Supplementary Fig. 5. Fluorescent images of **3j-PEG** in 143B cells with ethanol fixation at 0, 10, 22 and 30 min. The results are representative of three independent experiments.

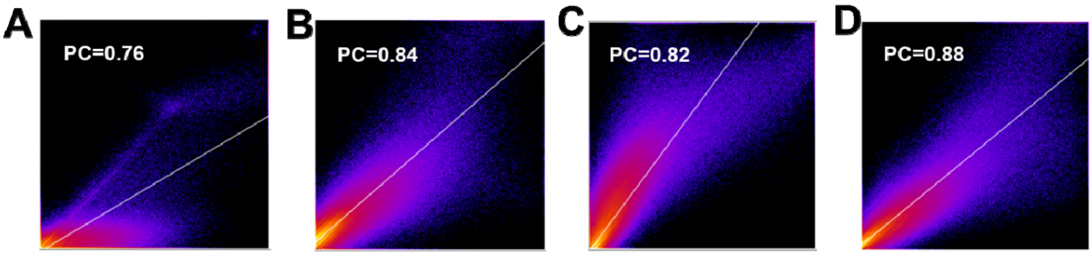

Supplementary Fig. 6. Colocalization scatterplots of **3j-PEG** and mito-tracker red, PC = 0.76. (B) Colocalization scatterplots of **3k-PEG** and mito-tracker green, PC = 0.84. (C) Colocalization scatterplots of **H4-PEG** and mito-tracker green, PC = 0.82. (D) Colocalization scatterplots of **H4-PEG-PT** and mito-tracker green. PC = 0.88

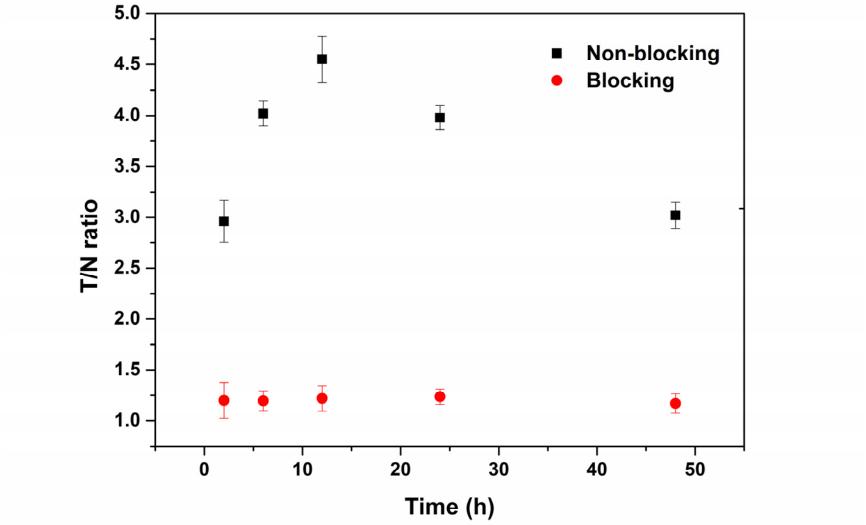

Supplementary Fig. 7. The NIR-II imaging, represented as (T/N) ratio for the **H4-PEG-PT** probe (black bar) and blocking group (red bar) at 2, 6, 12, 24, 48 h (under 808 nm excitation laser, 1000 LP and 250 ms). (n = 3 biologically independent mice). Data are presented as mean values ± SD.

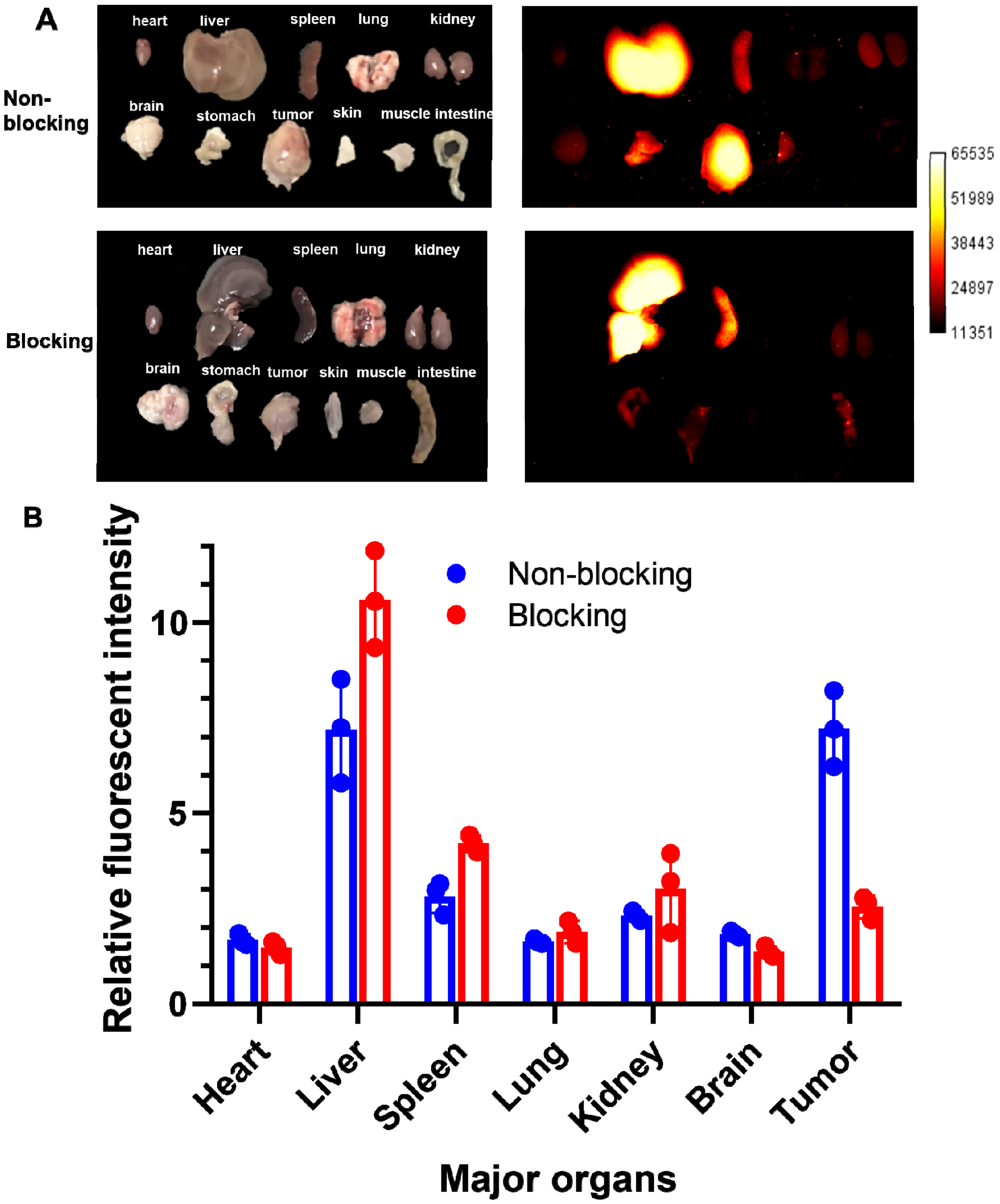

Supplementary Fig. 8. The biodistribution of **H4-PEG-PT** in orthotopic tumor mice at 48 h under an 808 nm excitation (1000LP, 3.5W and 200 ms) and the relative fluorescence intensity analysis of different organs (n = 3 biologically independent mice). Data are presented as mean values ± SD.

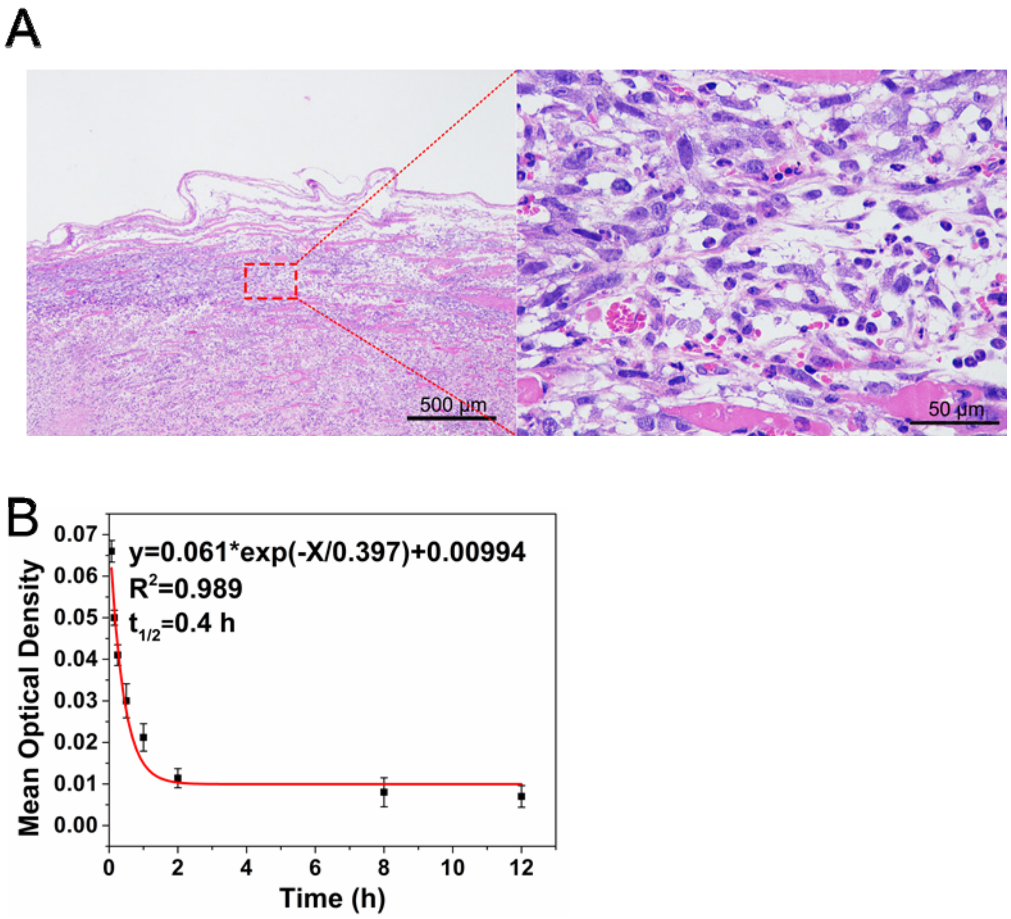

Supplementary Fig. 9. (A) H&E staining of 143B tumor tissues and (B) Relative fluorescence fluorescent intensity of **H4-PEG-PT** at different time points in the blood (n = 3 biologically independent samples). Data are presented as mean values ± SD.

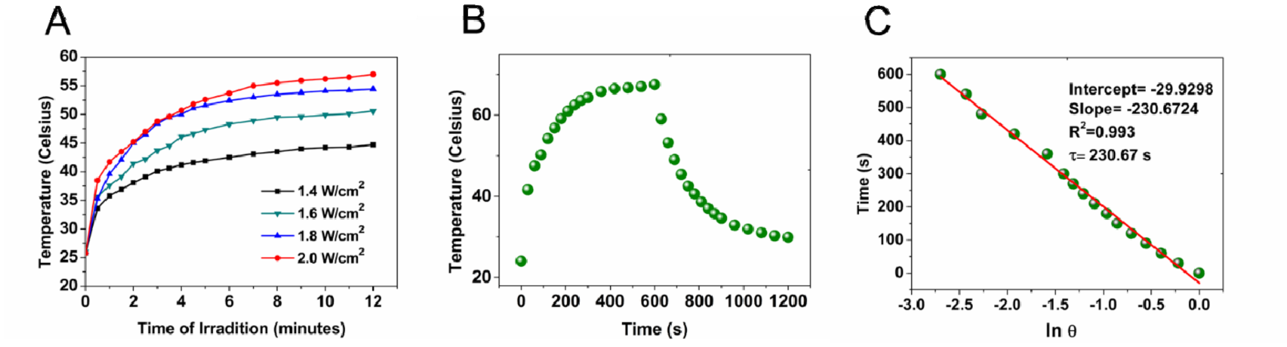

Supplementary Fig. 10. (A) Photo-thermal heating curves of **H4-PEG-PT** with 64 μM at different laser power densities. (B) The cooling curve of **H4-PEG-PT** (75 μM) after the irradiation of 808 nm laser (2.0 W/cm^2) and (C) its corresponding time-Inθ linear curve.

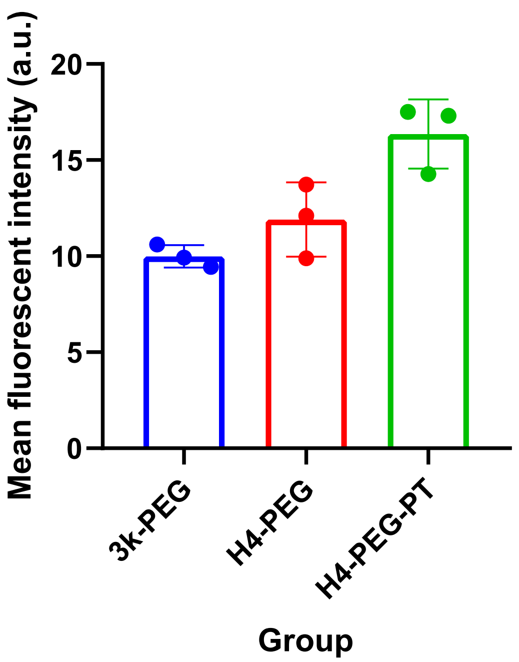

Supplementary Fig. 11. Quantitative analysis of Cyto c was measured in different groups by the photothermal therapy (n = 3 biologically independent samples). Data are presented as mean values ± SD.

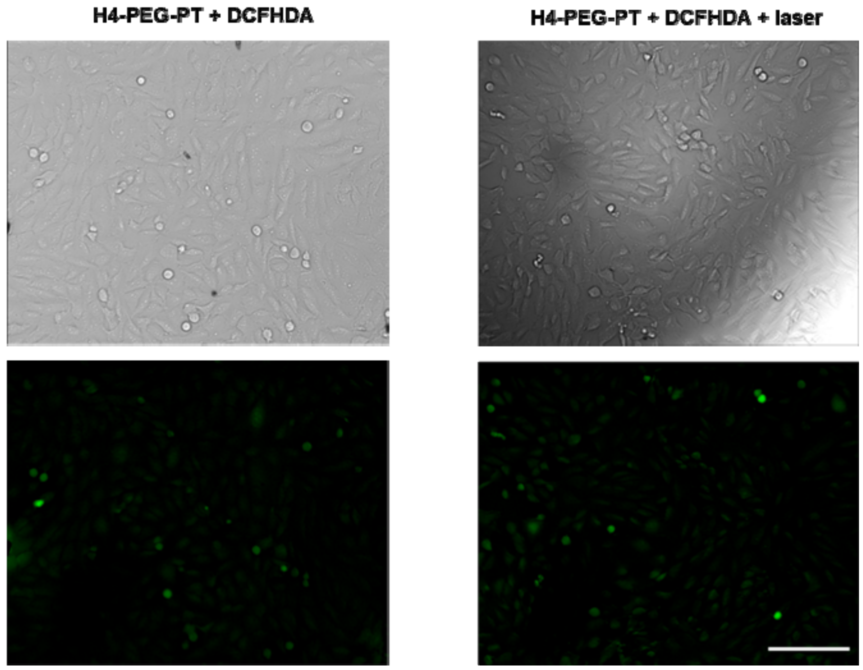

Supplementary Fig. 12. Intracellular ROS generation using DCFH-DA assay in 143B cells incubated with **H4-PEG-PT,** Scale bar: 100 µm. The results are representative of three independent experiments.

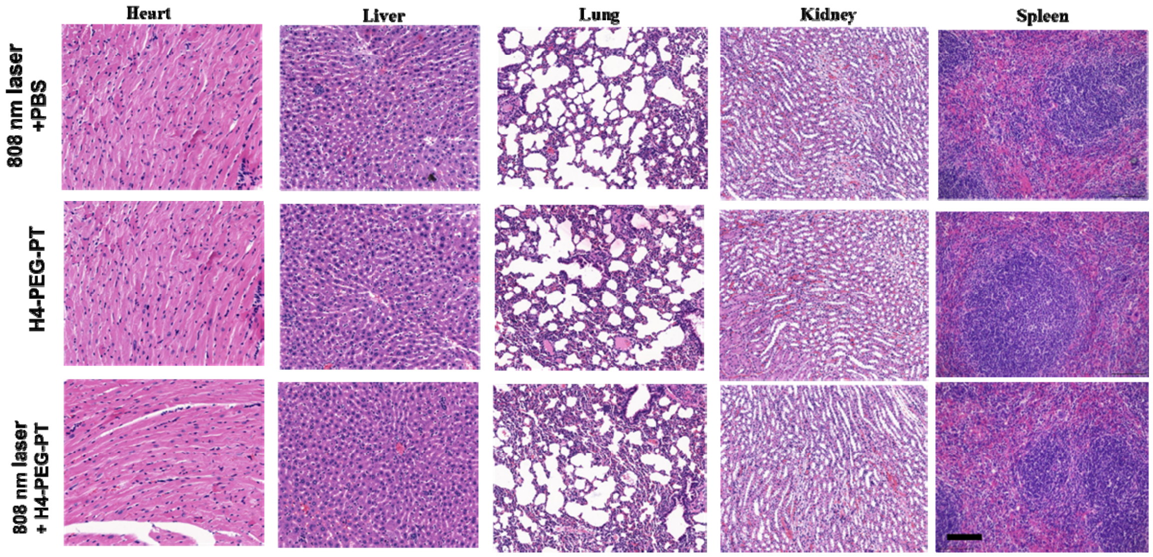

Supplementary Fig. 13. Major organs (heart, liver, spleen, lung, and kidney) from the tumor bearing mice in different treatment groups with H&E staining (scale bar: 100 μm) in photothermal experiments. The results are representative of three independent experiments.

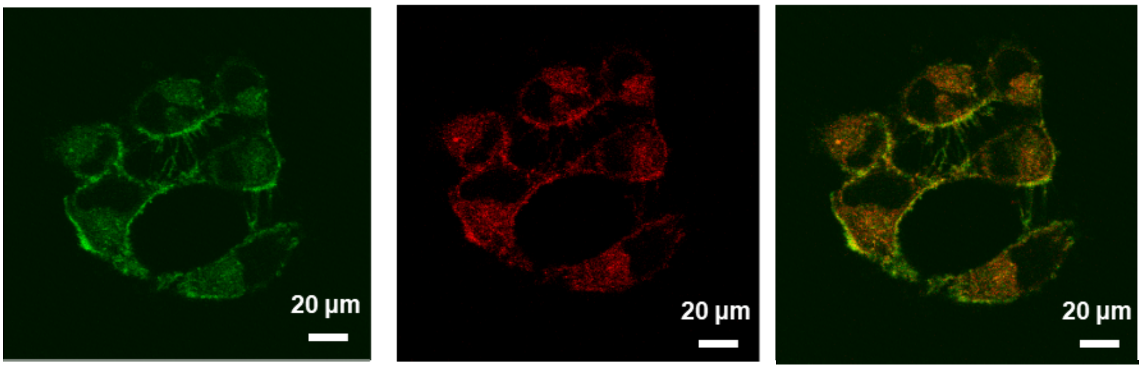

Supplementary Fig. 14. Confocal fluorescence microscopy studies of 143B cells incubated with Mito-tracker green (left); with **H4-PEG-PT** for 6 h (middle) and merge images of **H4-PEG-PT** (1 nM) and Mito-tracker green (right). λ_{em}: 500-530 nm, λ_{ex}: 488 nm (Mito-tracker green); λ_{em}: 580 nm-650 nm, λ_{ex}: 808 nm (**H4-PEG-PT**). The results are representative of three independent experiments.

Supplementary Table 1 Comparison of HOMO and LUMO orbital surfaces of **3a-3k** and **H4** using DFT B3LYP/6-31G(d) scrf = (cpcm, solvent = dichloromethane) method

Compound	HOMO	Energy (eV)	LUMO	Energy (eV)	E_{gap} (eV)	Compound	HOMO	Energy (eV)	LOMO	Energy (eV)	E_{gap} (eV)
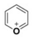	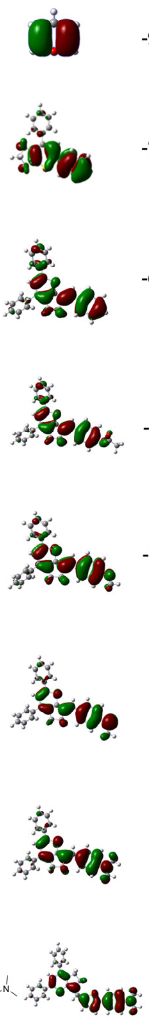	-9.62	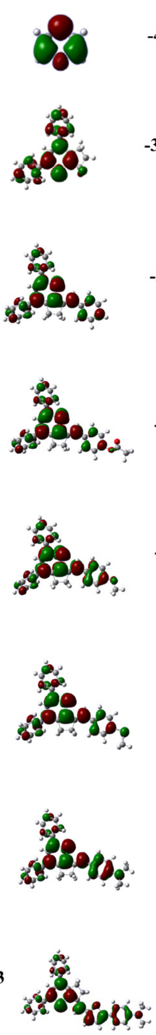	-4.03	5.59	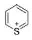	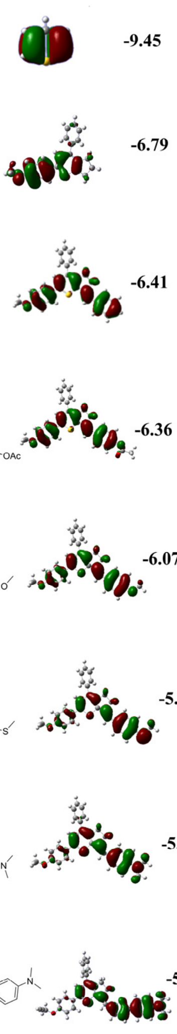	-9.45	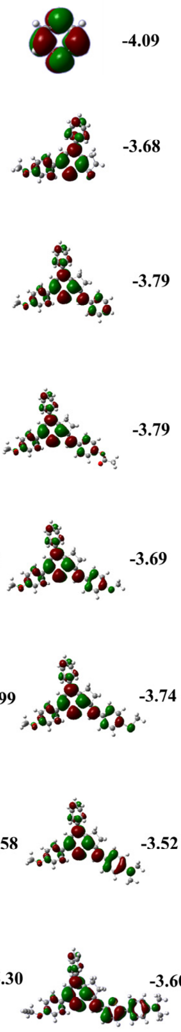	-4.09	5.37
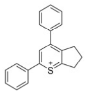		-7.39		-3.75	3.64	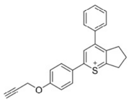		-6.79		-3.68	3.11
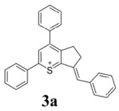 **3a**		-6.53		-3.86	2.67	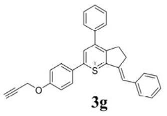 **3g**		-6.41		-3.79	2.62
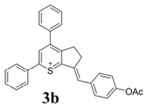 **3b**		-6.47		-3.86	2.61	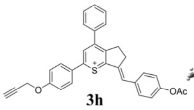 **3h**		-6.36		-3.79	2.57
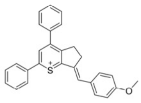 **3c**		-6.14		-3.75	2.39	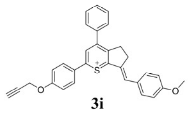 **3i**		-6.07		-3.69	2.38
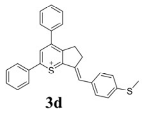 **3d**		-6.04		-3.80	2.24	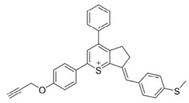 **3j**		-5.99		-3.74	2.25
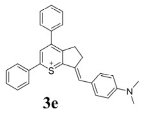 **3e**		-5.63		-3.56	2.07	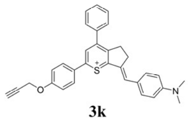 **3k**		-5.58		-3.52	2.06
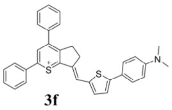 **3f**		-5.33		-3.63	1.7	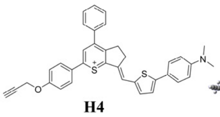 **H4**		-5.30		-3.60	1.7

Supplementary Table 2 Optical properties of **3a-3k** and **H4** in dichloromethane

Compound	$\lambda_{abs,max}$ (nm)	$\lambda_{em,max}$ (nm)	ε (L × mol^{-1} × cm^{-1})	QY (%) (In dichloromethane)
**3a**	520	618	7906.2	29.6
**3b**	528	645	7407.6	27.5
**3c**	577	696	14645	14.3
**3d**	599	757	15207	21.5
**3e**	741	917	30744	*N.A.
**3f**	826	1100	35082	1.78
**3g**	540	615	9867.9	30.90
**3h**	545	630	31860	32.99
**3i**	591	692	25249	10.92
**3j**	600	745	29283	29.01
**3k**	743	918	116894	*N.A.
**H4**	826	1100	40432	2.01

[a] Rhodamine 6G was chosen as a reference when measuring fluorescence quantum yield of **3a**, **3b**, **3c**, **3g**,**3h**,**3i**. [b] ICG was chosen as a reference when measuring fluorescence quantum yield of **3d** and **3j**. [c] IR-26 was chosen as a reference when measuring fluorescence quantum yield of **3f** and **H4**. *Due to unavailable references for absolute quantum yield measurement, we have not measured the quantum yields of **3e** and **3k** (λ em,max (nm) = ~910 nm).

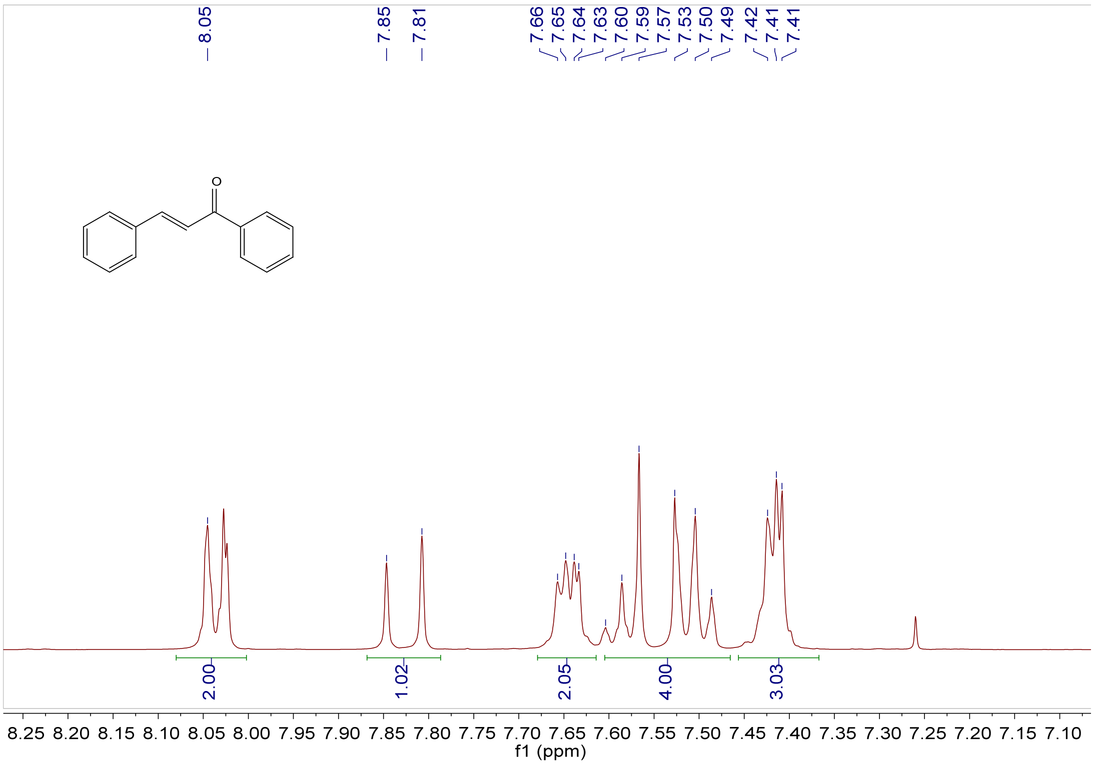

Supplementary Fig. 15 ^{1}H NMR spectra of compound **5a** in $CDCl_3$

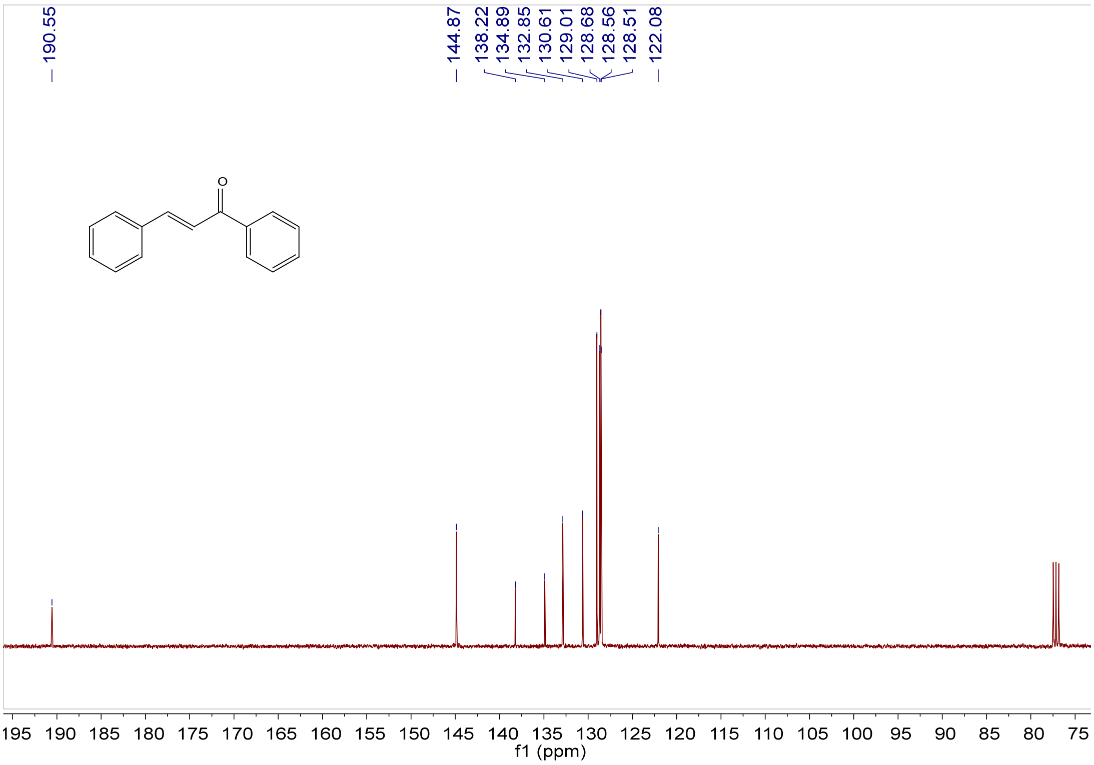

Supplementary Fig. 16 ^{13}C NMR spectra of compound **5a** in $CDCl_3$

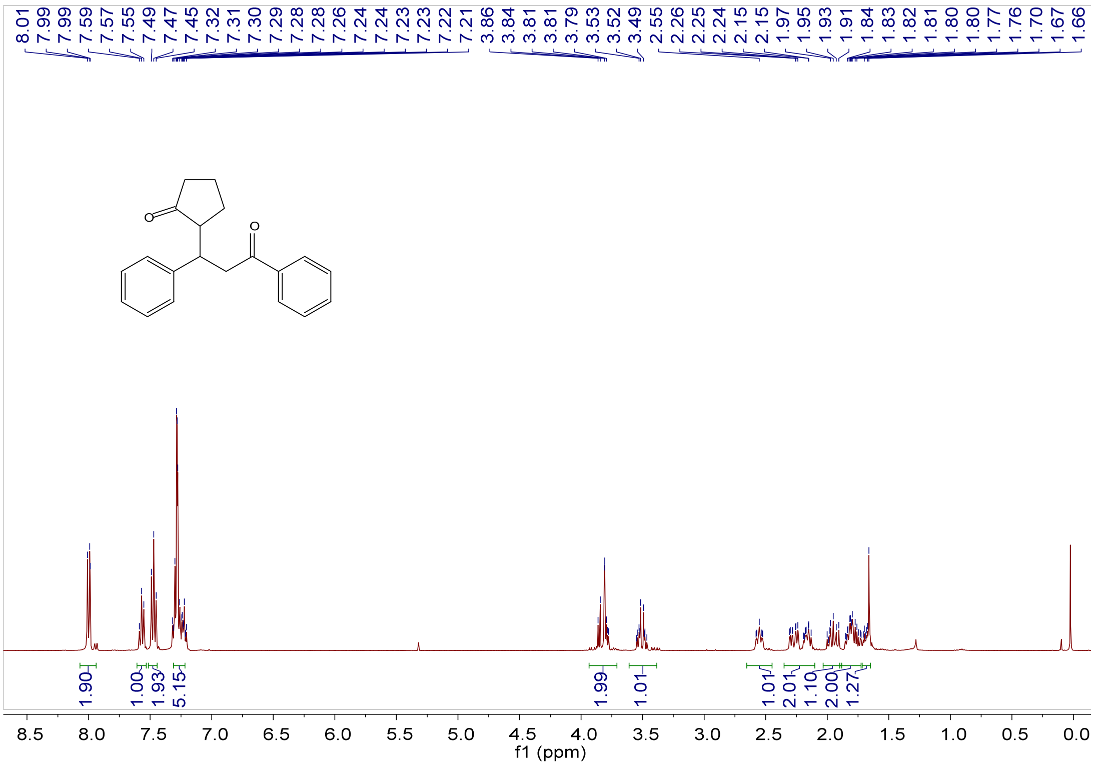

Supplementary Fig. 17 ^{1}H NMR spectra of compound **6a** in $CDCl_3$

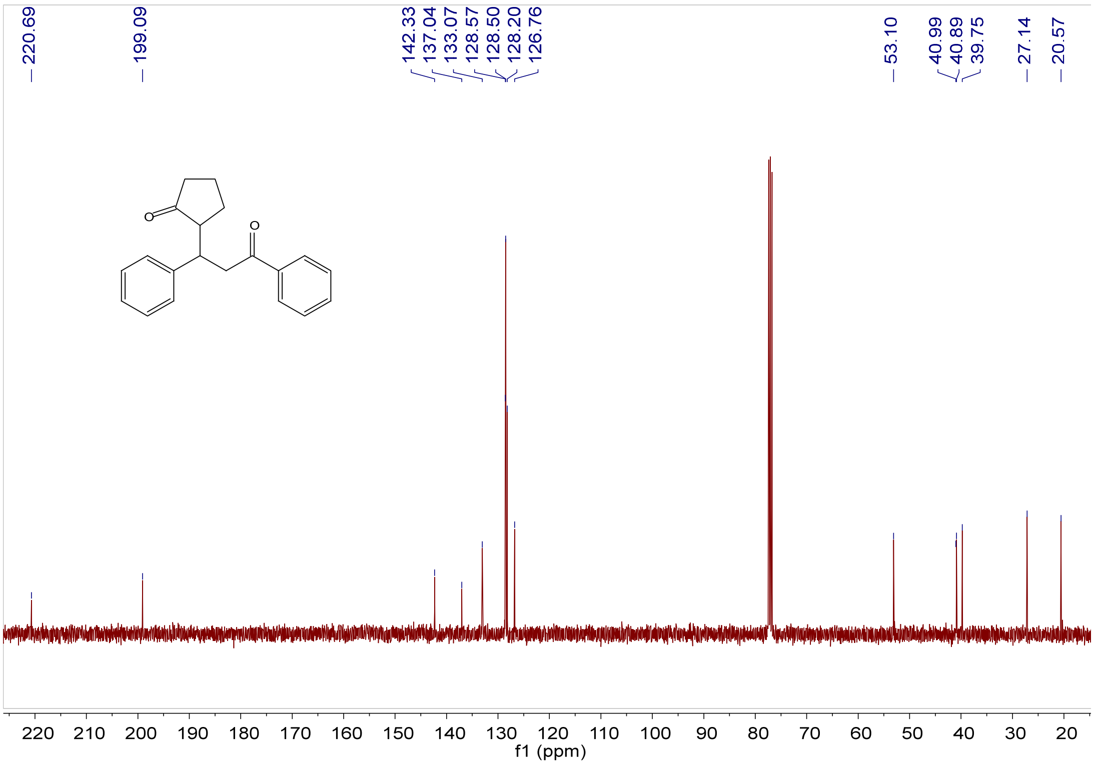

Supplementary Fig. 18 ^{13}C NMR spectra of compound **6a** in $CDCl_3$

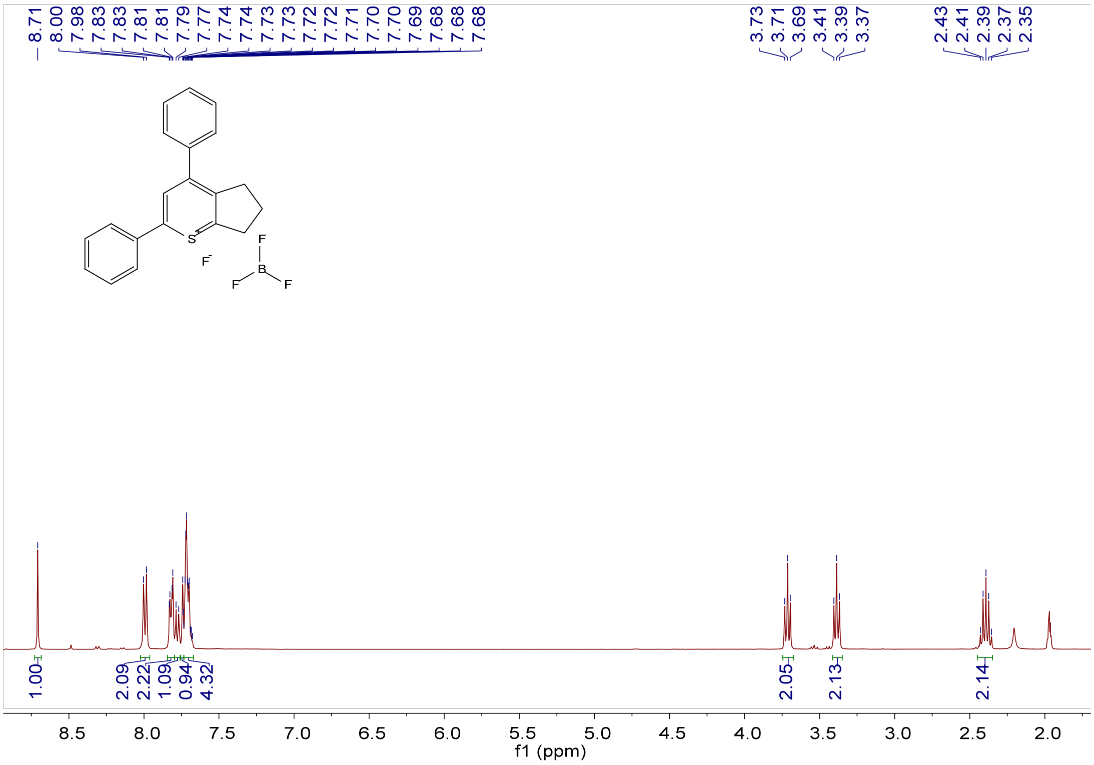

Supplementary Fig. 19 ^{1}H NMR spectra of compound **7a** in Acetonitrile-d_3

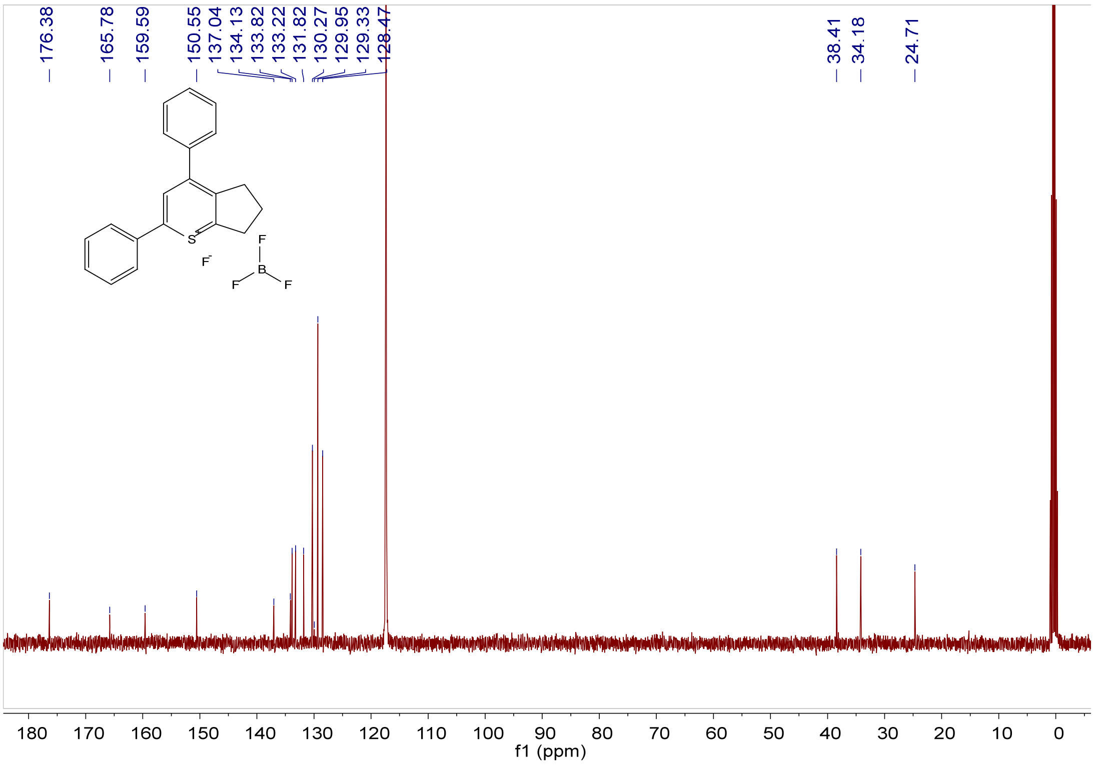

Supplementary Fig. 20 ^{13}C NMR spectra of compound **7a** in Acetonitrile-d_3

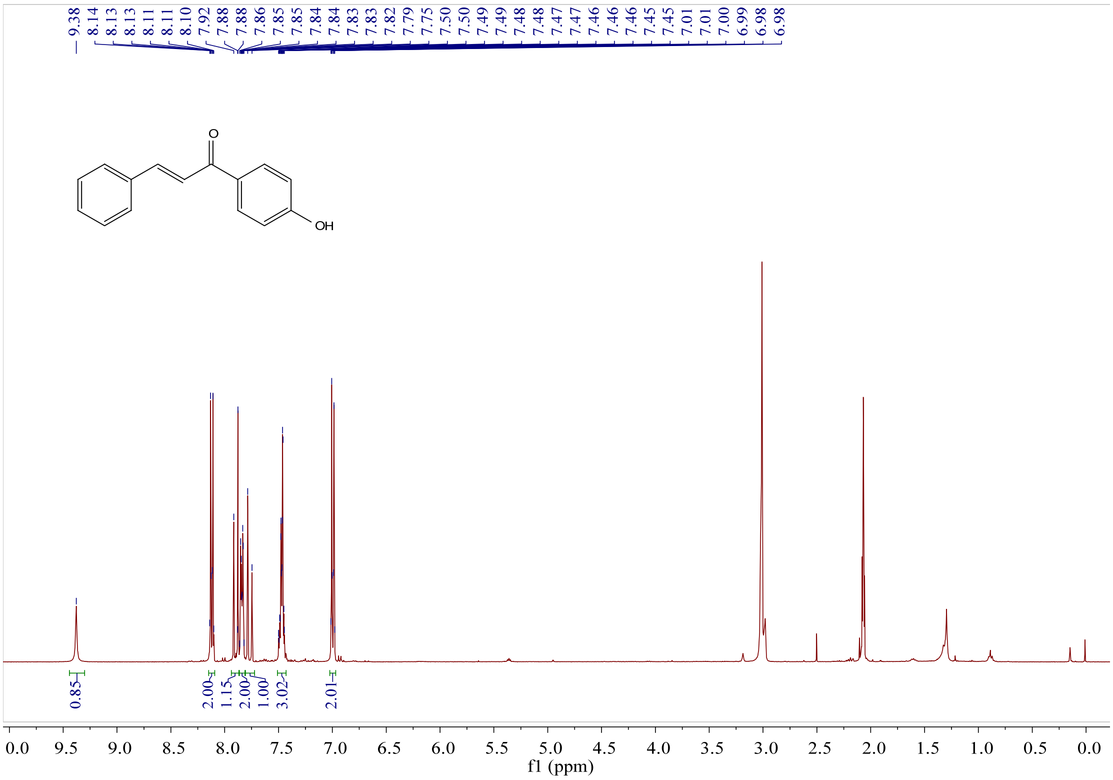

Supplementary Fig. 21 ^{1}H NMR spectra of compound ***1-(4-hydroxyphenyl)-3-phenylprop-2-en-1-one*** in Acetone-d_6

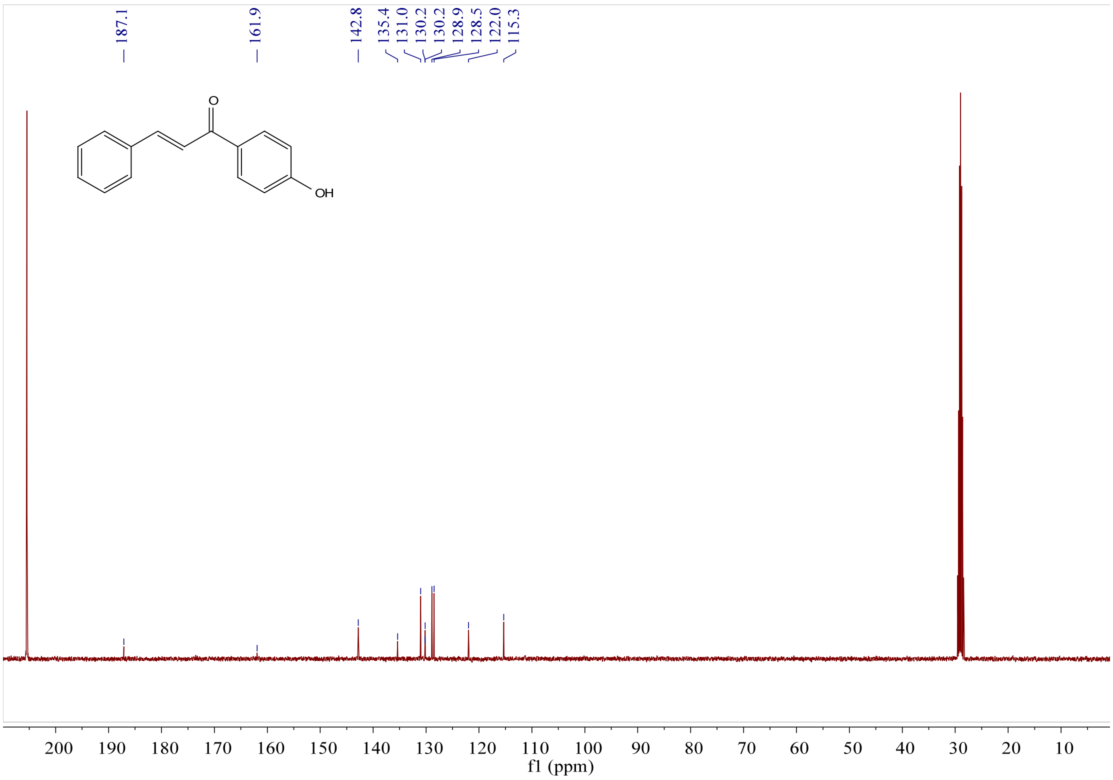

Supplementary Fig. 22 ^{13}C NMR spectra of compound ***1-(4-hydroxyphenyl)-3-phenylprop-2-en-1-one*** in Acetone-d_6

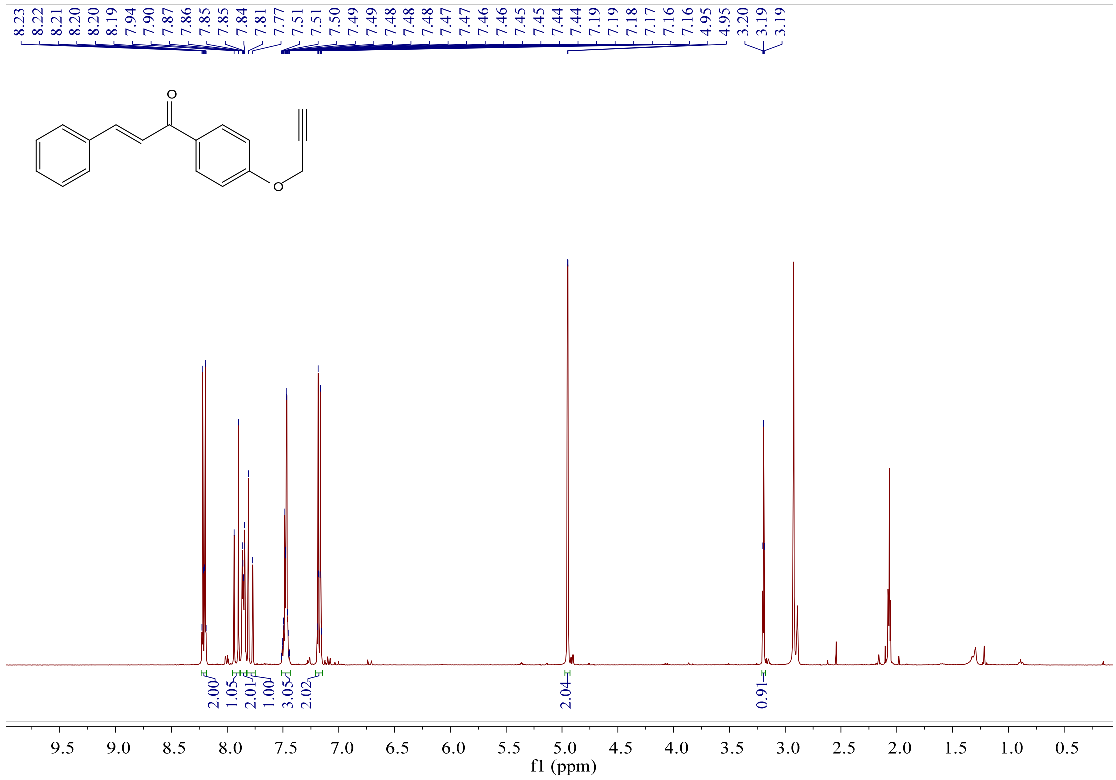

Supplementary Fig. 23 ^{1}H NMR spectra of compound **5b** in Acetone-d_6

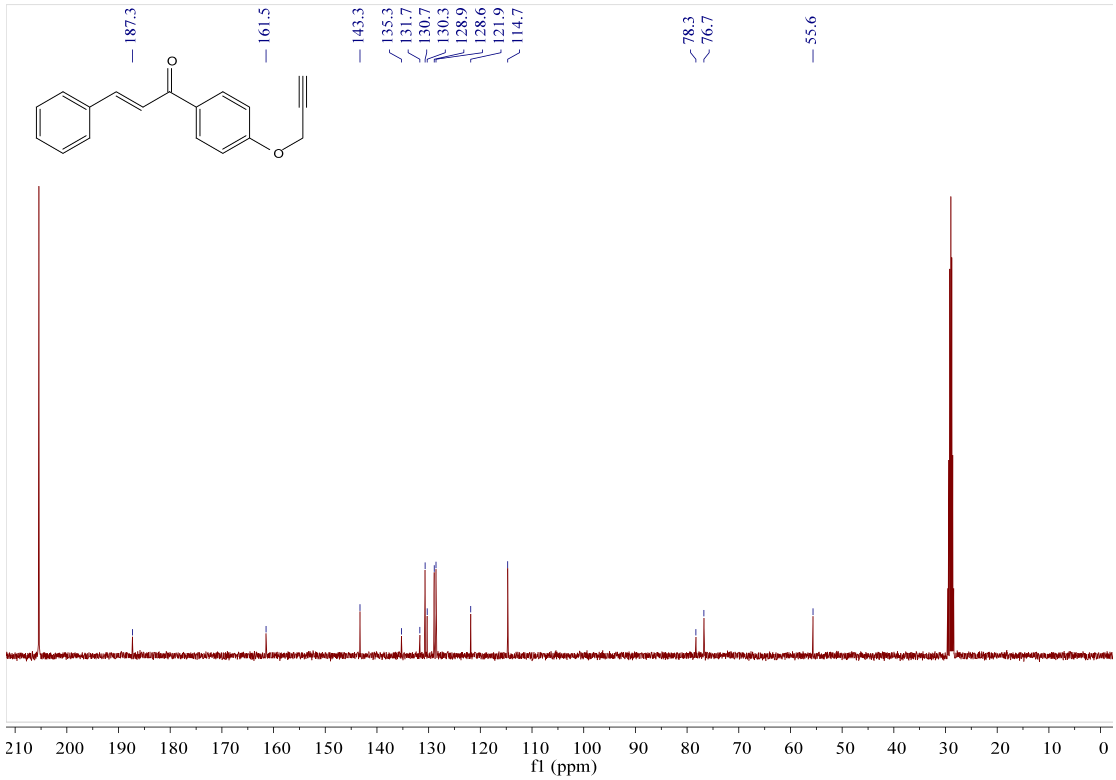

Supplementary Fig. 24 ^{13}C NMR spectra of compound **5b** in Acetone-d_6

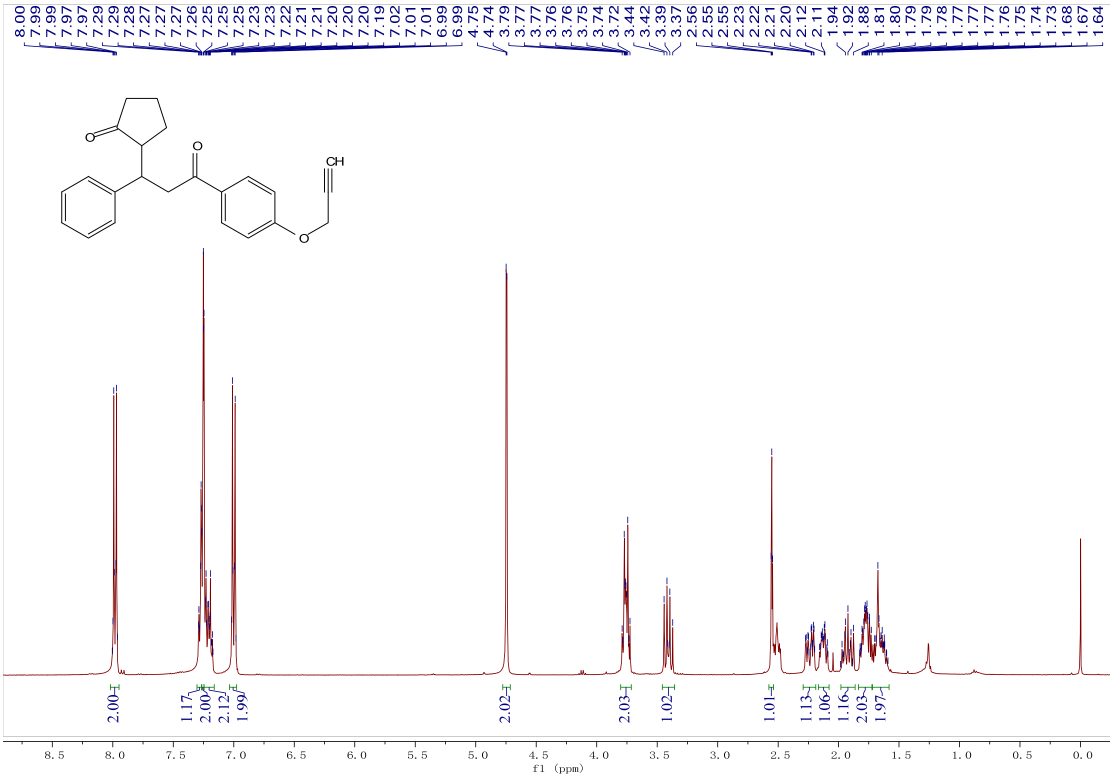

Supplementary Fig. 25 ^{1}H NMR spectra of compound **6b** in Chloroform-*d*

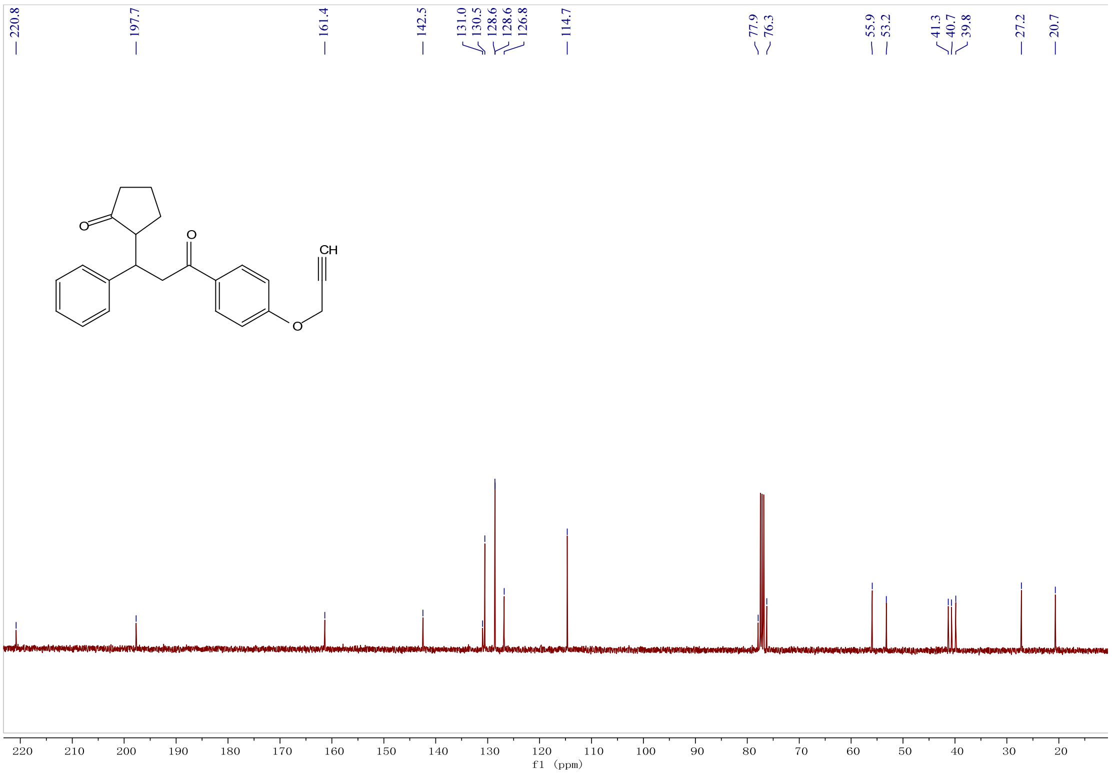

Supplementary Fig. 26 ^{13}C NMR spectra of compound **6b** in Chloroform-*d*

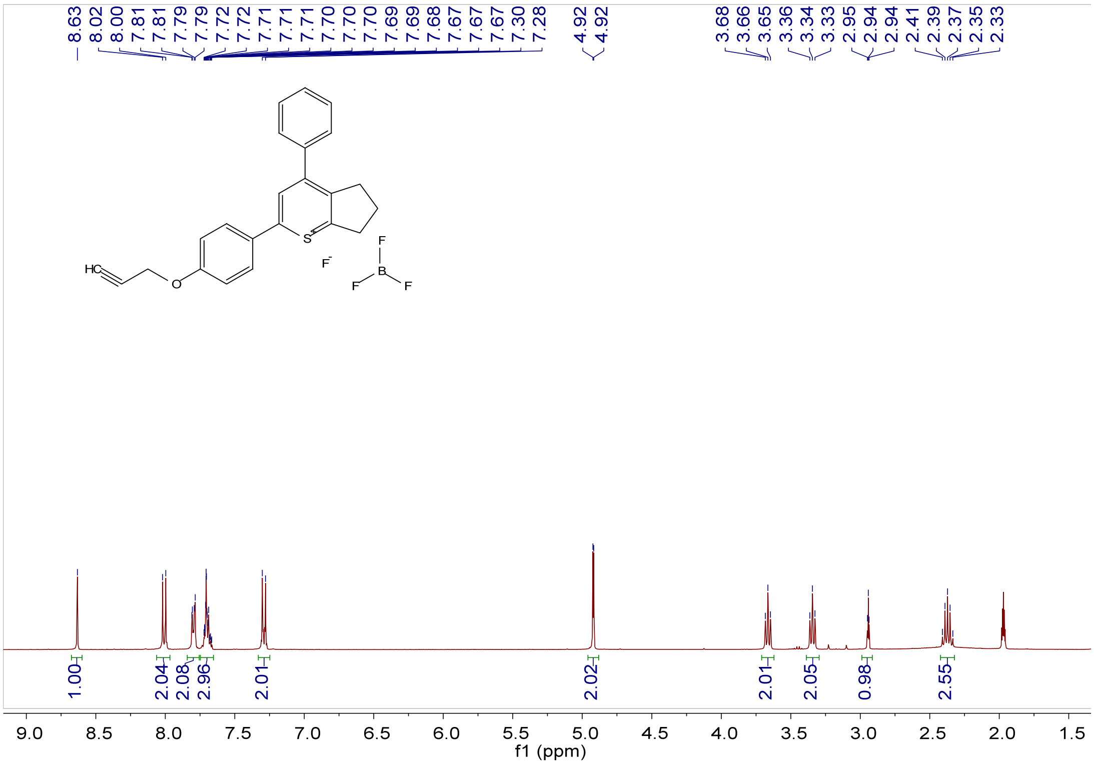

Supplementary Fig. 27 ^{1}H NMR spectra of compound **7b** in Acetonitrile-d_3

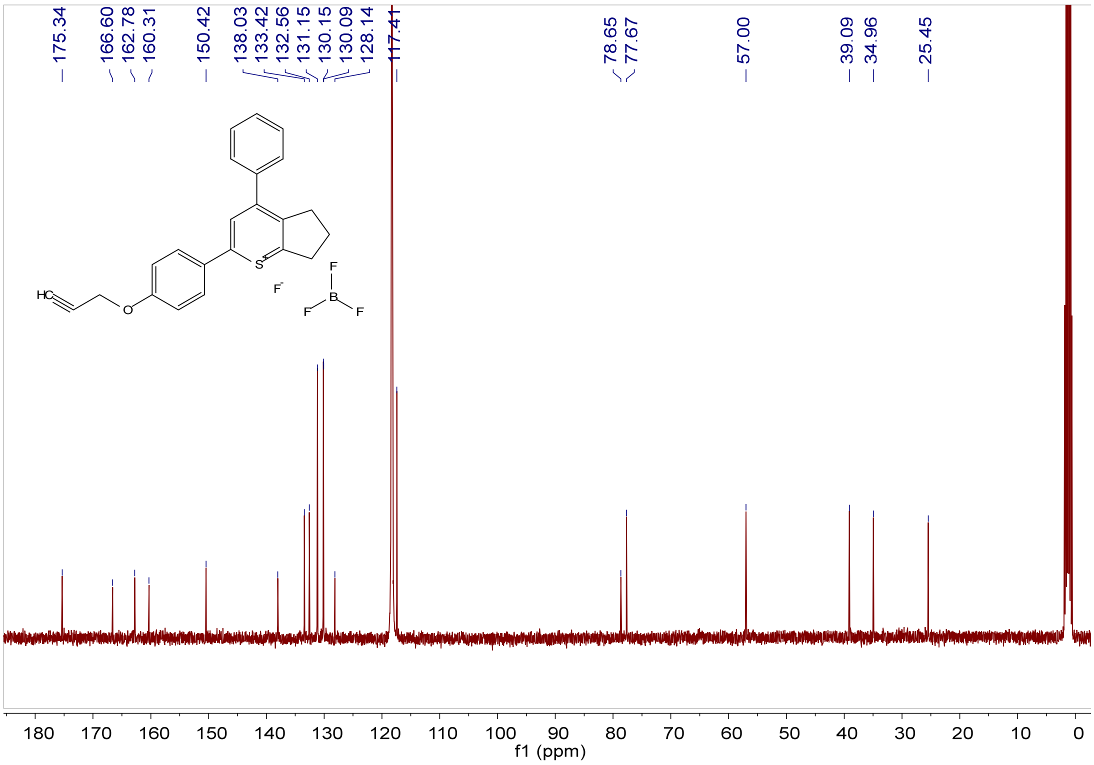

Supplementary Fig. 28 ^{13}C NMR spectra of compound **7b** in Acetonitrile-d_3

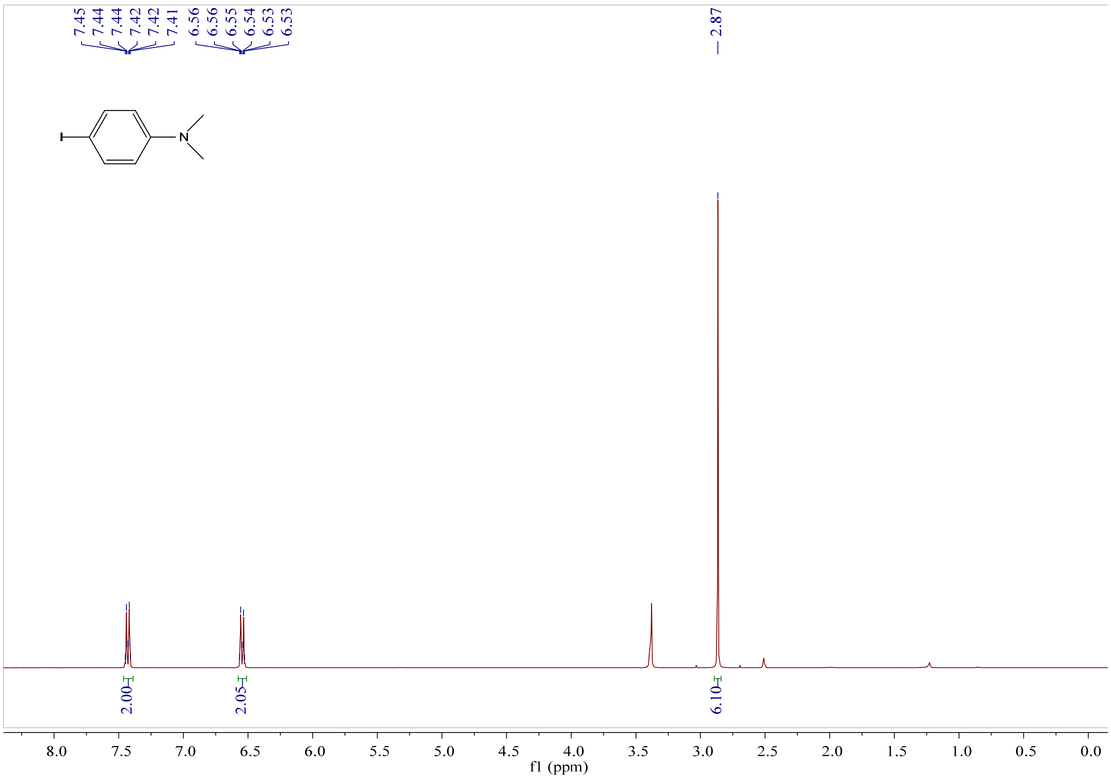

Supplementary Fig. 29 ^{1}H NMR spectra of compound ***2, 4-iodo-N, N-dimethylaniline*** in DMSO-d_6

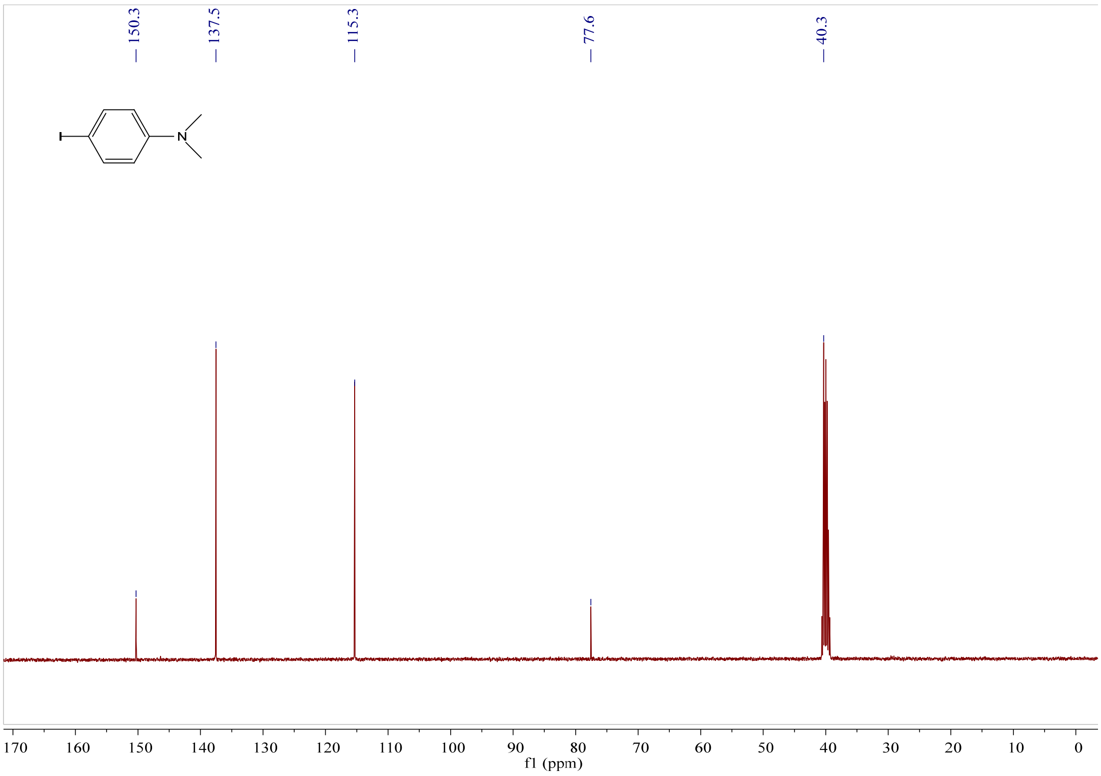

Supplementary Fig. 30 ^{13}C NMR spectra of compound ***2, 4-iodo-N, N-dimethylaniline*** in DMSO-d_6

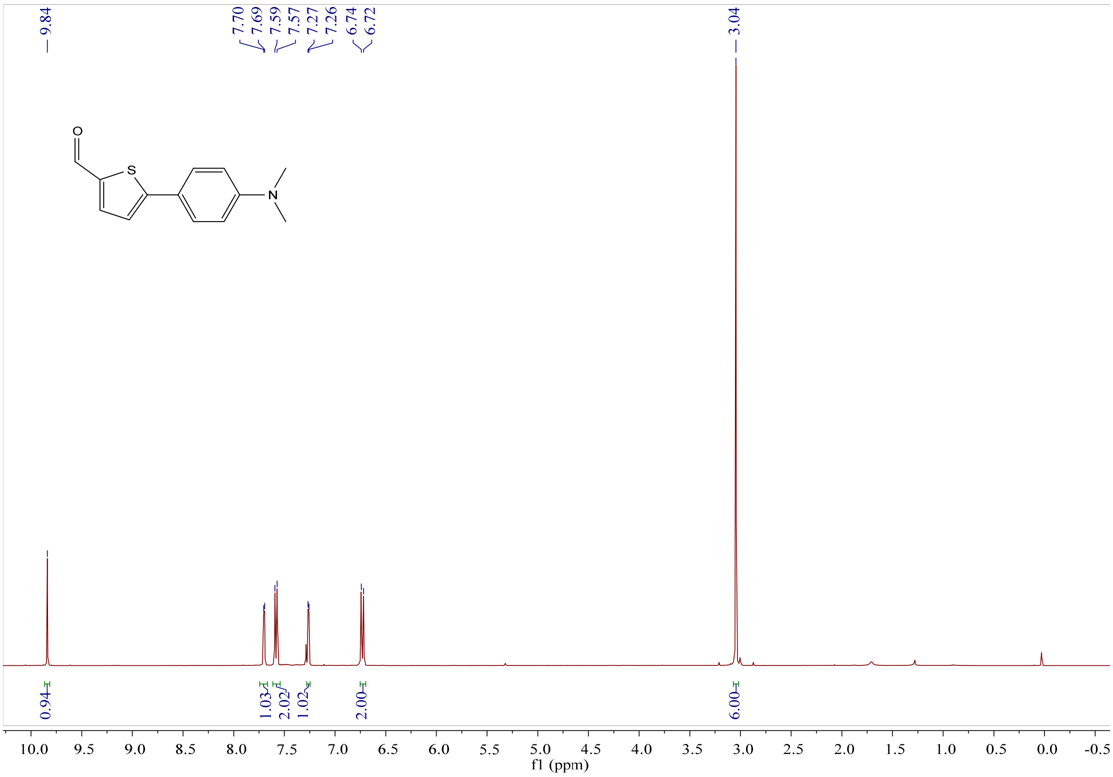

Supplementary Fig. 31 ^{1}H NMR spectra of compound ***5-(4-(dimethylamino)phenyl) thiophene-2-carbaldehyd*** in Chloroform-*d*

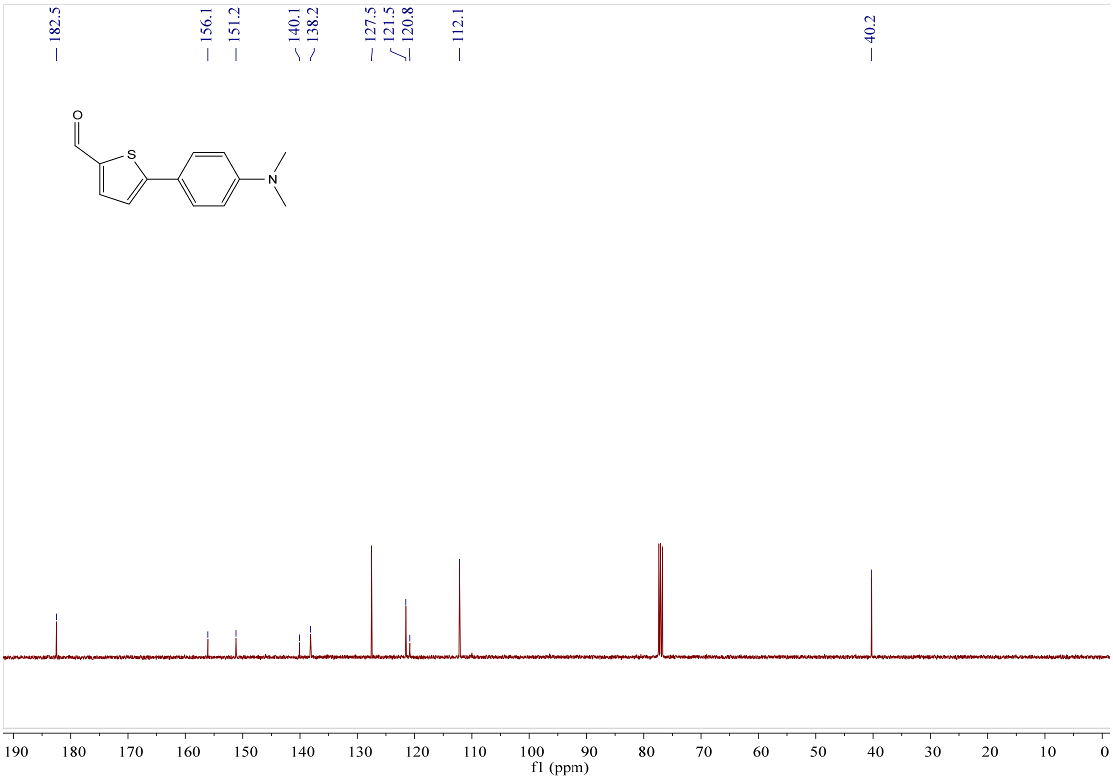

Supplementary Fig. 32 ^{13}C NMR spectra of compound ***5-(4-(dimethylamino)phenyl) thiophene-2-carbaldehyd*** in Chloroform-*d*

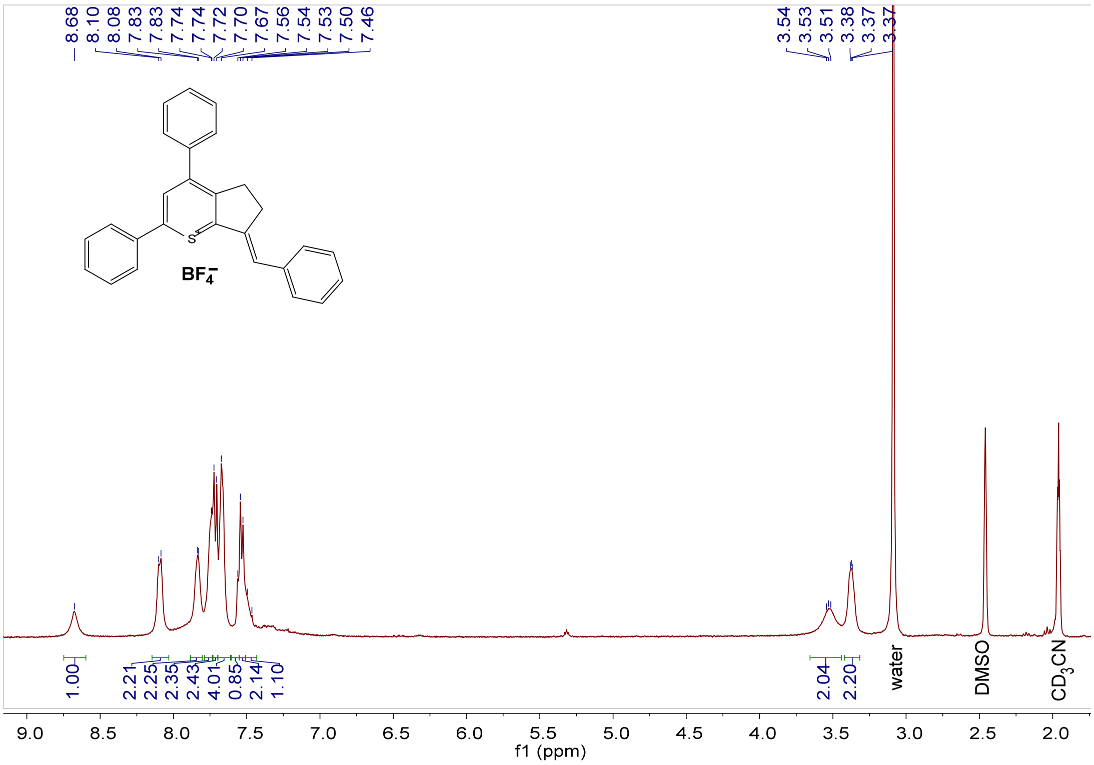

Supplementary Fig. 33 ^{1}H NMR spectra of compound **3a** in Acetonitrile-d_3 and DMSO-d_6

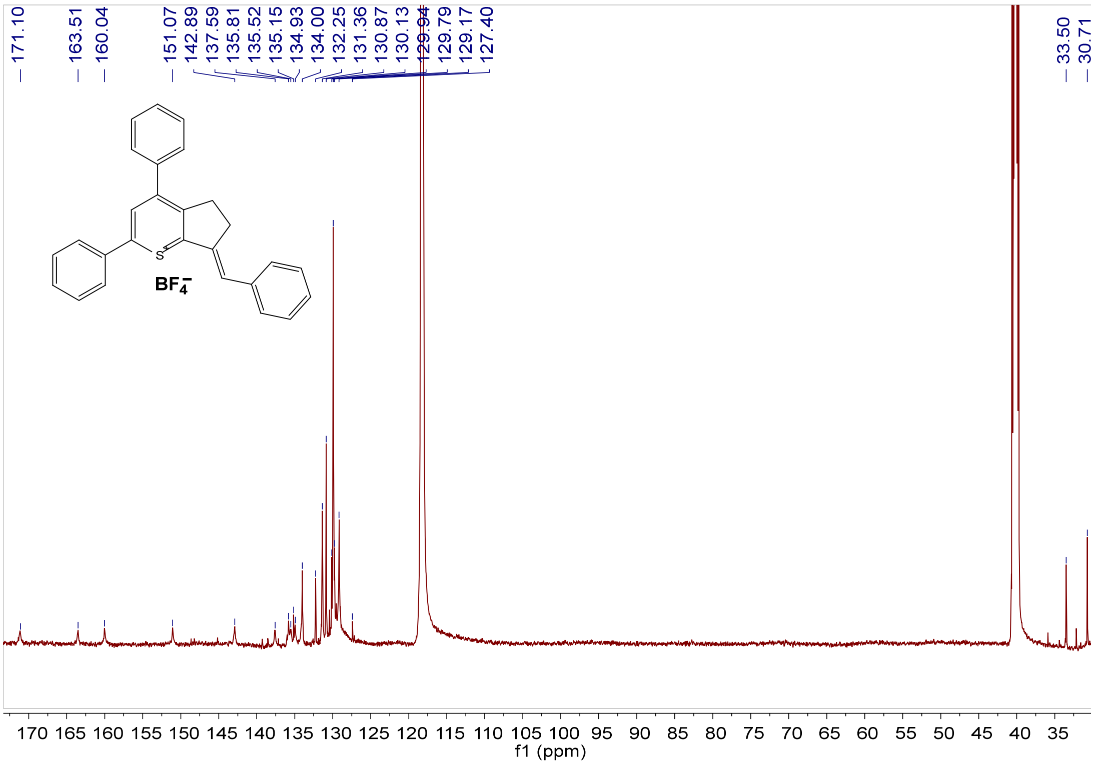

Supplementary Fig. 34 ^{13}C NMR spectra of compound **3a** in Acetonitrile-d_3 and DMSO-d_6

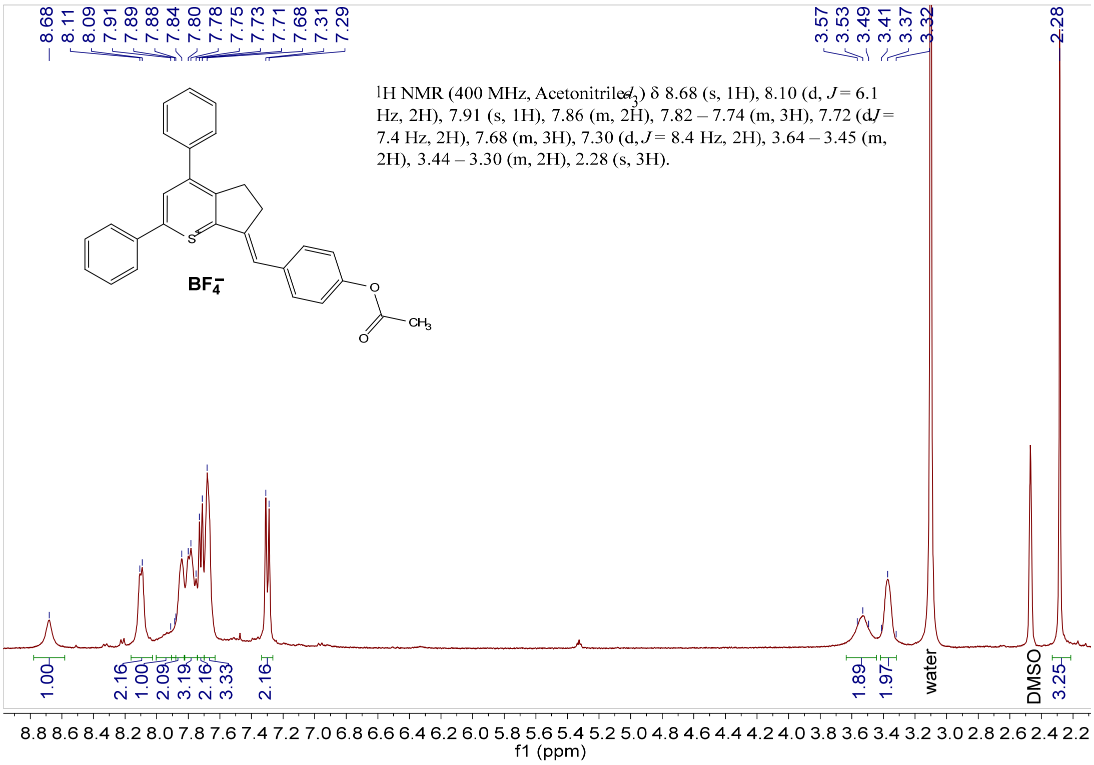

Supplementary Fig. 35 ^{1}H NMR spectra of compound **3b** in Acetonitrile-d_3 and DMSO-d_6

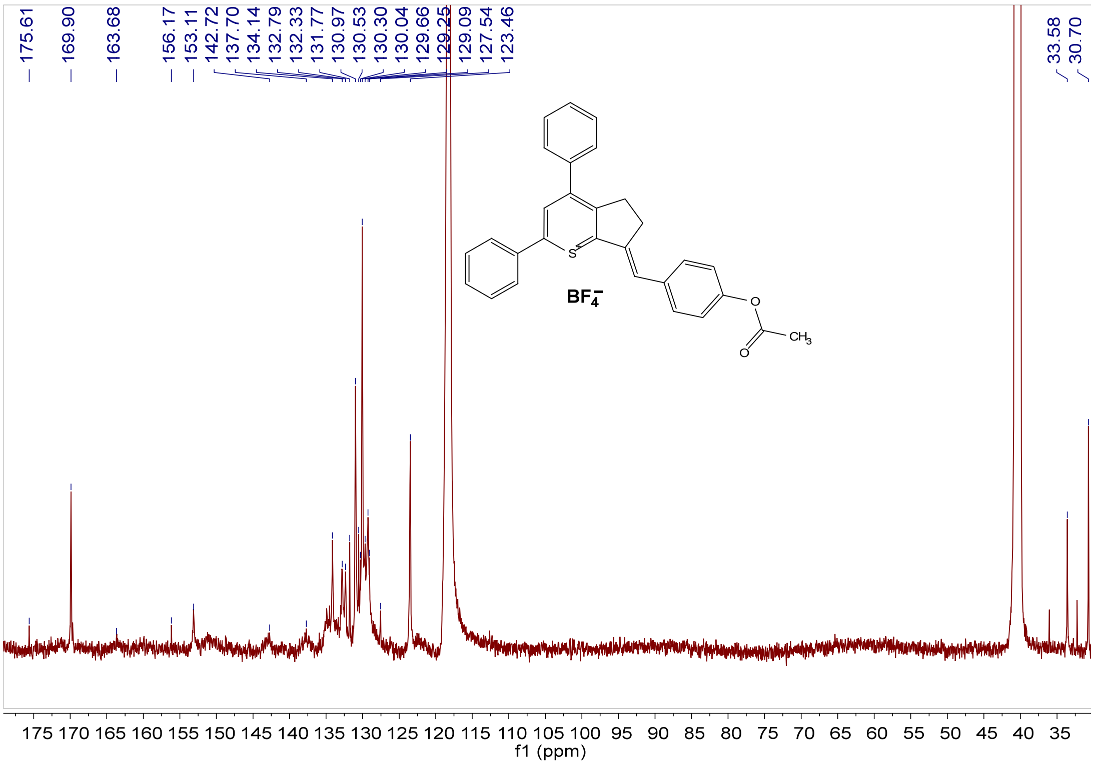

Supplementary Fig. 36 ^{13}C NMR spectra of compound **3b** in Acetonitrile-d_3 and DMSO-d_6

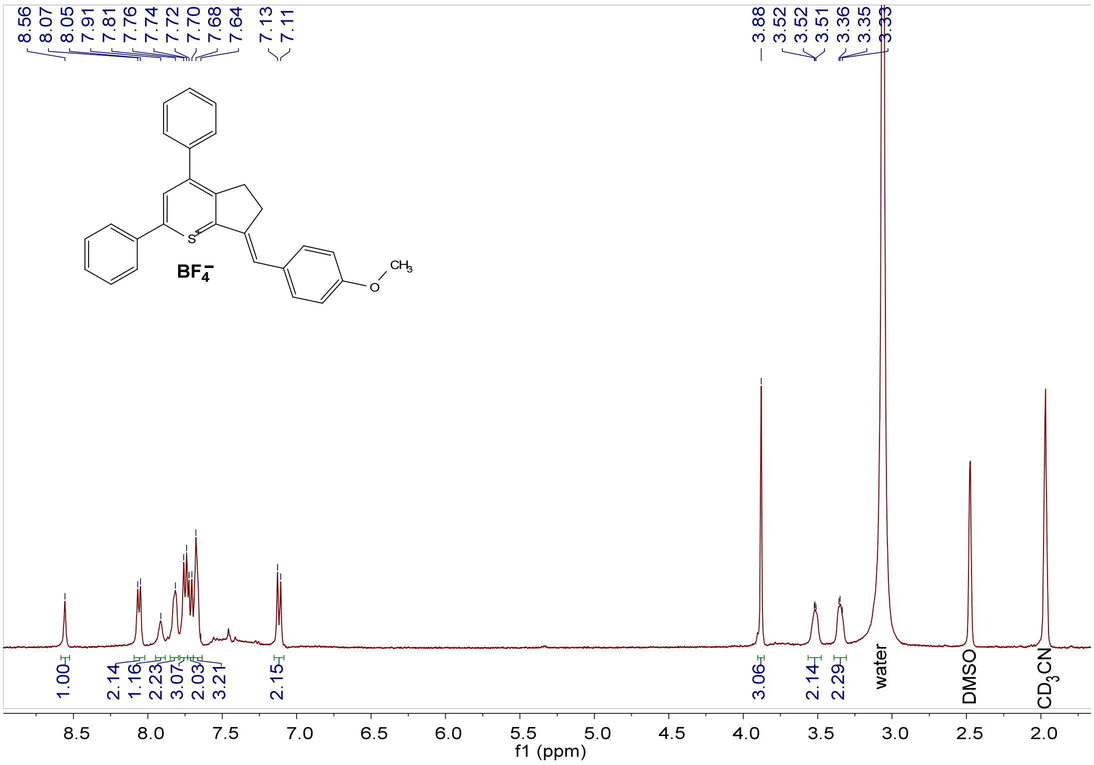

Supplementary Fig. 37 ^{1}H NMR spectra of compound **3c** in Acetonitrile-d_3 and DMSO-d_6

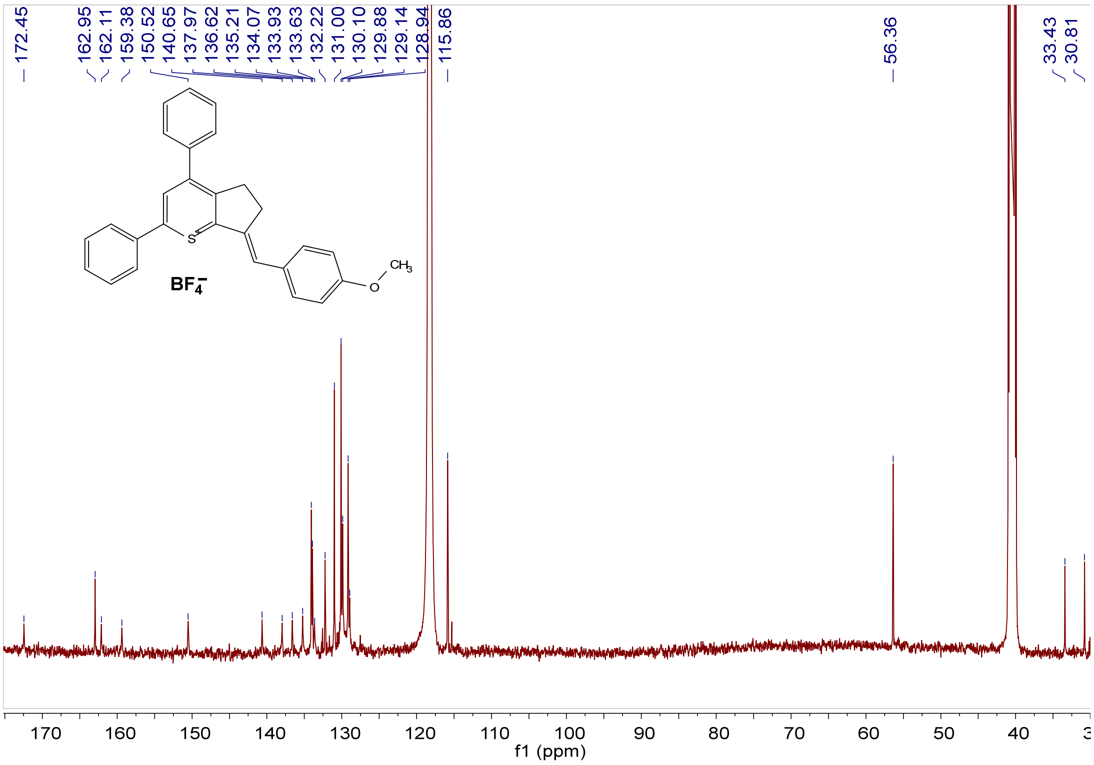

Supplementary Fig. 38 ^{13}C NMR spectra of compound **3c** in Acetonitrile-d_3 and DMSO-d_6

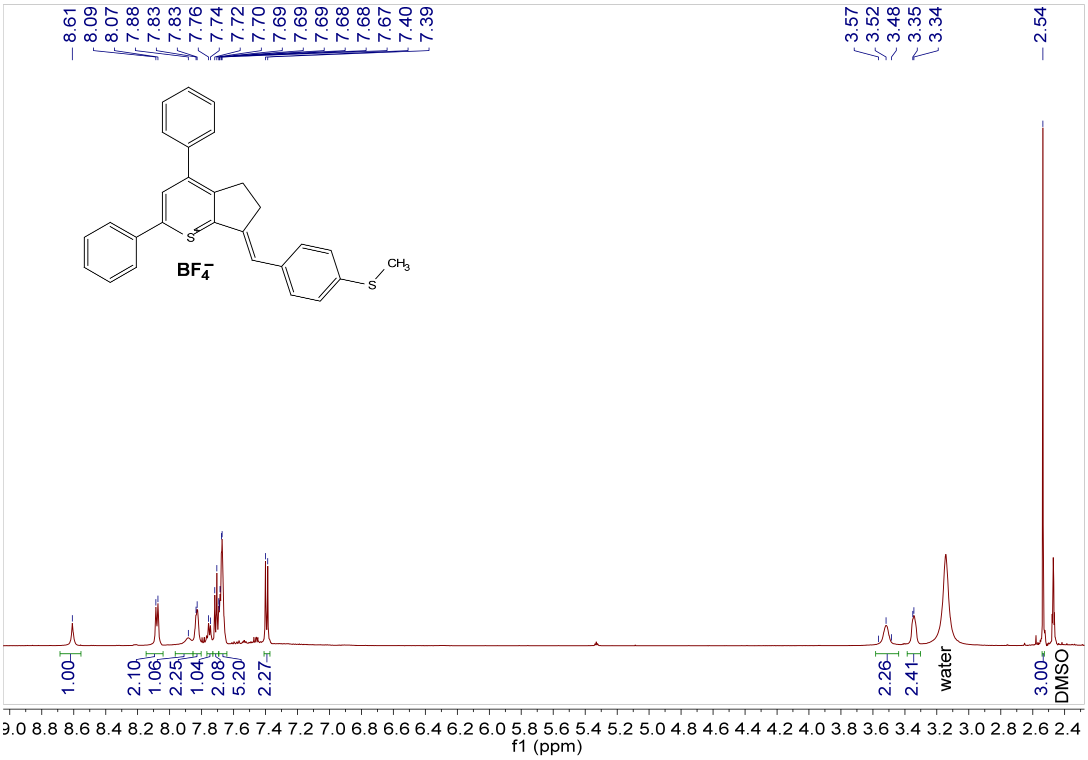

Supplementary Fig. 39 ^{1}H NMR spectra of compound **3d** in Acetonitrile-d_3 and DMSO-d_6

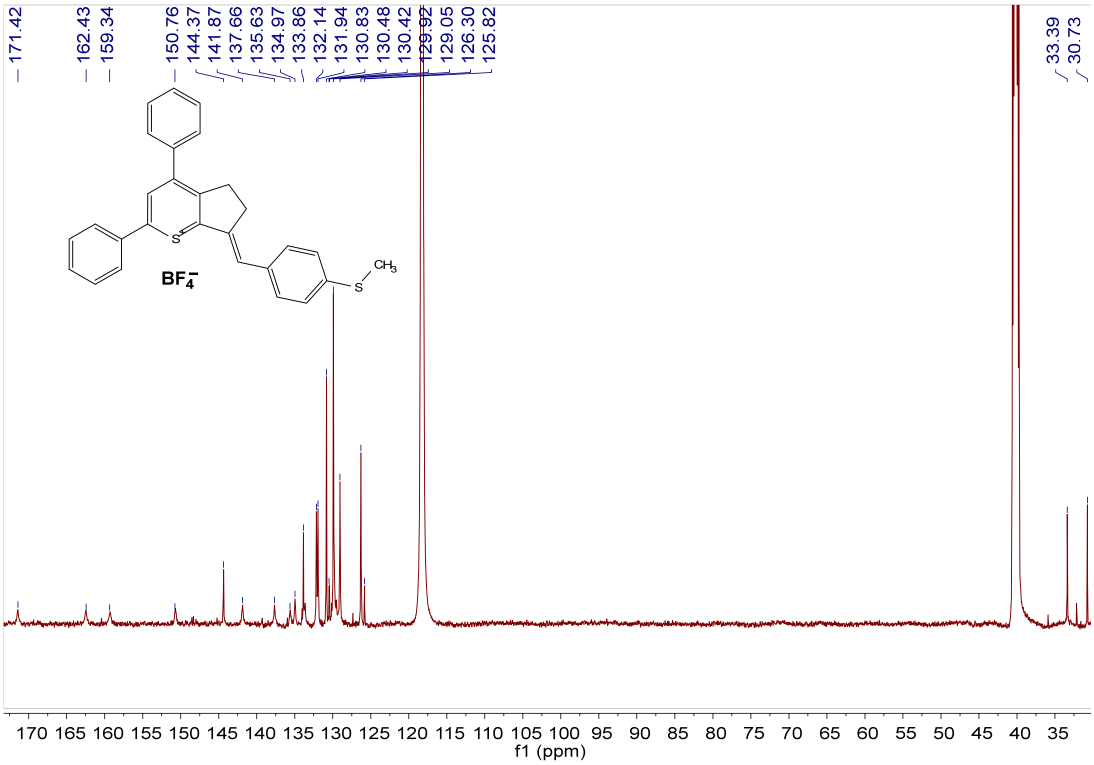

Supplementary Fig. 40 ^{13}C NMR spectra of compound **3d** in Acetonitrile-d_3 and DMSO-d_6

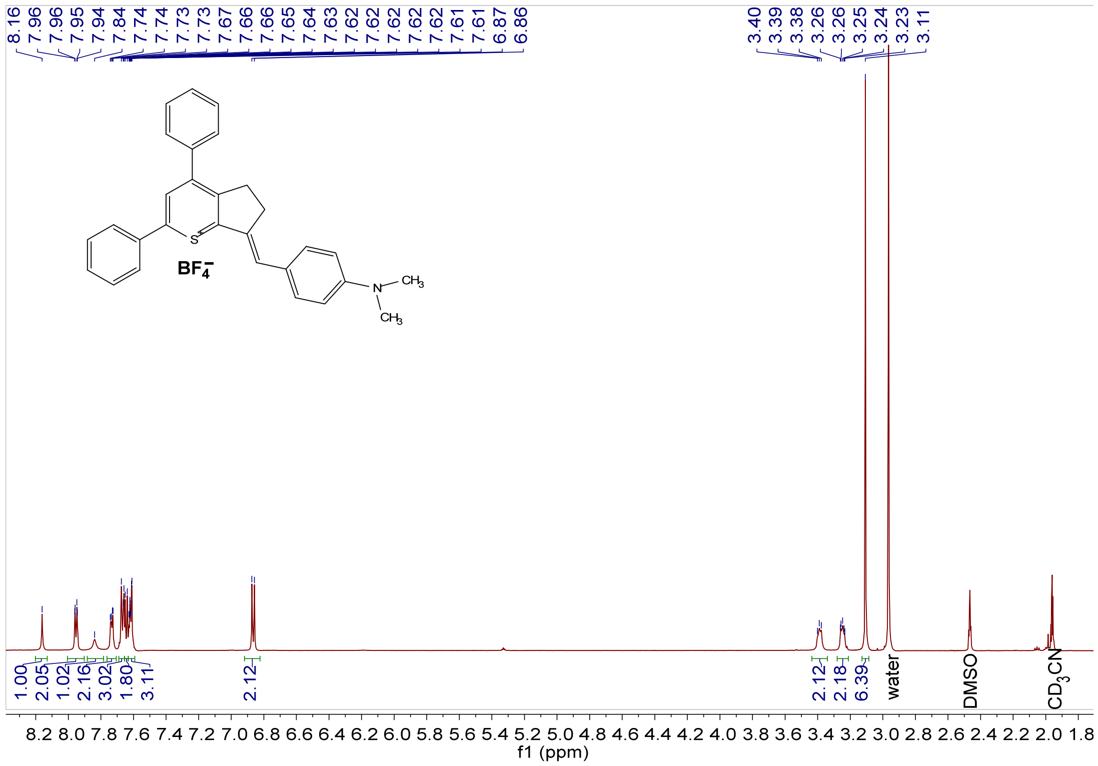

Supplementary Fig. 41 ^{1}H NMR spectra of compound **3e** in Acetonitrile-d_3 and DMSO-d_6

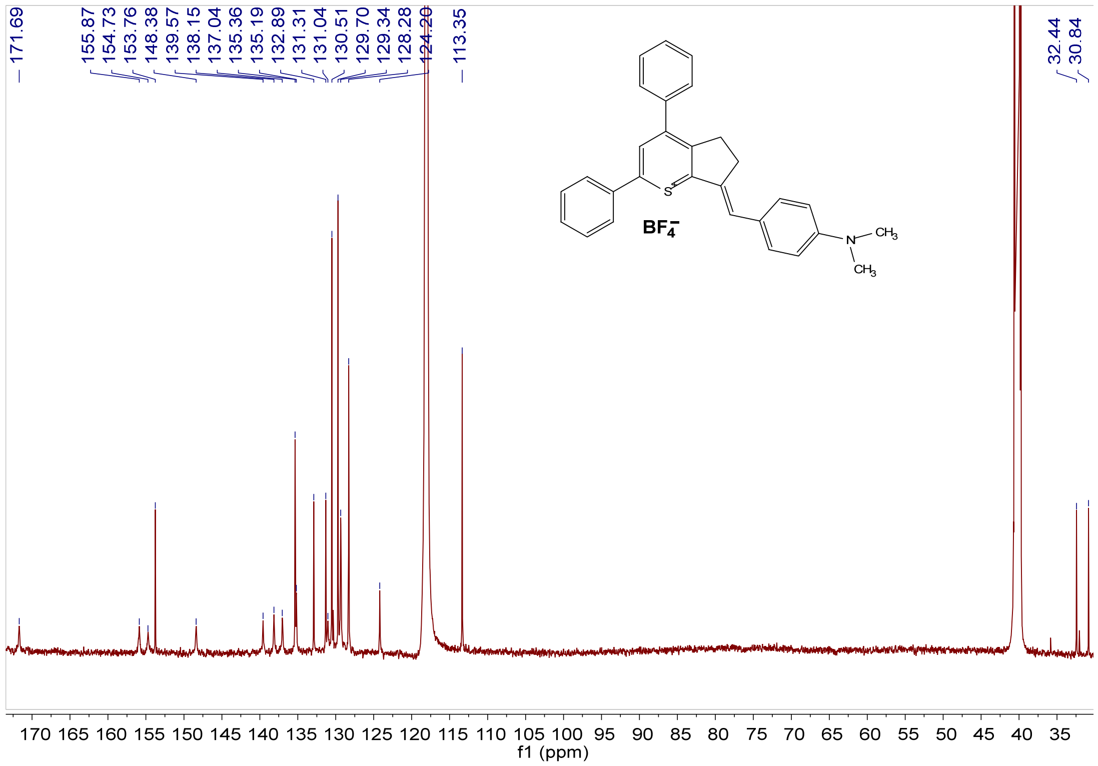

Supplementary Fig. 42 ^{13}C NMR spectra of compound **3e** in Acetonitrile-d_3 and DMSO-d_6

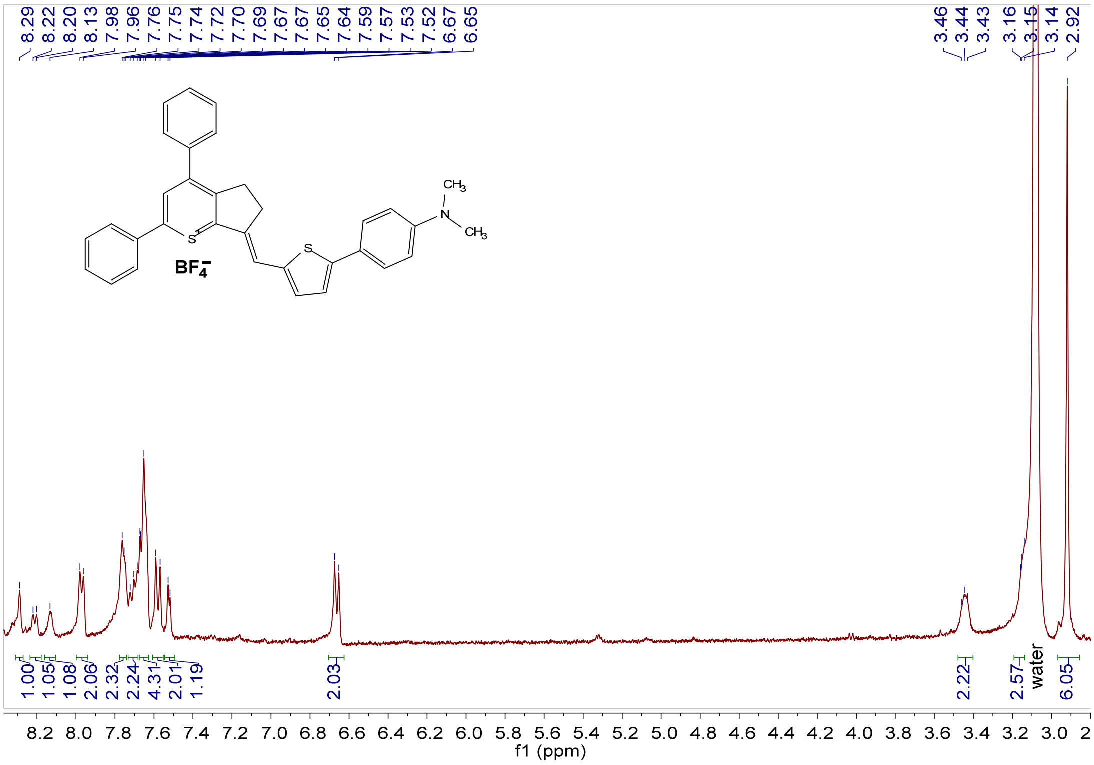

Supplementary Fig. 43 ^{1}H NMR spectra of compound **3f** in Acetonitrile-d_3 and DMSO-d_6

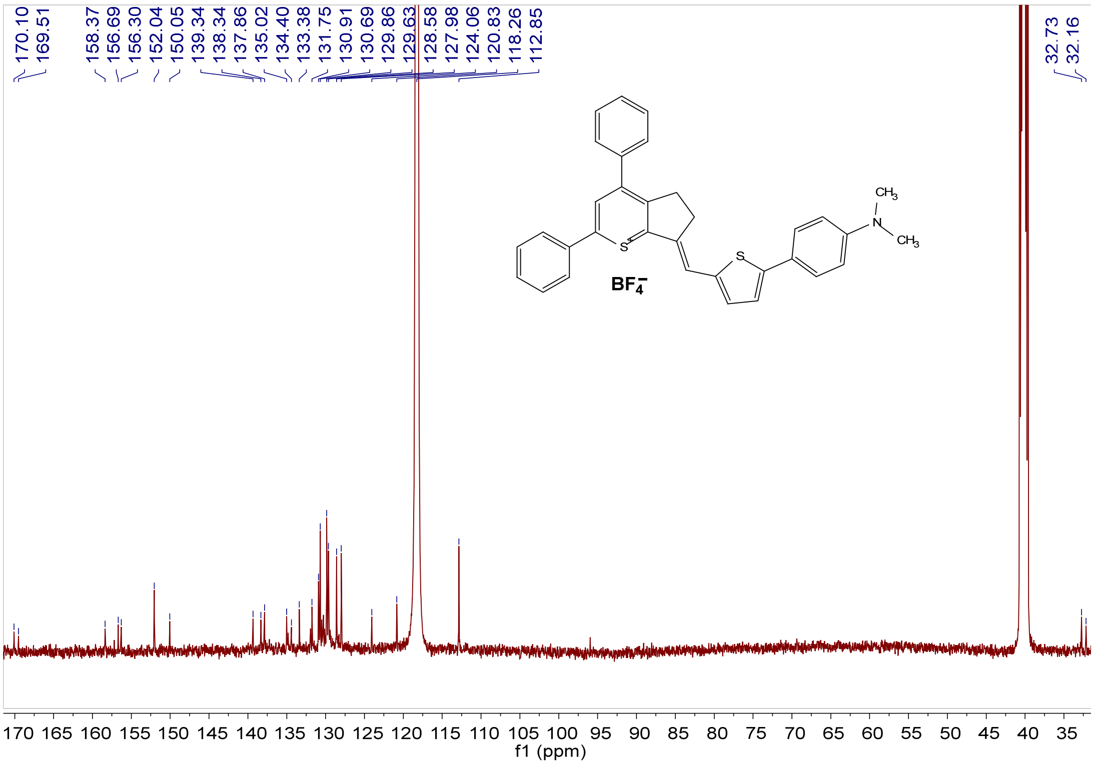

Supplementary Fig. 44 ^{13}C NMR spectra of compound **3f** in Acetonitrile-d_3 and DMSO-d_6

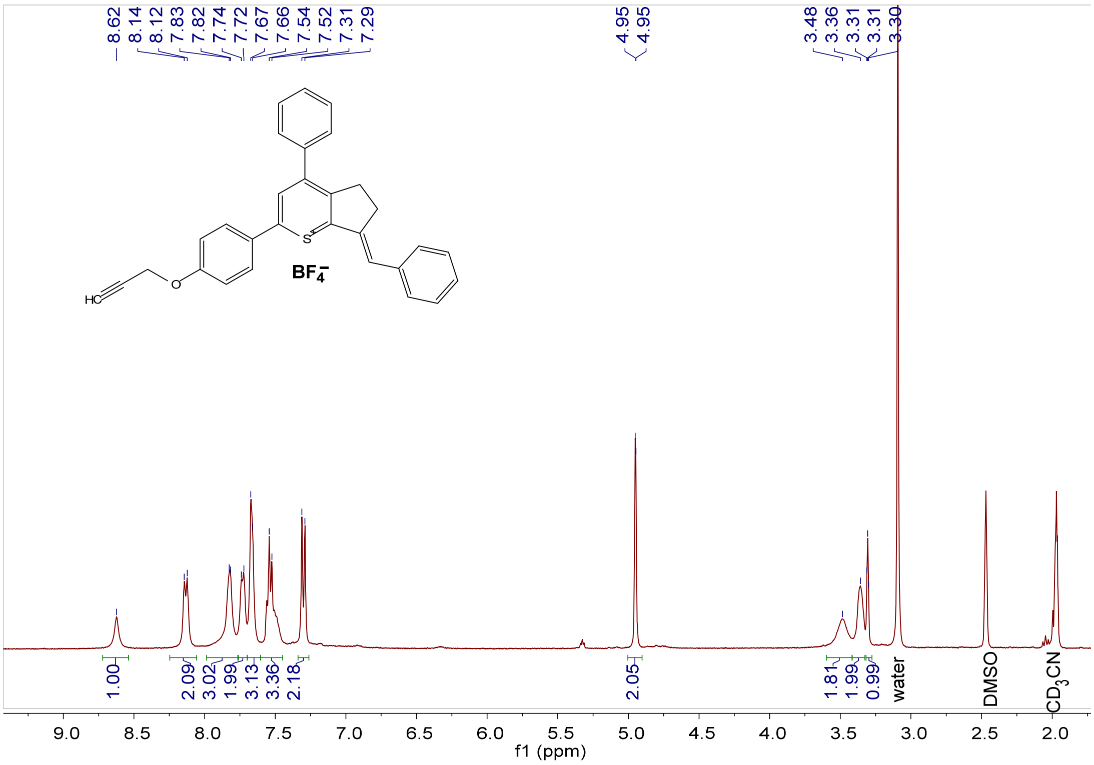

Supplementary Fig. 45 ^{1}H NMR spectra of compound **3g** in Acetonitrile-d_3 and DMSO-d_6

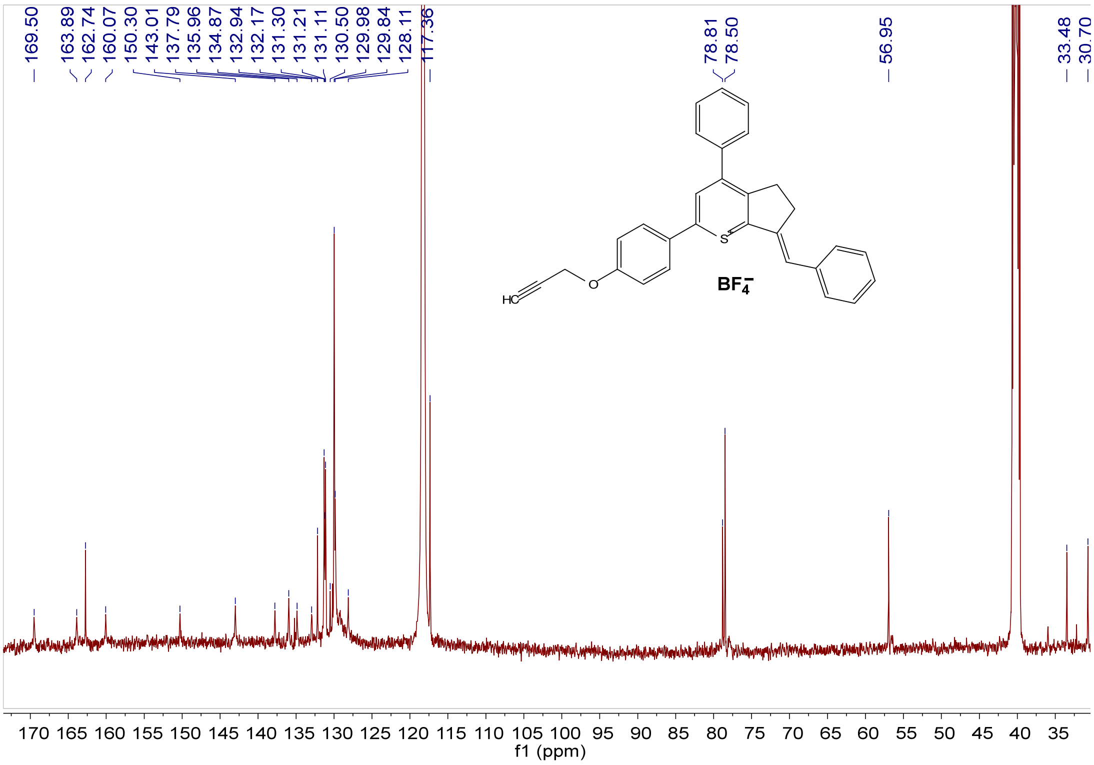

Supplementary Fig. 46 ^{13}C NMR spectra of compound **3g** in Acetonitrile-d_3 and DMSO-d_6

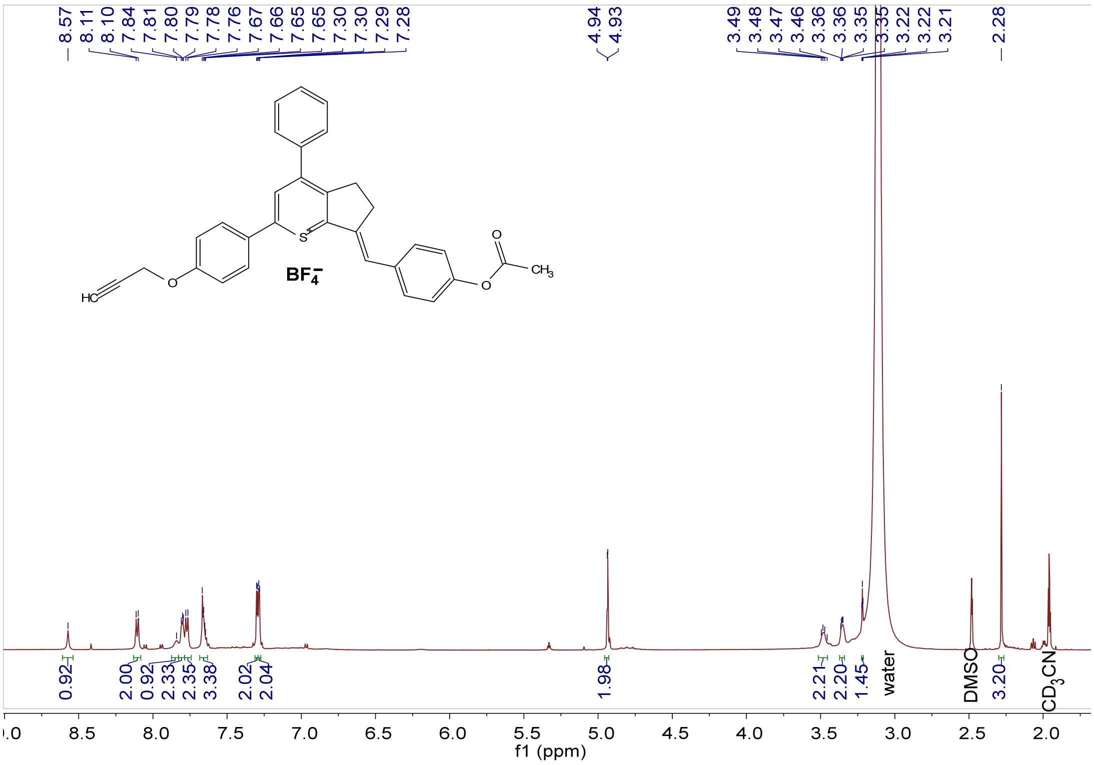

Supplementary Fig. 47 ^{1}H NMR spectra of compound **3h** in Acetonitrile-d_3 and DMSO-d_6

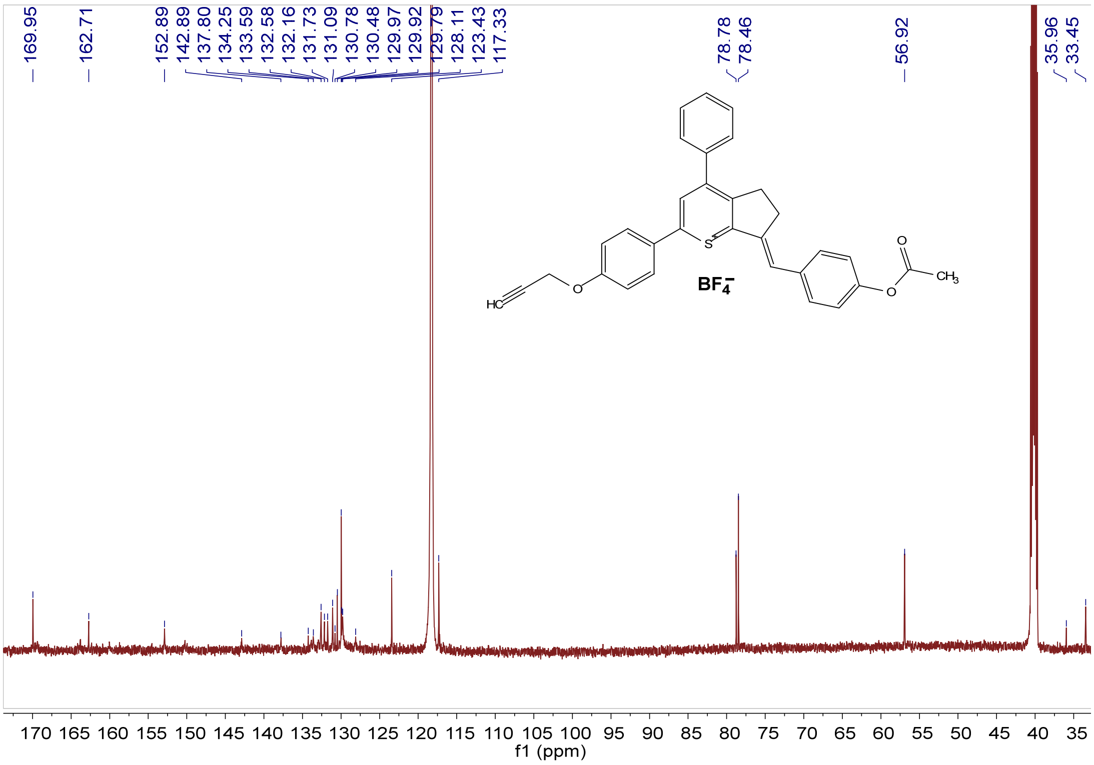

Supplementary Fig. 48 ^{13}C NMR spectra of compound **3h** in Acetonitrile-d_3 and DMSO-d_6

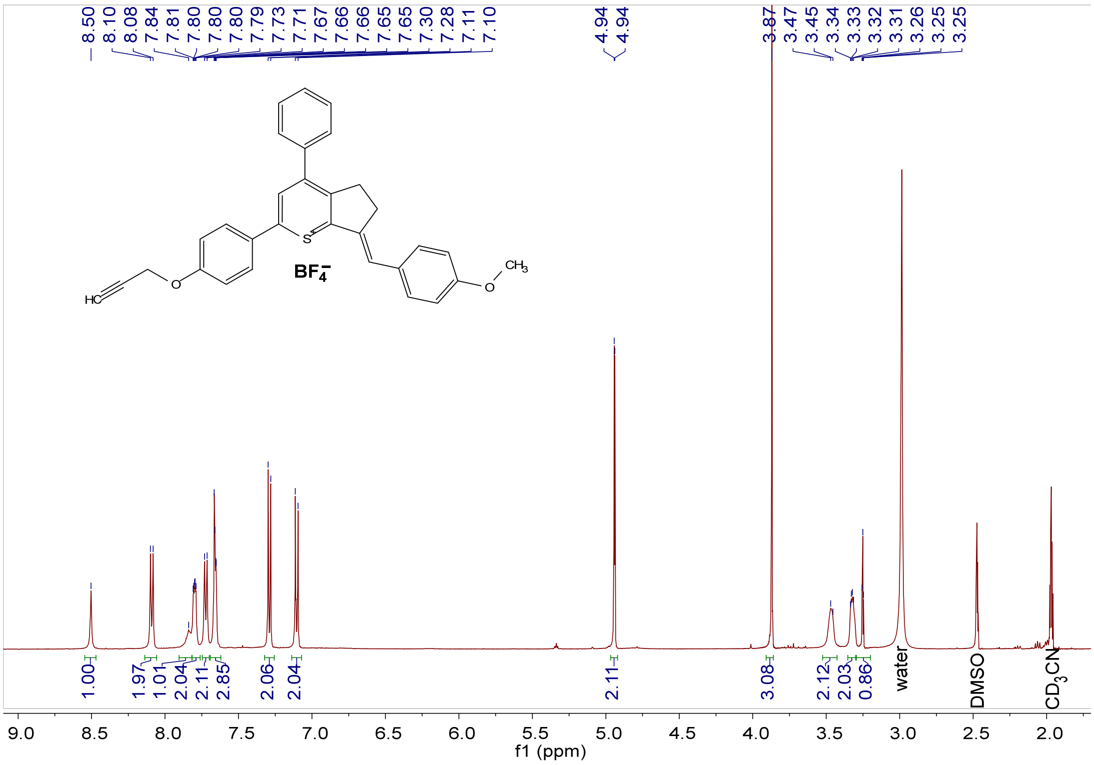

Supplementary Fig. 49 ^{1}H NMR spectra of compound **3i** in Acetonitrile-d_3 and DMSO-d_6

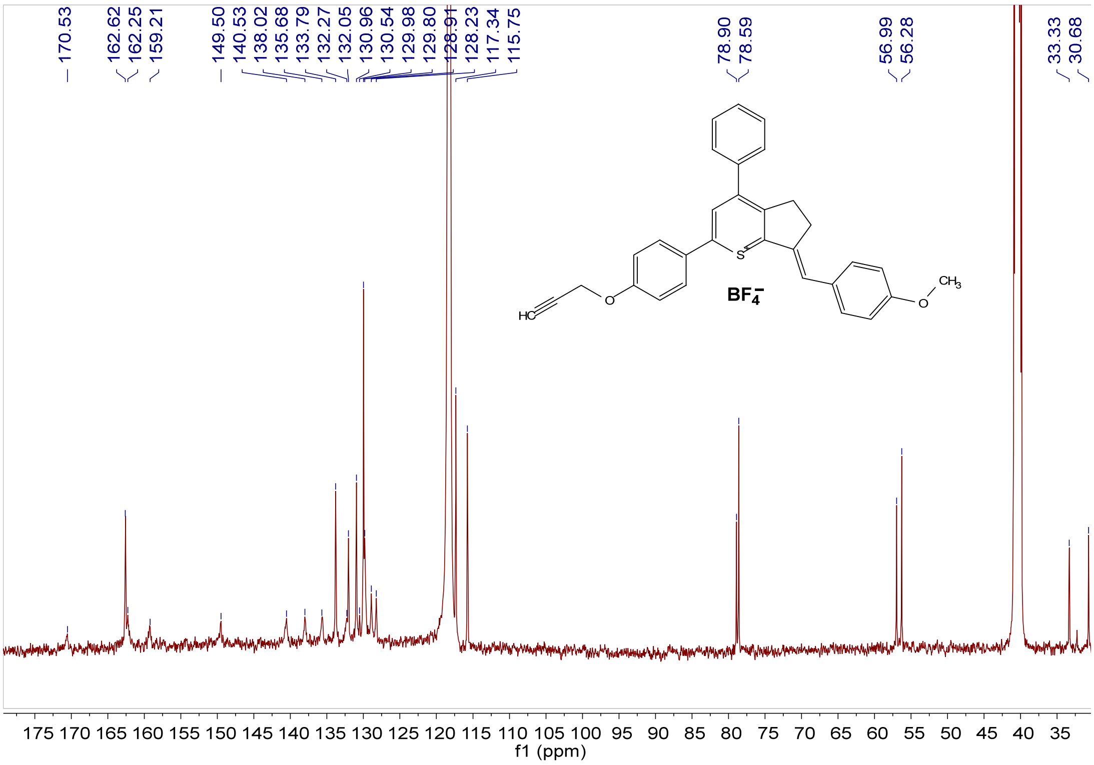

Supplementary Fig. 50 ^{13}C NMR spectra of compound **3i** in Acetonitrile-d_3 and DMSO-d_6

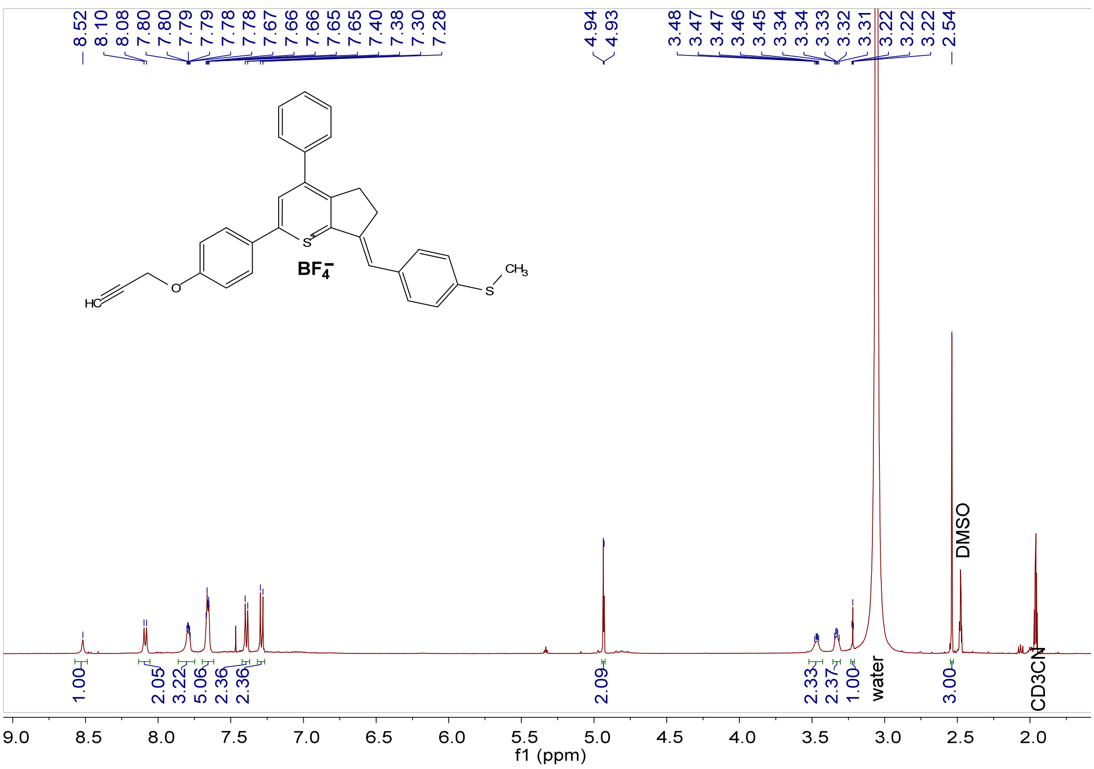

Supplementary Fig. 51 ^{1}H NMR spectra of compound **3j** in Acetonitrile-d_3 and DMSO-d_6

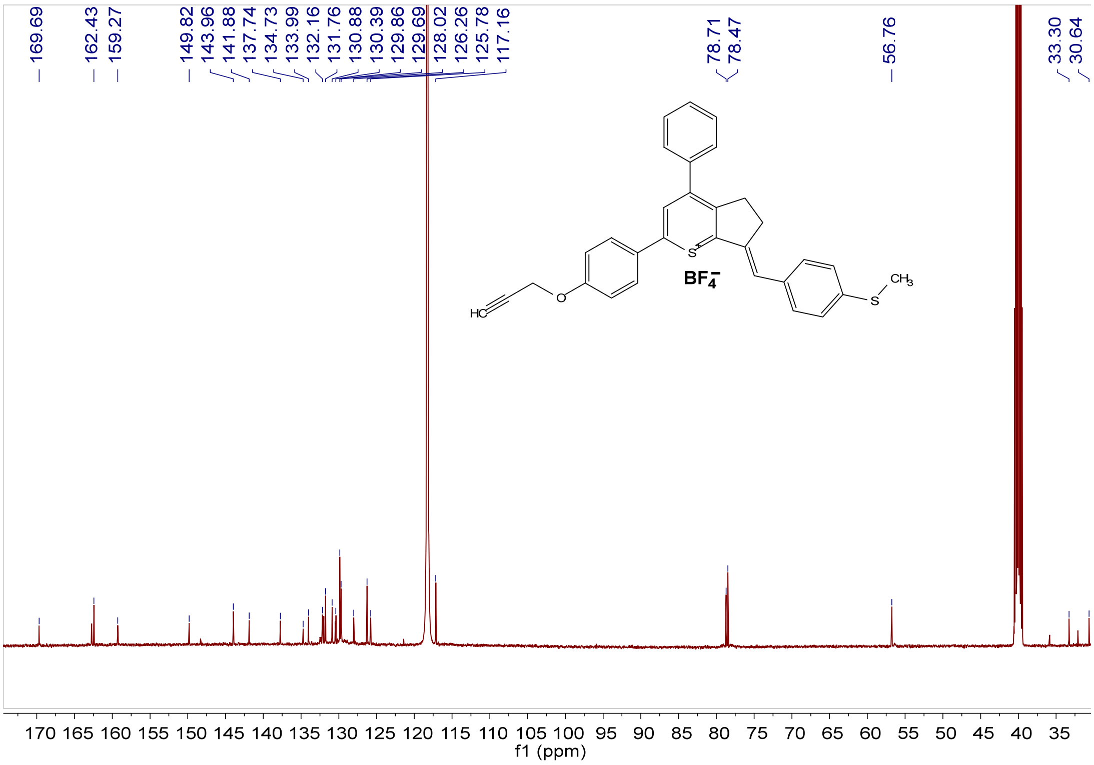

Supplementary Fig. 52 ^{13}C NMR spectra of compound **3j** in Acetonitrile-d_3 and DMSO-d_6

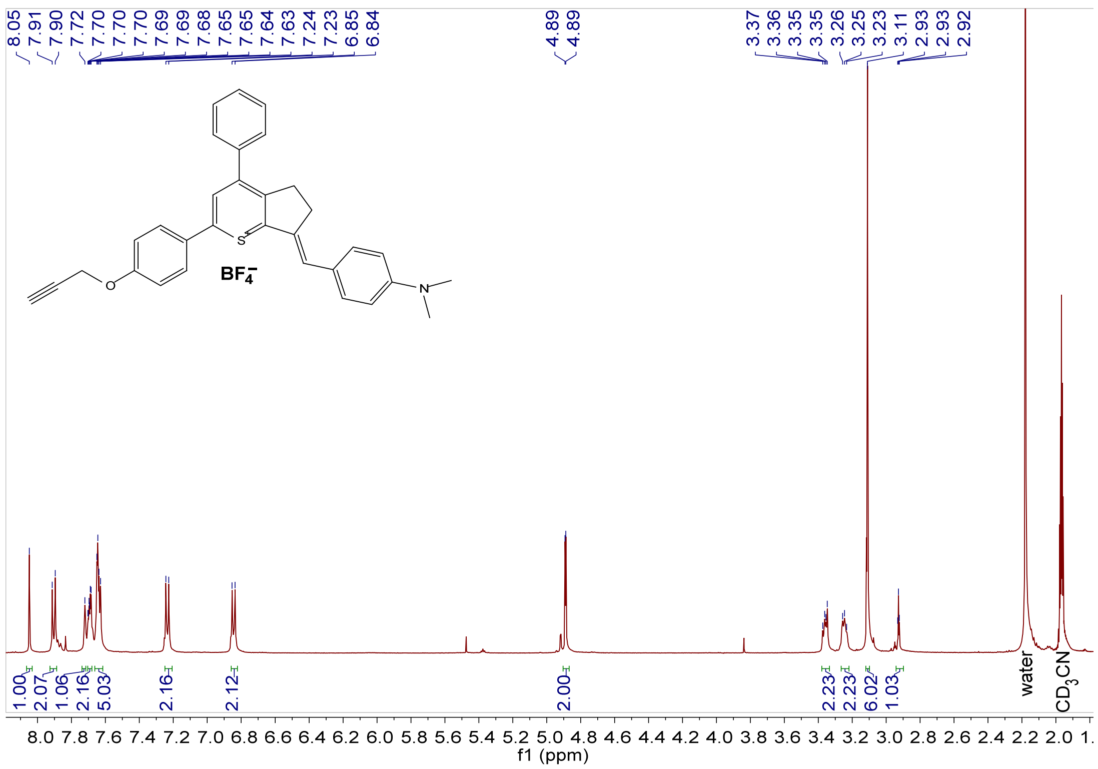

Supplementary Fig. 53 ^{1}H NMR spectra of compound **3k** in Acetonitrile-d_3.

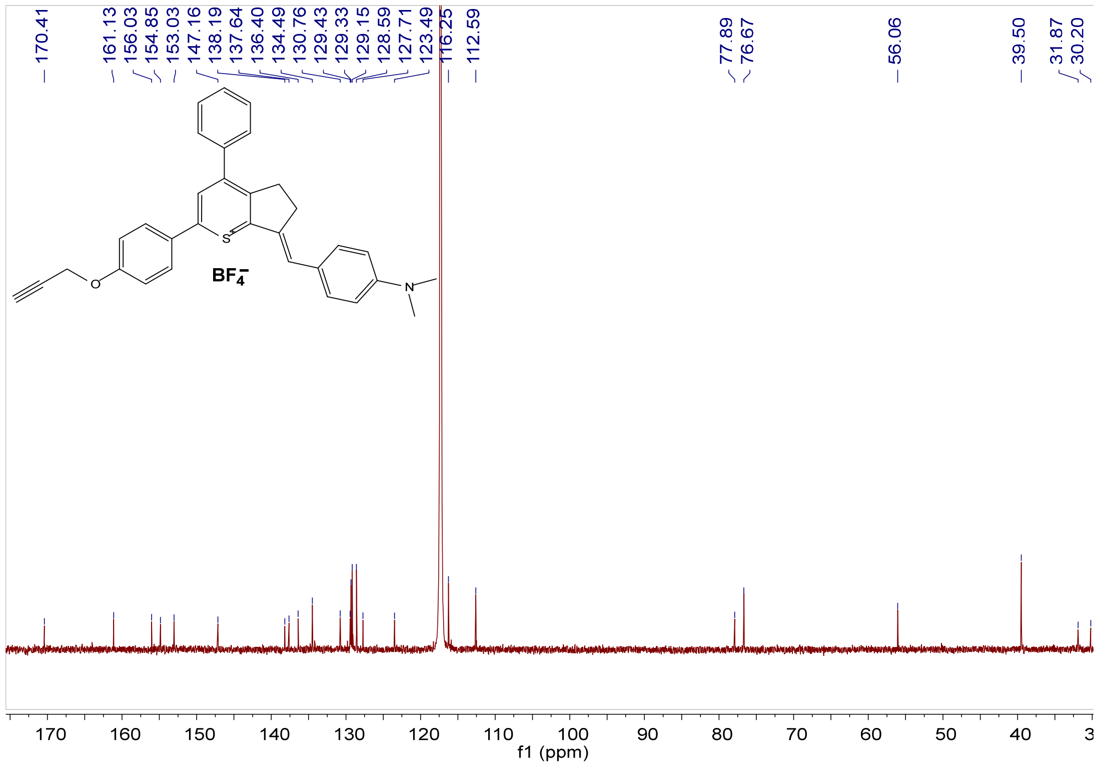

Supplementary Fig. 54 ^{13}C NMR spectra of compound **3k** in Acetonitrile-d_3.

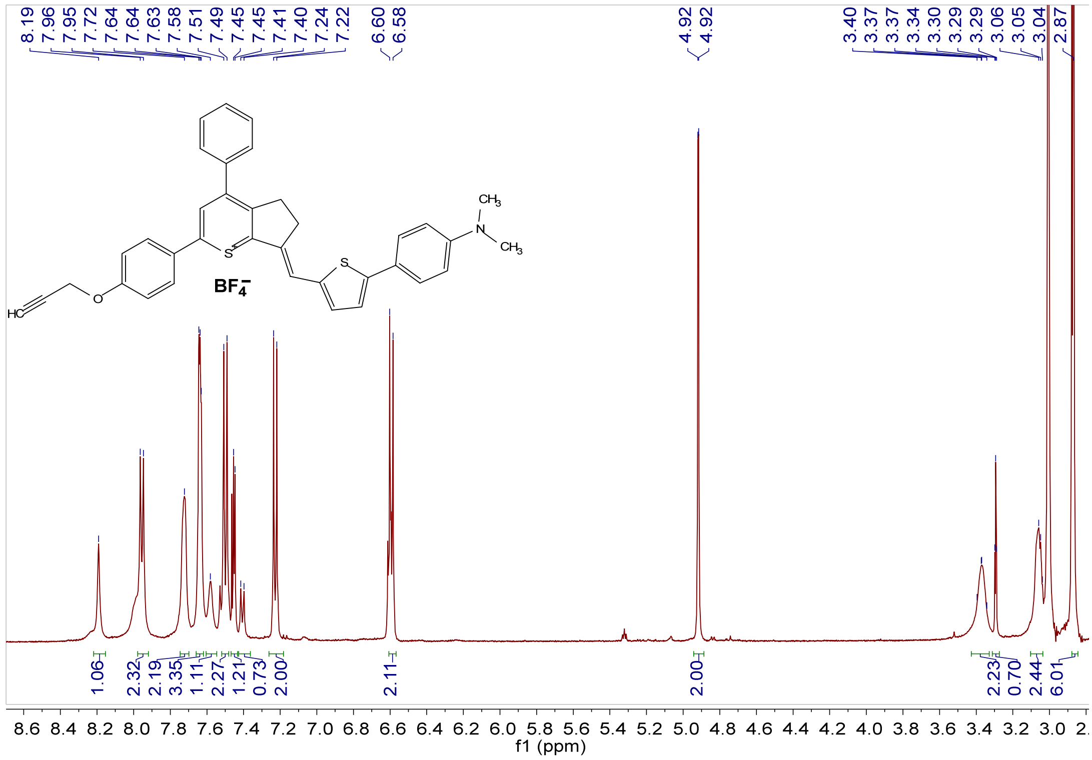

Supplementary Fig. 55 ^{1}H NMR spectra of compound **H4** in Acetonitrile-d_3 and DMSO-d_6.

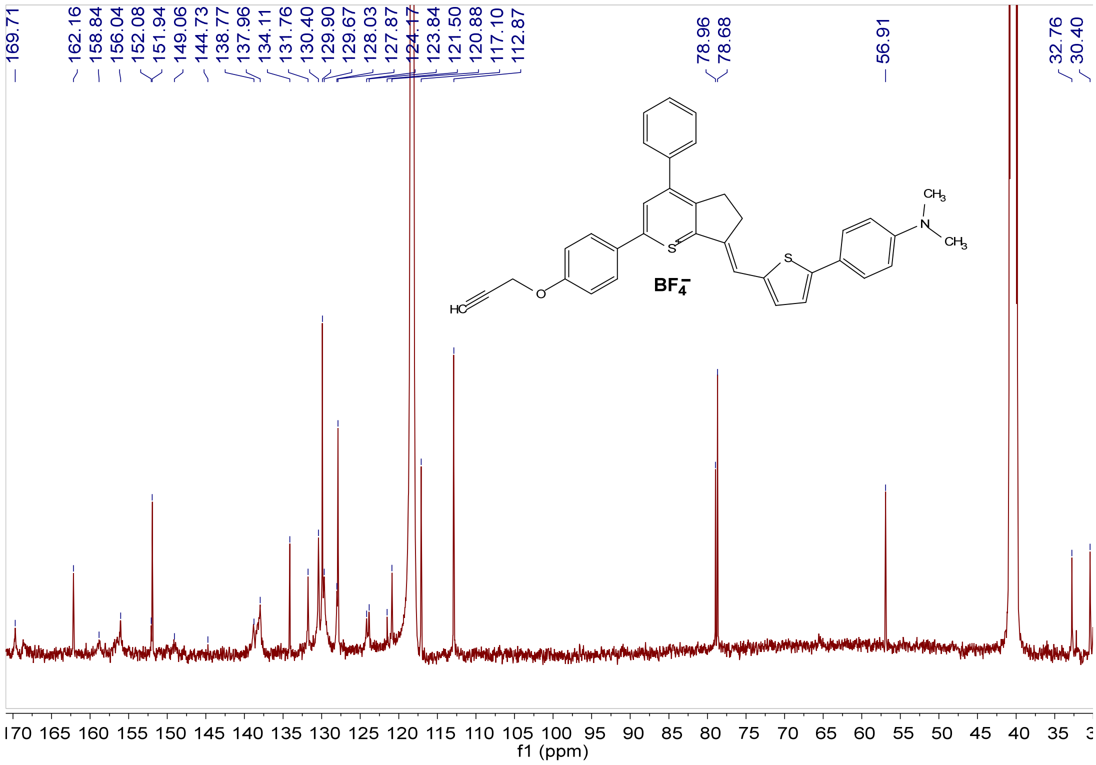

Supplementary Fig. 56 ^{13}C NMR spectra of compound **H4** in Acetonitrile-d_3 and DMSO-d_6.

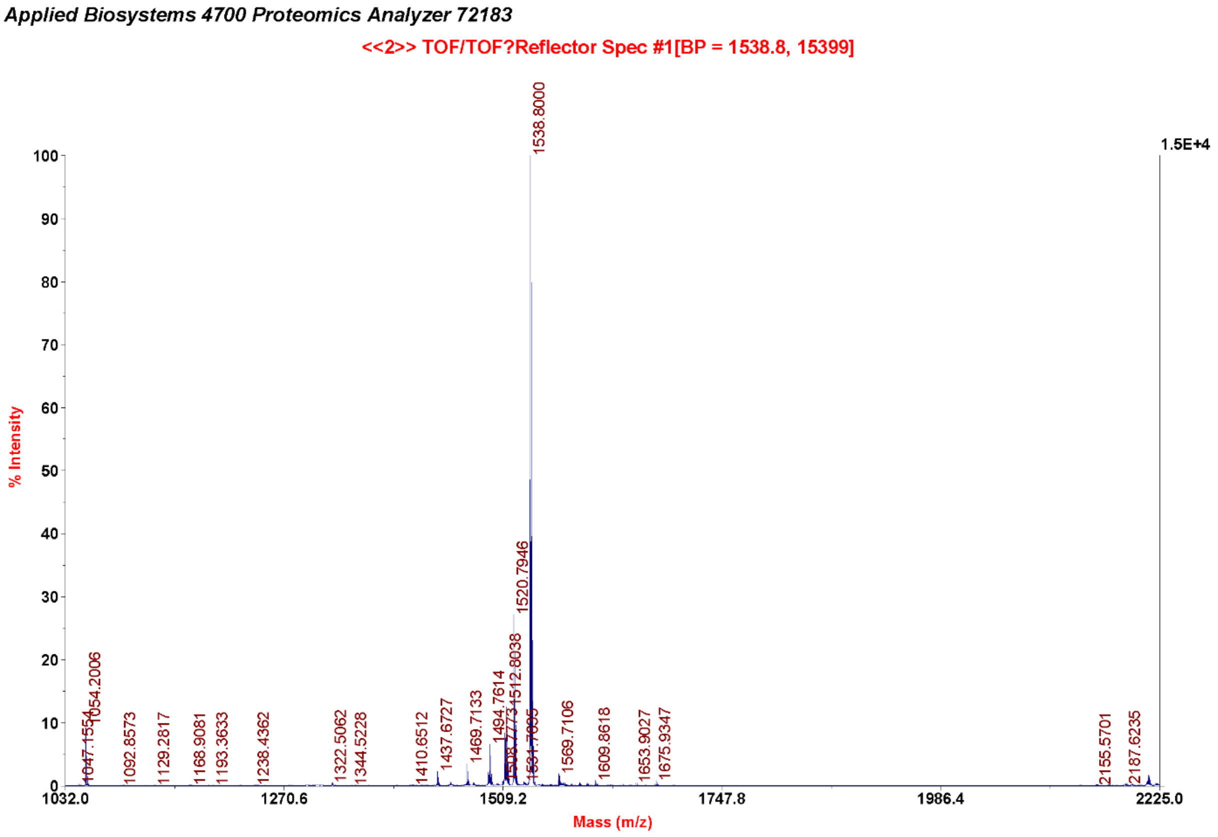

Supplementary Fig. 57 Maldi-Tof of **PEG-PT.**

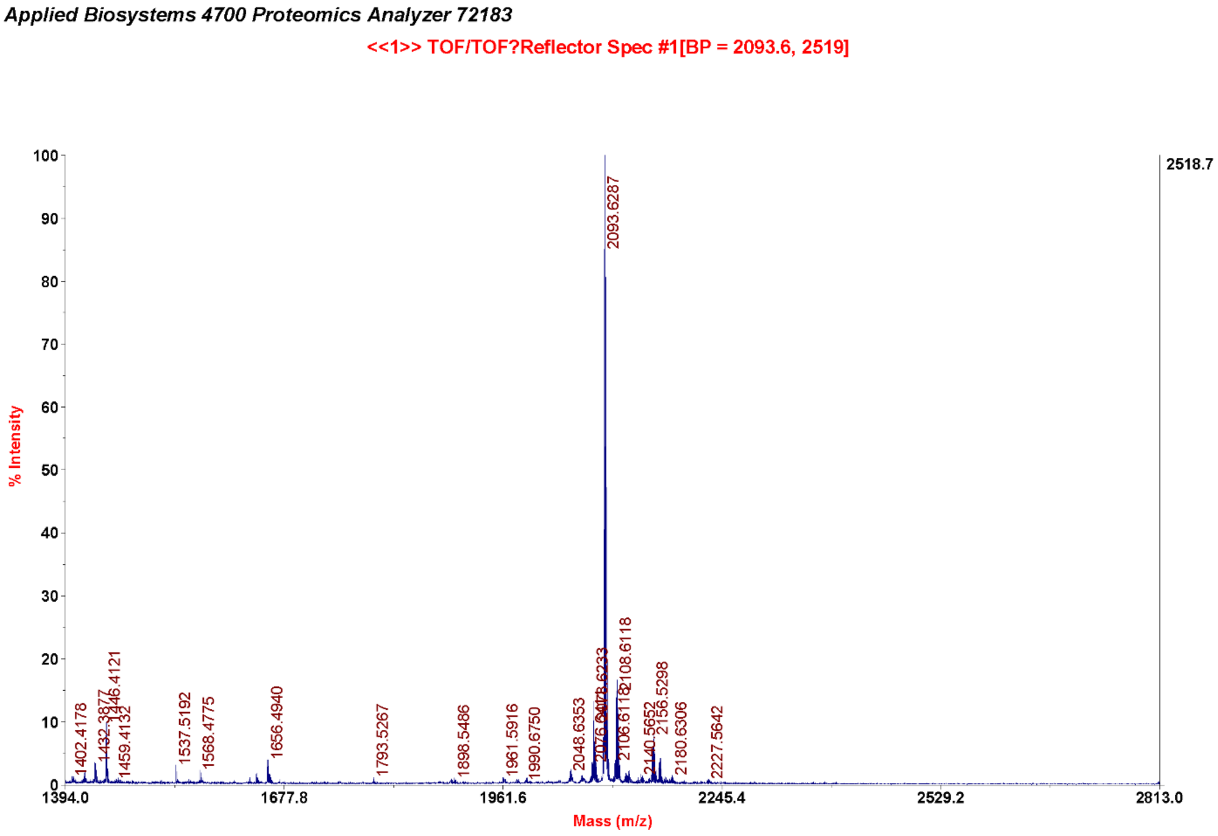

Supplementary Fig. 58 Maldi-Tof of **H4-PEG-PT.**

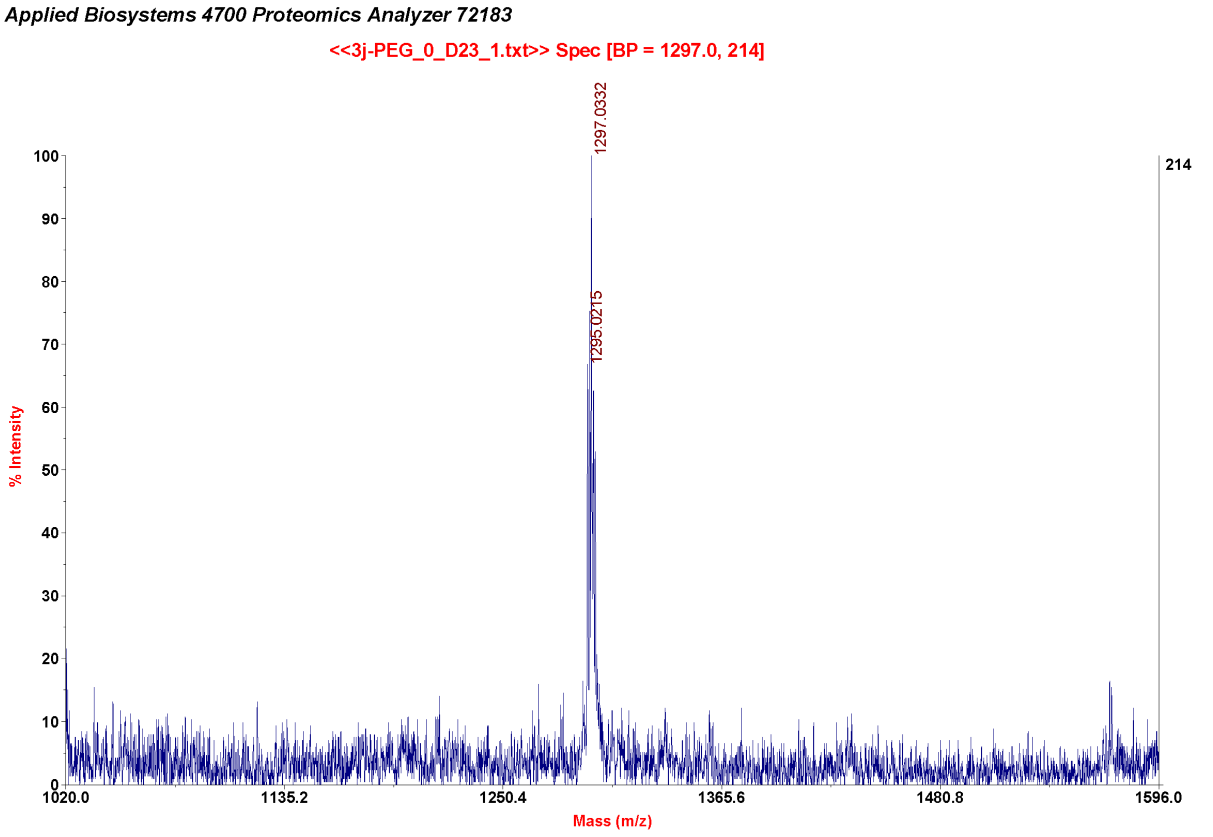

Supplementary Fig. 59 Maldi-Tof of **3j-PEG.**

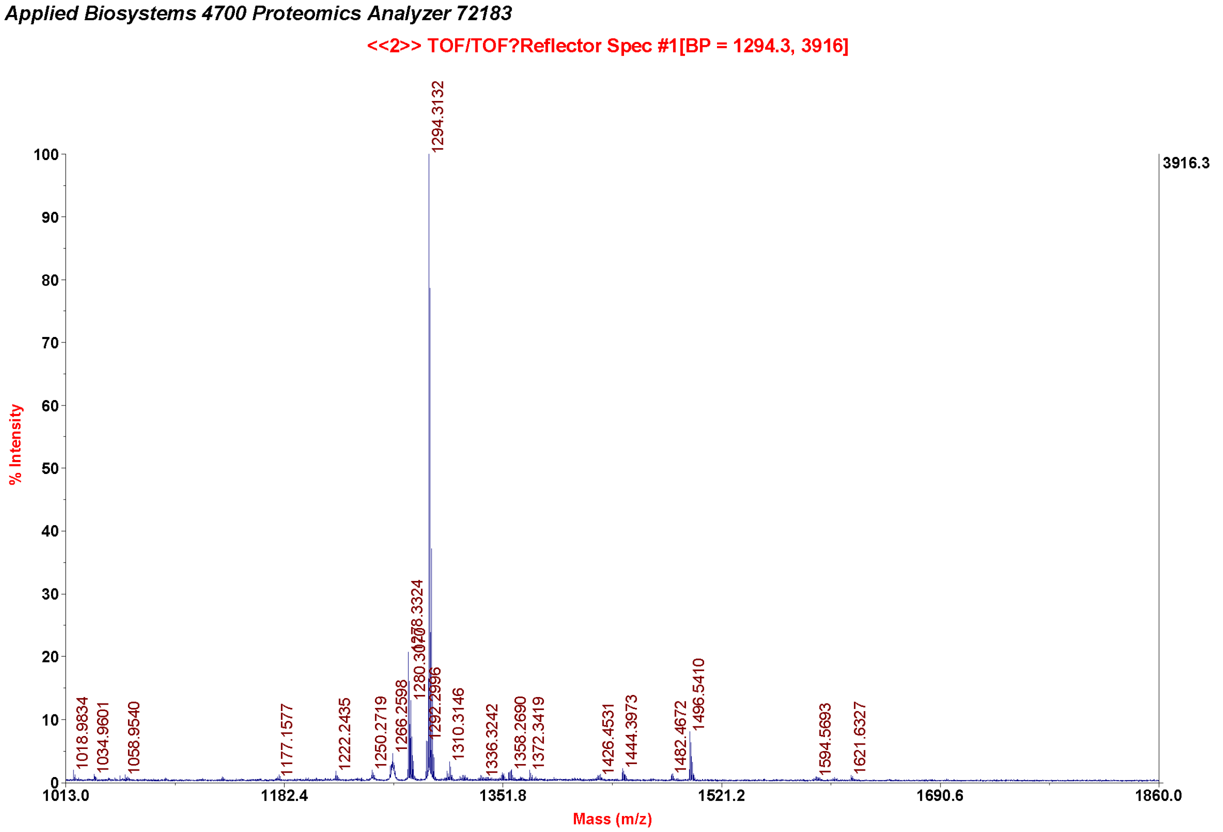

Supplementary Fig. 60 Maldi-Tof of **3k-PEG.**

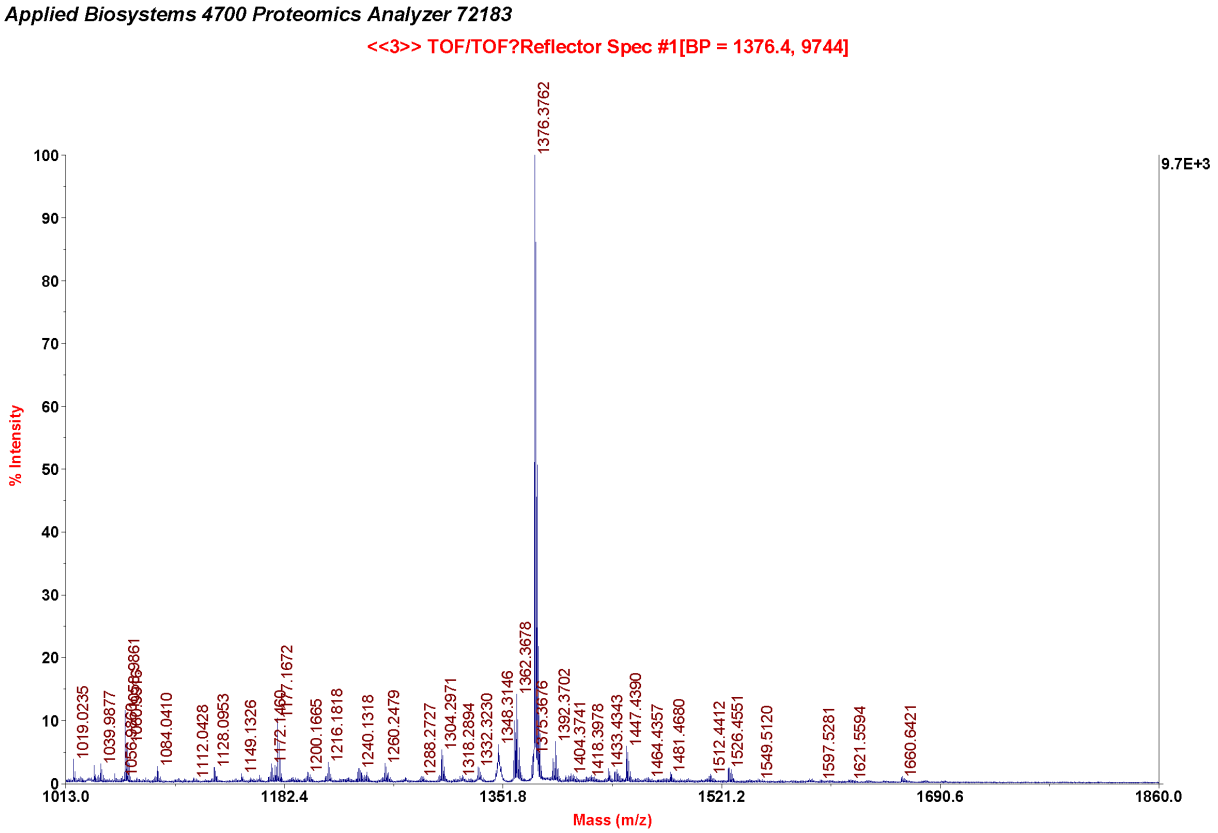

Supplementary Fig. 61 Maldi-Tof of **H4-PEG.**
