# Supplementary material for: Upconversion NIR-II fluorophores for mitochondria-targeted cancer imaging and photothermal therapy
Source: Nat Commun. 2020 Dec 3;11:6183. doi: 10.1038/s41467-020-19945-w (PMC7713230; doi:10.1038/s41467-020-19945-w)
Supplement: Supplementary file 3 — Description of Additional Supplementary Files [file 41467_2020_19945_MOESM3_ESM.docx]

**Description of Additional Supplementary Files**

**Title: Supplementary Movie 1.**

**Description: Merge of Mitotracker red and 3j-PEG**

**Title: Supplementary Movie 2.**

**Description:** **3D image (Merge of Mitotracker red and 3j-PEG)**
