# Supplementary material for: Upconversion NIR-II fluorophores for mitochondria-targeted cancer imaging and photothermal therapy
Source: Nat Commun. 2020 Dec 3;11:6183. doi: 10.1038/s41467-020-19945-w (PMC7713230; doi:10.1038/s41467-020-19945-w)
Supplement: Supplementary file 6 — Reporting Summary [file 41467_2020_19945_MOESM6_ESM.pdf]

nature research

Corresponding author(s): Xuechuan Hong, Yuling Xiao

Last updated by author(s): Oct 20, 2020

Reporting Summary

Nature Research wishes to improve the reproducibility of the work that we publish. This form provides structure for consistency and transparency in reporting. For further information on Nature Research policies, see our Editorial Policies and the Editorial Policy Checklist.

Statistics

For all statistical analyses, confirm that the following items are present in the figure legend, table legend, main text, or Methods section.

n/a	Confirmed	
☐	☒	The exact sample size (n) for each experimental group/condition, given as a discrete number and unit of measurement
☐	☒	A statement on whether measurements were taken from distinct samples or whether the same sample was measured repeatedly
☐	☒	The statistical test(s) used AND whether they are one- or two-sided<br>*Only common tests should be described solely by name; describe more complex techniques in the Methods section.*
☒	☐	A description of all covariates tested
☒	☐	A description of any assumptions or corrections, such as tests of normality and adjustment for multiple comparisons
☐	☒	A full description of the statistical parameters including central tendency (e.g. means) or other basic estimates (e.g. regression coefficient) AND variation (e.g. standard deviation) or associated estimates of uncertainty (e.g. confidence intervals)
☐	☒	For null hypothesis testing, the test statistic (e.g. F, t, r) with confidence intervals, effect sizes, degrees of freedom and P value noted<br>*Give P values as exact values whenever suitable.*
☒	☐	For Bayesian analysis, information on the choice of priors and Markov chain Monte Carlo settings
☒	☐	For hierarchical and complex designs, identification of the appropriate level for tests and full reporting of outcomes
☒	☐	Estimates of effect sizes (e.g. Cohen's d, Pearson's r), indicating how they were calculated

Our web collection on statistics for biologists contains articles on many of the points above.

Software and code

Policy information about availability of computer code

Data collection	Leica (SPE), Olympus (FLUOVIEW FV1000 and FV1200 CLSM) were used to acquire confocal images. NIR-II images were collected from NIR-II imaging system purchased from Suzhou NIR Optics technologies CO., Ltd. GFP bioluminescence images were collected from In Vivo Xtreme imaging system (XTEREME BI, Bruker). Cellular uptake and DCFH-DA assay images were obtained by Zeiss Axiovert 200 fluorescence microscopy. The absorbance intensity of MTT assay was measured by Perkin Elmer VICTOR X4. Bioluminescence or fluorescence intensity in ATP detection, JC-1 assay, MPT detection, and caspase multiplex activity assay was collected from microplate reader (TECAN, Infinite M200 Pro).UV-Vis absorbance spectrum was recorded on a PerkinElmer Lambda 25. NIR-II fluorescence spectrum was recorded on an Applied Nano Fluorescence spectrometer. MALDI-TOF mass spectra was obtained on Applied Biosystems 4700 Proteomics Analyzer. The NMR spectra were acquired on a Bruker magnetic resonance spectrometer.
Data analysis	Chemical structures were drawn by Chemdraw 14. Quantum chemistry calculation was performed using Gaussian 09 program (Revision D.09) software, Image J (1.51j8) was used to analyze the NIR-II images. Origin 9 was used to analyze the spectrum images. All statistical analyses were performed by Graphpad Prism 8.0 and SPSS 20.0.

For manuscripts utilizing custom algorithms or software that are central to the research but not yet described in published literature, software must be made available to editors and reviewers. We strongly encourage code deposition in a community repository (e.g. GitHub). See the Nature Research guidelines for submitting code & software for further information.

Data

Policy information about availability of data

All manuscripts must include a data availability statement. This statement should provide the following information, where applicable:

- Accession codes, unique identifiers, or web links for publicly available datasets
- A list of figures that have associated raw data
- A description of any restrictions on data availability

The authors declare that all data needed to evaluate the conclusion of this work are presented in the paper and the Supplementary Information. Additional data related to this paper may be requested from the authors.The source data underlying Figs. 3a-d, 4a–i, 5e–h, 8a–c, 8l–p, 8g-i, 8j-k, 9e-f and Supplementary Figs. 1a-b, 2a-h, 3a-c, 10a–c are provided as a Source Data file.

Field-specific reporting

Please select the one below that is the best fit for your research. If you are not sure, read the appropriate sections before making your selection.

[x] Life sciences   [ ] Behavioural & social sciences   [ ] Ecological, evolutionary & environmental sciences

For a reference copy of the document with all sections, see nature.com/documents/nr-reporting-summary-flat.pdf

Life sciences study design

All studies must disclose on these points even when the disclosure is negative.

Sample size	No sample size calculations were performed. Animal sample size was determined by the number of biological replicates necessary for ensuring statistical significance. The number of biological replicates are also reported in the relevant figure legends in the manuscript. For experiments other than animal studies, the sample size (n) of each experiment is provided in the figure captions in the manuscript and supplementary information accordingly. Sample sizes were chosen to support meaningful conclusions.
Data exclusions	No data were excluded.
Replication	Experiments were repeated independently at least three experiments with similar results. All experiments were reproduced to reliably support conclusions stated in the manuscript.
Randomization	All samples were randomly allocated into experimental groups.
Blinding	No studies were blinded because treatment was administered by the researcher collecting the data.

Reporting for specific materials, systems and methods

We require information from authors about some types of materials, experimental systems and methods used in many studies. Here, indicate whether each material, system or method listed is relevant to your study. If you are not sure if a list item applies to your research, read the appropriate section before selecting a response.

Materials & experimental systems

n/a	Involved in the study	
[ ]	[x]	Antibodies
[ ]	[x]	Eukaryotic cell lines
[x]	[ ]	Palaeontology and archaeology
[ ]	[x]	Animals and other organisms
[x]	[ ]	Human research participants
[x]	[ ]	Clinical data
[x]	[ ]	Dual use research of concern

Methods

n/a	Involved in the study	
[x]	[ ]	ChIP-seq
[x]	[ ]	Flow cytometry
[x]	[ ]	MRI-based neuroimaging

Antibodies

Antibodies used	Recombinant Anti-Smac/Diablo antibody [Y12] (ab32023,abcam); Anti-Endo G antibody (ab9647, abcam); Recombinant Anti-AIF antibody [E20] - Mitochondrial Marker (ab32516, abcam); Recombinant Anti-Cytochrome C antibody [EPR1327] (ab133504, abcam); Secondary Alexa-647-conjugated Goat anti-Rabbit Antibody (A27040, Life technologies); HRP conjugated Goat Anti-Rabbit IgG H&L (HRP) (ab6721, abcam); Anti-caspase 3, anti-cleaved caspase 3, anti-cleaved caspase 9 and anti-caspase 9: (Cell Signaling Technology, 9662S, 9664S, 9509S and 9504S respectively); anti-β-actin antibody (ab179467, abcam).
Validation	All antibodies were verified by the supplier and each lot has been quality tested.

Eukaryotic cell lines

Policy information about cell lines

Cell line source(s)	L929 and HepG2 cells were purchased from the China Center for Type Culture Collection (CCTCC). 143 B cells were purchased from American Type Culture Collection (ATCC).
Authentication	All cell lines were authenticated by Short Tandem Repeat test.
Mycoplasma contamination	All cell lines tested negative for mycoplasma contamination.
Commonly misidentified lines (See ICLAC register)	No commonly misidentified cell lines were used in the study.

Animals and other organisms

Policy information about studies involving animals; ARRIVE guidelines recommended for reporting animal research

Laboratory animals	Female Balb/c nude mice (6 weeks old) were obtained from Beijing Vital River Laboratory Animal Technology Co., Ltd. Mice were housing in independent ventilation cage (IVC) system under specific pathogen free (SPF) condition. Housing temperature was 22-25 Celsius degree, humidity was 35-45%. The dark/light cycle was 12 h light/12 h dark.
Wild animals	The study did not involve wild animals.
Field-collected samples	The study did not involve samples collected from field.
Ethics oversight	All animal studies were performed in accordance with the Guidelines for the Care and Use of Laboratory Animals of the Chinese Animal Welfare Committee and approved by The Institutional Animal Care and Use Committee (IACUC), Wuhan University Center for Animal Experiment, Wuhan, China.

Note that full information on the approval of the study protocol must also be provided in the manuscript.
